# N^+^-C-H···O Hydrogen bonds in protein-ligand complexes

**DOI:** 10.1038/s41598-018-36987-9

**Published:** 2019-01-25

**Authors:** Yukihiro Itoh, Yusuke Nakashima, Shuichiro Tsukamoto, Takashi Kurohara, Miki Suzuki, Yoshitake Sakae, Masayuki Oda, Yuko Okamoto, Takayoshi Suzuki

**Affiliations:** 1 Department of Chemistry, Graduate School of Medical Science, Kyoto Prefectural University of Medicine, 1-5 Shimogamohangi-cho, Sakyo-ku, Kyoto, 606-0823 Japan; 2 Department of Physics, Nagoya University, Furo-cho, Chikusa-ku, Nagoya, Aichi 464-8602 Japan; 3 Graduate School of Life and Environmental Sciences, Kyoto Prefectural University, Kyoto, 1-5 Shimogamohangi-cho, Sakyo-ku, Kyoto, 606-8522 Japan; 4 CREST, Japan Science and Technology Agency (JST), 4-1-8 Honcho, Kawaguchi, Saitama, 332-0012 Japan

## Abstract

In the context of drug design, C-H···O hydrogen bonds have received little attention so far, mostly because they are considered weak relative to other noncovalent interactions such as O-H···O hydrogen bonds, π/π interactions, and van der Waals interactions. Herein, we demonstrate the significance of hydrogen bonds between C-H groups adjacent to an ammonium cation and an oxygen atom (N^+^-C-H···O hydrogen bonds) in protein-ligand complexes. Quantum chemical calculations revealed details on the strength and geometrical requirements of these N^+^-C-H···O hydrogen bonds, and a subsequent survey of the Protein Data Bank (PDB) based on these criteria suggested that numerous protein-ligand complexes contain such N^+^-C-H···O hydrogen bonds. An ensuing experimental investigation into the G9a-like protein (GLP)-inhibitor complex demonstrated that N^+^-C-H···O hydrogen bonds affect the activity of the inhibitors against the target enzyme. These results should provide the basis for the use of N^+^-C-H···O hydrogen bonds in drug discovery.

## Introduction

Noncovalent interactions, such as heteroatom-hydrogen bonds X-H···Y (X = O or N; Y = O, N, or halogen) and π/π interactions play a critical role in the formation of protein-ligand complexes^1–6^. Such interactions manifest between proteins and their ligands in many protein complexes registered on the Protein Data Bank (PDB). Accordingly, these interactions should always be considered when designing ligands for target proteins^7^.

Although C-H···O hydrogen bonds are also noncovalent interactions, their potential significance in the context of drug design has received little attention so far, which is probably due to the fact that they are considered weaker than heteroatom-hydrogen bonds^8–11^. However, when the C-H group is activated by electron-withdrawing groups, e.g. C-H groups that are covalently bound to a cationic nitrogen atom (N^+^-C-H), C-H···O hydrogen bonds may become as strong as heteroatom-hydrogen bonds, which could be important for molecular recognition^12–19^. For example, N^+^-C-H···Y hydrogen bonds are likely to be involved in the substrate recognition of tetraalkylammonium-based catalysts^20,21^, and the ligand/substrate recognition in receptors/enzymes may be controlled by N^+^-C-H···O hydrogen bonds (Supplementary Fig. S1A,B)^22–24^. Based on these findings, we envisioned that C-H···O hydrogen bonds that are activated by ammonium cations should represent in general important interactions for protein-ligand interactions and drug design. Many small-molecule ligands for proteins contain tetraalkylammonium or aliphatic amino groups. Given their p*K*_aH_ values (~10), the latter are almost exhaustively protonated under physiological conditions (pH = 7–8). Accordingly, ligands bearing tetraalkylammonium or aliphatic amino groups could be recognized by the oxygen atoms of the proteins via N^+^-C-H···O hydrogen bonds (Supplementary Fig. S1C,D). In this study, we attempted to examine the importance of N^+^-C-H···O hydrogens in protein-ligand interactions by a threefold approach: (i) theoretical research and criteria setting for N^+^-C-H···O hydrogen bonds by quantum chemical calculations (Table 1), (ii) a PDB survey based on these criteria, and (iii) an experimental investigation. Herein, we describe the details and results of our study on N^+^-C-H···O hydrogen bonds.

**Table 1:** Summary for the geometric profiling of N^+^-C-H···O hydrogen bonds.

Hydrogen acceptor	Geometry	Schematic diagram	Range	Preference^*a*^
Peptide bond/Asn/Gln	H···O distance (*d*_HO_)		2.0 Å < *d*_HO_ < 2.7 Å	<2.4 Å
	C-H···O angle (*ψ*)		90° < *ψ* < 180°	
	H···O=C angle (*ξ*)		105° < *ξ* < 180°	>135°
	H-elevation angle (*θ*)		<70°	~0°
Asp/Glu	*d*_HO_		1.4 Å < *d*_HO_ < 2.7 Å	<2.4 Å
	*ψ*		90° < *ψ* < 180°	
	*ξ*		90° < *ξ* < 270°	>240°
	*θ*		<80°	
Ser/Thr/Tyr	*d*_HO_		1.8 Å < *d*_HO_ < 2.7 Å (Ser/Thr) 2.0 Å < *d*_HO_ < 2.7 Å (Tyr)	<2.4 Å
	*ψ*		105° < *ψ* < 180°	
	*ξ*		105° < *ξ* < 150°	105°–120°
	*θ*		<60° (Ser/Thr) <75° (Tyr)	~0°

^*a*^See Supplementary Fig. S9.

## Results and Discussion

### Establishment of criteria for the presence of N^+^-C-H···O hydrogen bonds in protein-ligand complexes based on quantum chemical calculations

Before N^+^-C-H···O hydrogen bonds can be examined in protein-ligand complexes, selection criteria have to be established for such interactions, as general criteria to determine the existence of N^+^-C-H···O hydrogen bonds in protein-ligand complexes still remain elusive. Although a few computational studies on N^+^-C-H···O hydrogen bonds have previously been reported^12,17^, the quantity of theoretical information on N^+^-C-H···O hydrogen bonds remains low, especially regarding the character of the hydrogen acceptor oxygen atoms and the relationship between the energy and the geometry of these interactions. In this study, we simulated complexes of two molecules bound via N^+^-C-H···O hydrogen bonds, and we determined the strength and geometry of the N^+^-C-H···O hydrogen bonds between proteins and ligands. Specifically, we used *N*-methylacetamide (**1**), propanoate (**2**), ethanol (**3**), and phenol (**4**) as hydrogen acceptor models for the protein peptide bond/Asn/Gln, Asp/Glu, Ser/Thr, and Tyr, respectively, and monomethylammonium (**5**), trimethylammonium (**6**) or *N*-methylpiperidium (**7**) as hydrogen donor models in protonated aliphatic amine-containing ligands. Geometry optimizations and single-point calculations were carried out at the M06-2X/6-311++G** level of theory^25,26^. The results of this computational study allowed us to establish selection criteria for N^+^-C-H···O hydrogen bonds (Table 1).

Initially, we analyzed the electrical charges on the hydrogen donors of the N^+^-C-H···O hydrogen bond models, considering that the electrostatic force controls the geometry of the heteroatom-hydrogen bonds^27^. A natural bond orbital (NBO) analysis^28^ of **5** indicated that the positive charge is distributed over the H atoms (formal charge on H1: 0.237; H2: 0.447), including those of the CH_3_ group, rather than being localized on the N atom (−0.688) (Supplementary Fig. S2). The NBO analysis also showed that the formal charge on the H atoms of the CH_3_ group in **5** (0.237) is more positive than that of monomethylamine (**8**; 0.185/0.159) or ethane (**9**; 0.195). These results suggest high potential for the N^+^-C-H group to act as a hydrogen donor in hydrogen bonds.

**Figure 1:**
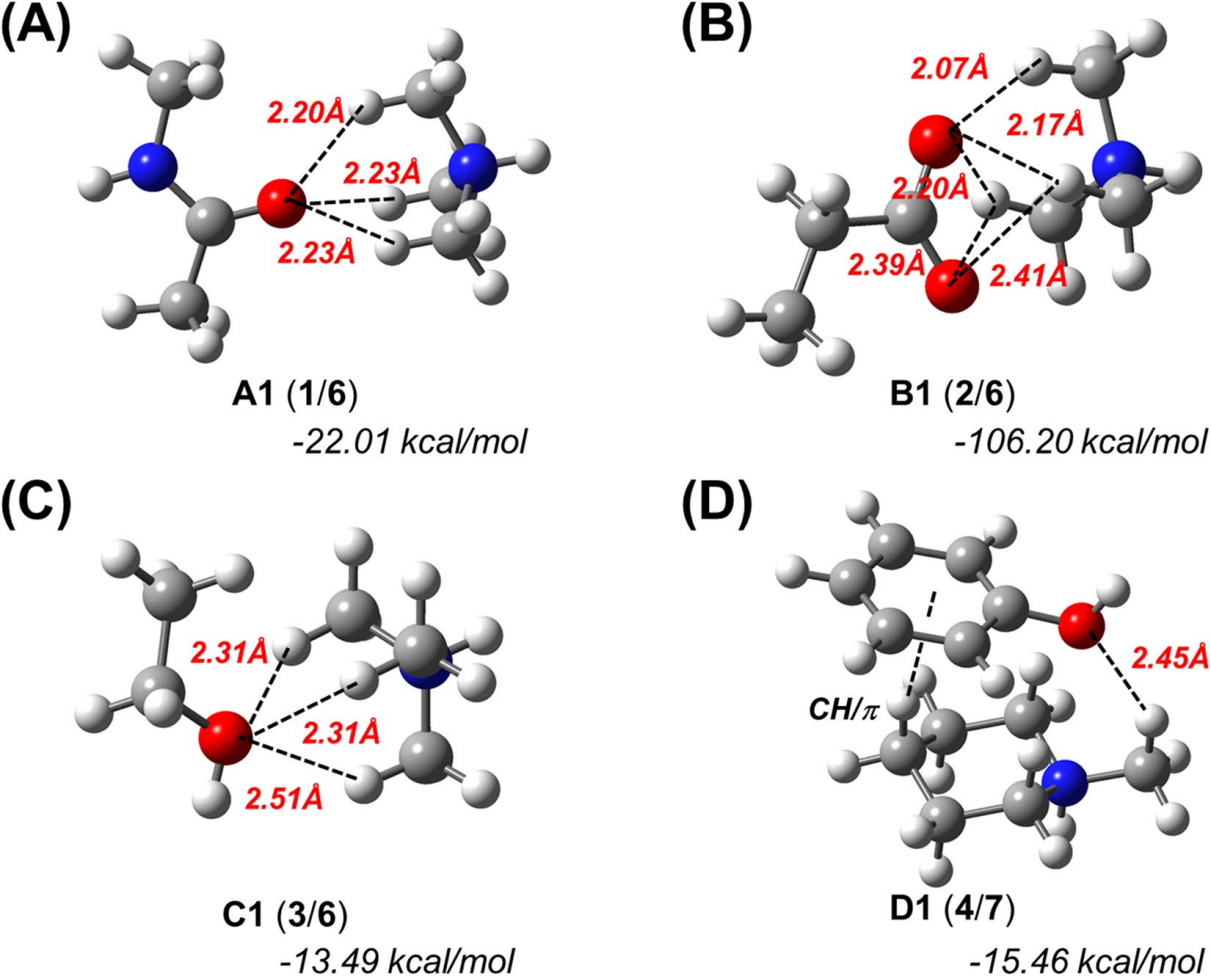
Theoretically optimized geometries and counterpoise-corrected interaction energies for N^+^-C-H··O hydrogen bond models. The geometry optimizations and energy calculations were carried out at the M06-2X/6-311++G** level of theory in the gas phase. (**A**) Trimethylammonium (**6**) complexed with *N*-methylacetamide (**1**). (**B**) **6** complexed with propanoate (**2**). (**C**) **6** complexed with ethanol (**3**). (**D**) *N*-methylpiperidium (**7**) complexed with phenol (**4**).

Subsequently, we simulated the optimized structures of complexes **A1**, **B1** and **C1**, i.e., complexes of **1**, **2**, and **3** with **6**, as well as that of complex **D1**, which consists of **4** and **7** in the gas phase (Fig. 1). For the optimized complex between **4** with **6**, only CH/π interactions were calculated, which is comparable to the results of a previously reported similar model of phenol/SMeEt_2_^+^ (Supplementary Fig. S3)^16^. The H···O distances between one H atom of the CH_3_ groups and one O atom of each hydrogen acceptor are less than the sum of the van der Waals radii of hydrogen and oxygen (2.72 Å), which suggests the formation of intermolecular hydrogen bonds between these atoms (Fig. 1 and Table S1). In addition, counterpoise-corrected interaction energies^29^ of −22.01, −106.20, −13.49 and −15.46 kcal/mol were calculated for **A1**, **B1**, **C1**, and **D1**, respectively. Subsequently, we also simulated the optimized structures of complexes **A1**–**D1** in water (Supplementary Fig. S4 and Table S1). Although the interaction energies/H···O distances in water were slightly higher/longer than those in the gas phase, we did not observe any significant differences between the two phases. These results suggest that the results in the gas phase should at least qualitatively correlate with those in water or a ligand-binding pocket, as the latter does generally not contain many water molecules. Therefore, we thereafter performed all calculations in the gas phase.

Next, we compared energies of the N^+^-C-H···O hydrogen bonds to those of interactions which are often considered in drug design. As a result, the energies of the N^+^-C-H···O hydrogen bonds are low relative to those of heteroatom-hydrogen bonds, π/π interactions, cation/π interactions, or CH/π interaction models (Supplementary Fig. S5).

Subsequently, we compared **A1**–**D1** with **A2**–**D2** and **A3**–**D3**, in which **6** in **A1**–**C1** is replaced with trimethylamine (**10**) or 2,2-dimethylpropane (**11**), and **7** in **D1** is replaced with *N*-methylpiperidine (**12**) or methyl cyclohexane (**13**) (Supplementary Fig. S6 and Table S1). The conformations of **A2** and **A3** are significantly different from that of **A1** (Supplementary Fig. S6A). The H···O distances in **A2**–**D2** and **A3**–**D3** are longer than those in **A1**–**D1** (Fig. 1, Supplementary Fig. S6, and Table S1). Additionally, the interaction energies of **A1**–**D1** are lower than those of **A2**–**D2** and **A3**–**D3** (Fig. 1, Supplementary Fig. S6, and Table S1). These results indicate that the nitrogen cation is important for the formation of the C-H···O hydrogen bond, as well as for its strength. Finally, we also estimated the influence of an N-H···O hydrogen bond formed involving the ammonium moiety on the C-H···O hydrogen bonds of **A1**–**D1** (Supplementary Fig. S7 and Table S1). For these simulations, we used a water molecule as a hydrogen-bond acceptor for the N-H···O hydrogen bond. The obtained results suggest that even if ideal heteroatom-hydrogen bonds are formed between the ammonium cation and the water molecule, the geometries and energies of the C-H···O hydrogen bonds of **A1**–**D1** are hardly influenced by the heteroatom-hydrogen bonds.

**Figure 2:**
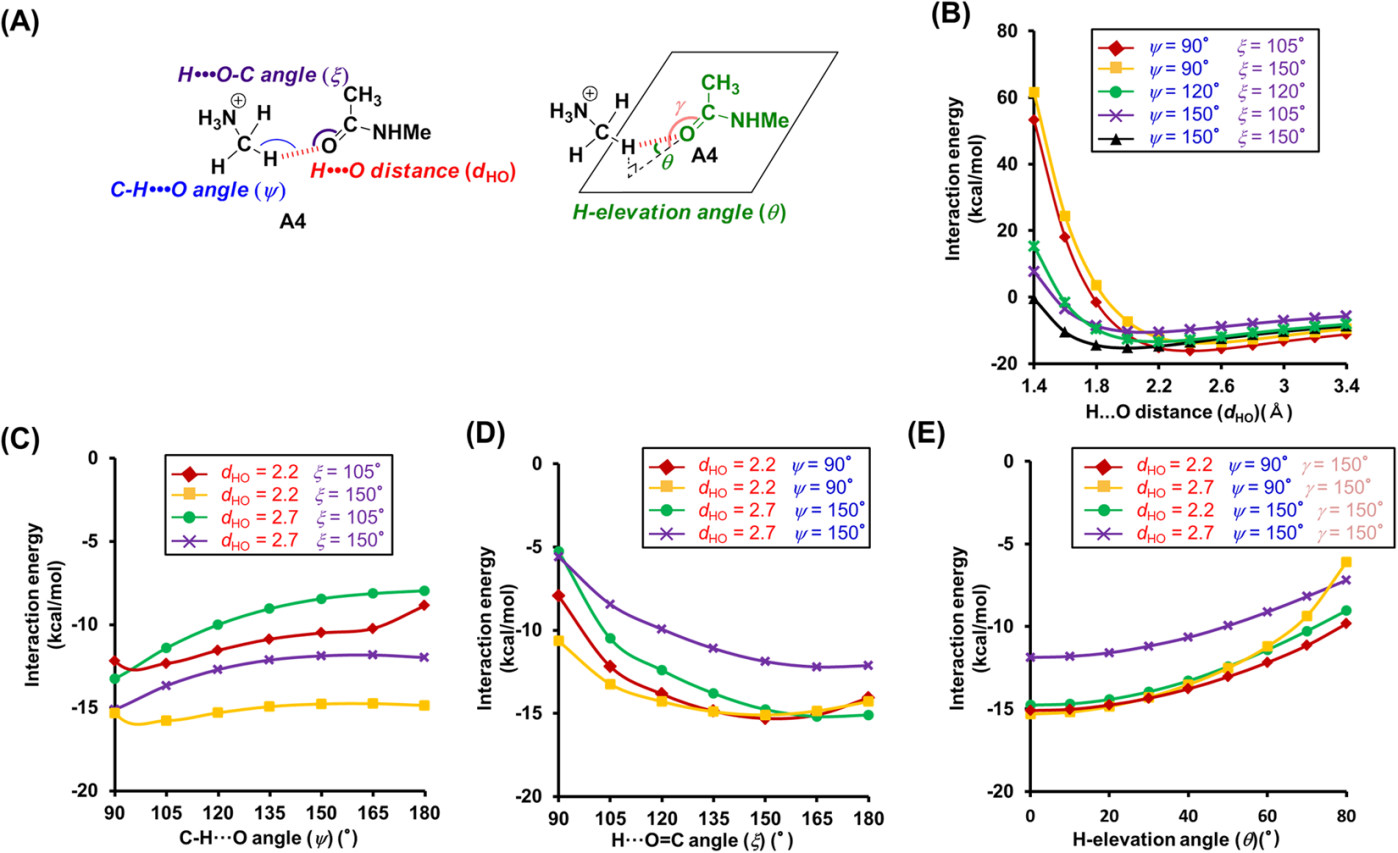
Theoretical analysis of the dependence of the interaction energies on the geometry of the N^+^-C-H···O hydrogen bonds between *N*-methylacetamide (**1**) and monomethylamine (**5**). (**A**) Geometry of the N^+^-C-H···O hydrogen bond model between **1** and **5**. (**B**–**E**) Interaction energies of the N^+^-C-H···O hydrogen bond as a function of the following geometry parameters. (**B**) H···O distance (*d*_HO_). (**C**) C-H···O angle (*ψ*). (**D**) H···O=C angle (*ξ*). (**E**) H-elevation angle (*θ*). For the analysis of the H···O distance, the C-H···O, H···O=C, and H-elevation angles were kept constant (0°). Interaction energies were corrected for basis set superposition error (BSSE) by counterpoise correction.

**Figure 3:**
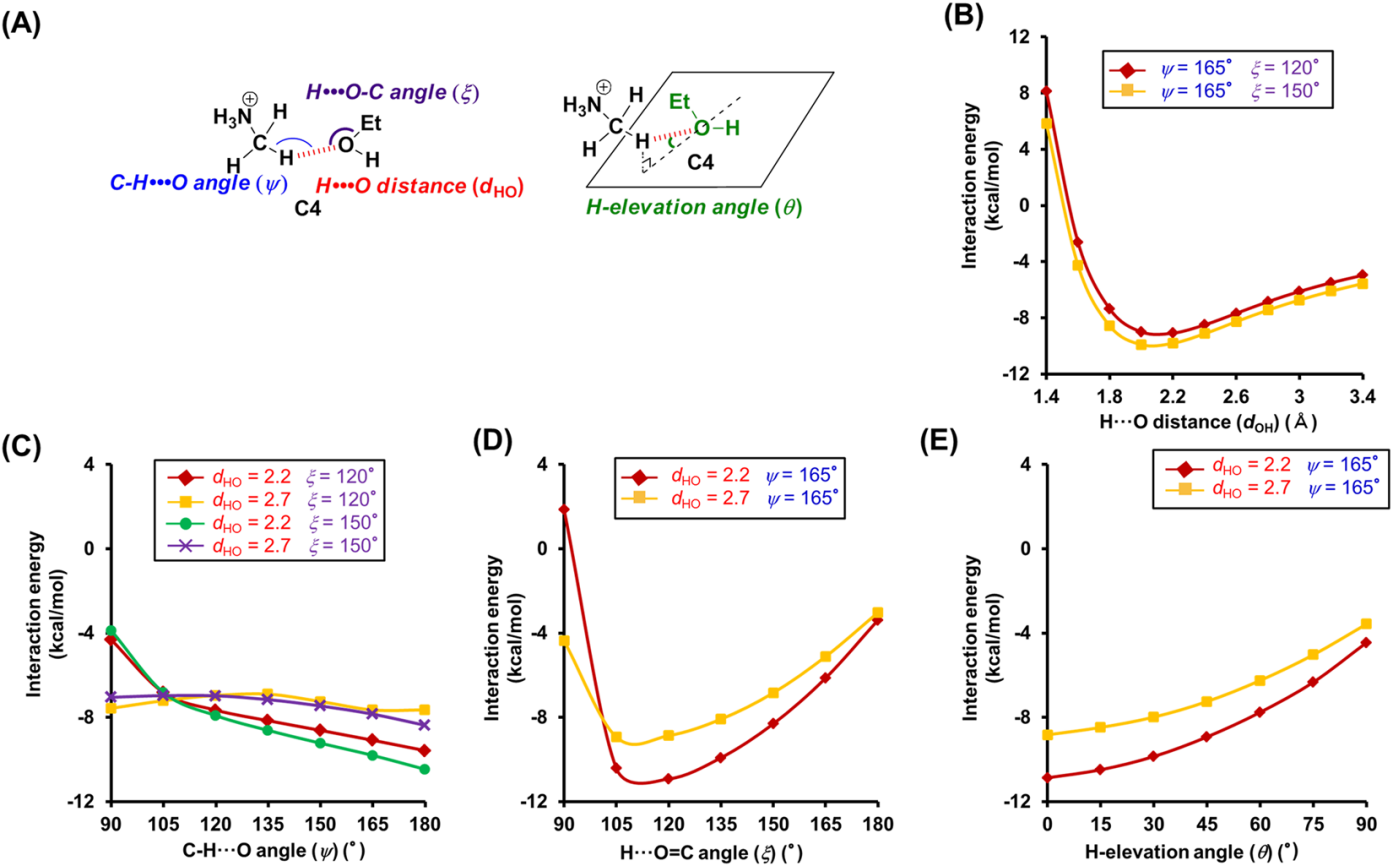
Theoretical analysis of the dependence of the interaction energies on the geometry of N^+^-C-H···O hydrogen bonds between propanoate (**2**) and monomethylamine (**5**). (**A**) Geometry of the N^+^-C-H···O hydrogen bond model between **2** and **5**. (**B**–**E**) Interaction energies of the N^+^-C-H···O hydrogen bond as a function of the following geometry parameters. (**B**) H···O distance (*d*_HO_). (**C**) -CH···O angle (*ψ*). (**D**) H···O=C angle (*ξ*). (**E**) H-elevation angle (*θ*). For the analysis of the H···O distance, the C-H···O, H···O=C, and H-elevation angles were kept constant (0°). Interaction energies were corrected for BSSE by counterpoise correction.

**Figure 4:**
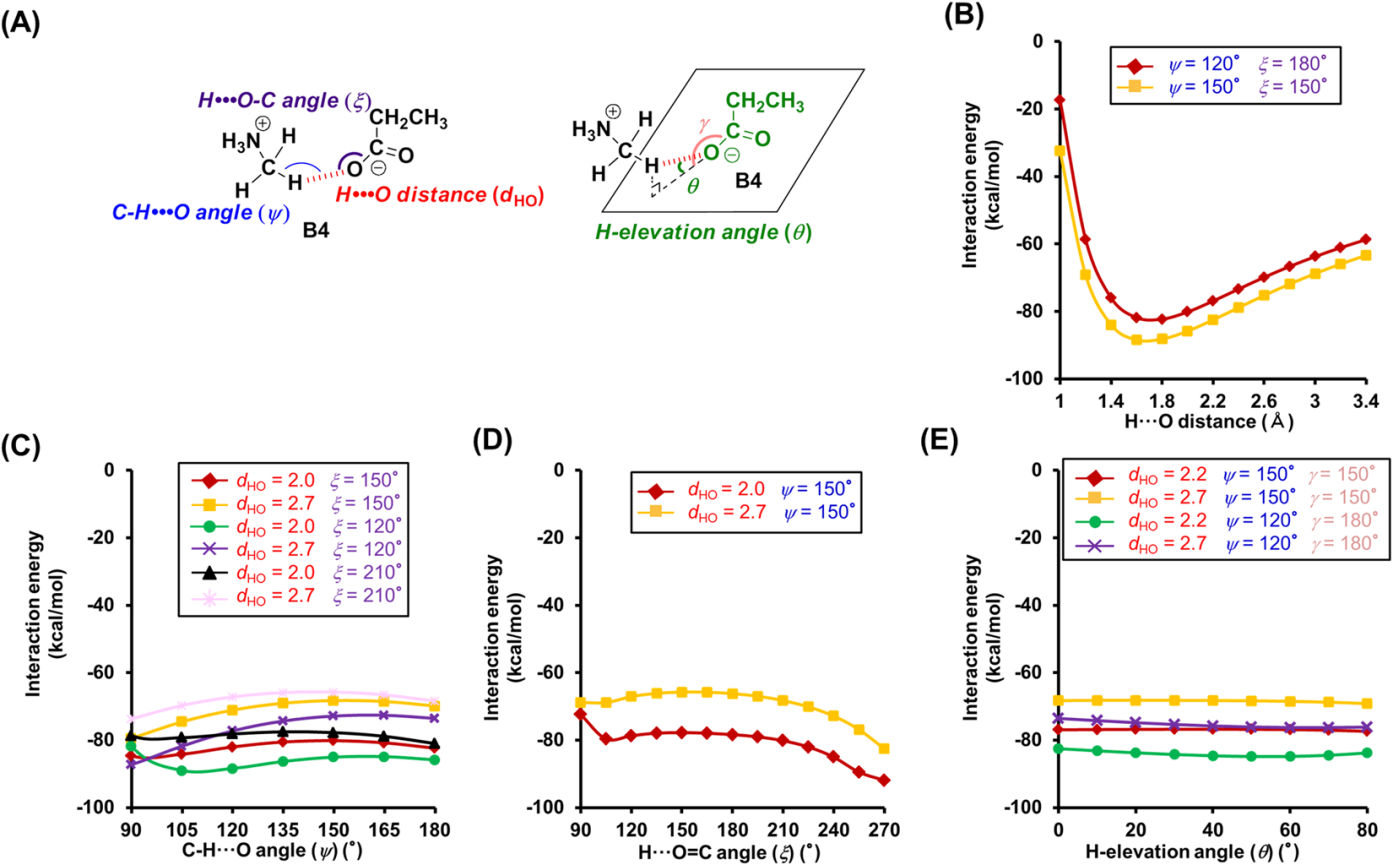
Theoretical analysis of the dependence of the interaction energies on the geometry of the N^+^-C-H···O hydrogen bonds between ethanol (**3**) and monomethylamine (**5**). (**A**) Geometry of the N^+^-C-H···O hydrogen bond model between **3** and **5**. (**B**–**E**) Interaction energies of the N^+^-C-H···O hydrogen bond as a function of the following geometry parameters. (**B**) H···O distance (*d*_HO_). (**C**) C-H···O angle (*ψ*). (**D**) H···O=C angle (*ξ*). (**E**) H-elevation angle (*θ*). For the analysis of the H···O distance, the C-H···O, H···O=C, and H-elevation angles were kept constant (0°). Interaction energies were corrected for BSSE by counterpoise correction.

**Figure 5:**
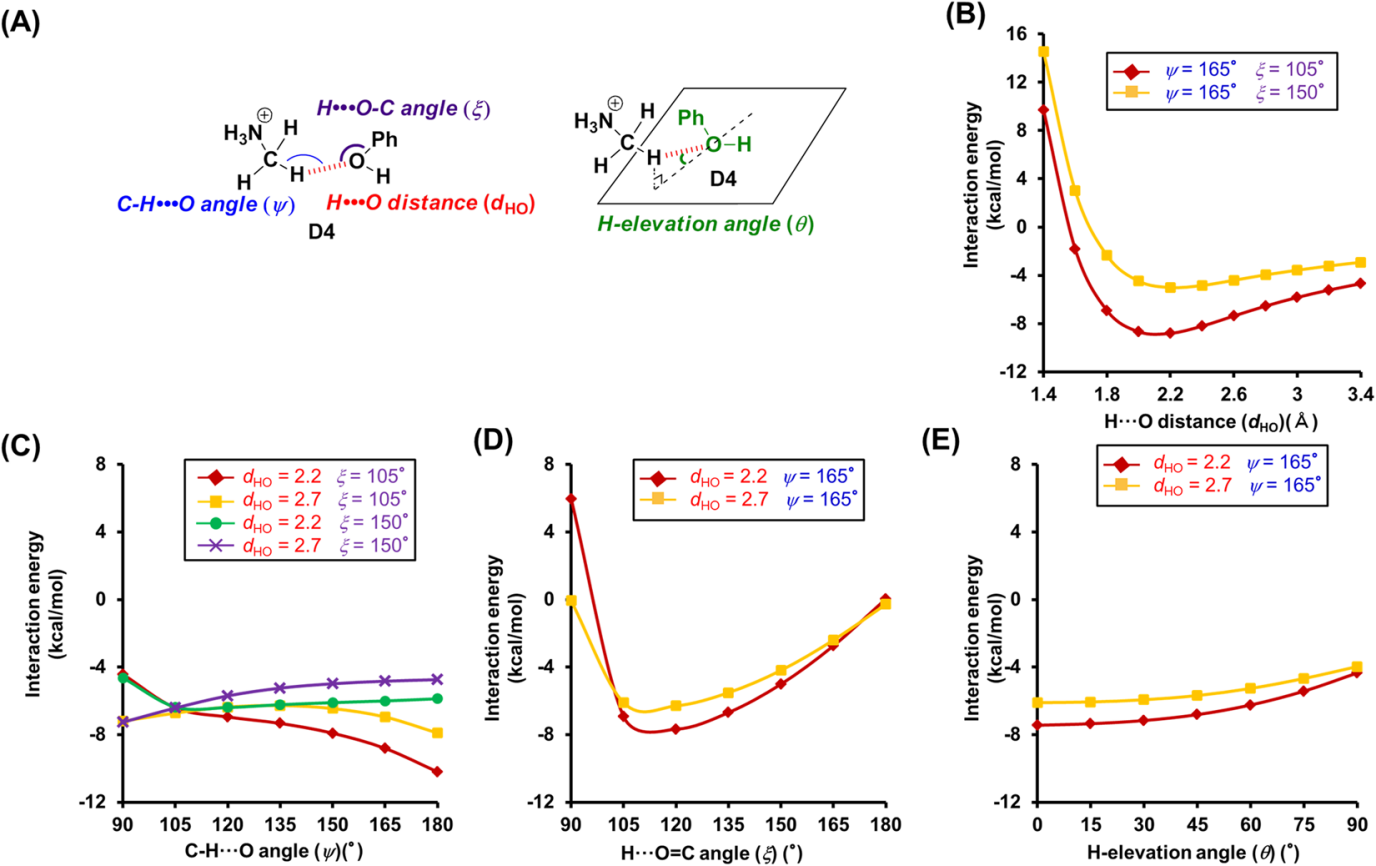
Theoretical analysis of the dependence of the interaction energies on the geometry of the N^+^-C-H···O hydrogen bonds between phenol (**4**) and monomethylamine (**5**). (**A**) Geometry of the N^+^-C-H···O hydrogen bond model between **4** and **5**. (**B**–**E**) Interaction energies of the N^+^-C-H···O hydrogen bond as a function of the following geometry parameters. (**B**) H···O distance (*d*_HO_). (**C**) C-H···O angle (*ψ*). (**D**) H···O=C angle (*ξ*). (**E**) H-elevation angle (*θ*). For the analysis of the H···O distance, the C-H···O, H···O=C, and H-elevation angles were kept constant (0°). Interaction energies were corrected for BSSE by counterpoise correction.

In order to establish selection criteria for N^+^-C-H···O hydrogen bonds, we used **A4**–**D4**, which consist of **1**–**4** and **5** as a simple model for N^+^-C-H···O hydrogen bonds, and examined the dependence of their interaction energy on the geometry. Specifically, we measured the H···O distances (*d*_HO_), the C-H···O angle (*ψ*), the H···O=C/H···O-C angle (*ξ*), and the H-elevation angle (*θ*)^30^ (Figs 2A, 3A, 4A and 5A). Initially, we examined the dependence of the interaction energy on the H···O distance in **A4**, whose C-H···O, H···O=C, and H-elevation angles were kept constant (Fig. 2B). For H···O distances in the range of 2.0–2.7 Å in **A4**, low interaction energies (−16.19 to −7.3 kcal/mol) were calculated (Fig. 2B), which is similar to the case of O-H···O hydrogen bonds (Supplementary Fig. S8F). The estimation of the distance dependence in **B4**–**D4** was carried out in a similar fashion. As **B4** is basically formed by ionic interactions between an anion and a cation, its interaction energy was significantly lower in the H···O distance region of 1.4–2.7 Å (Fig. 3B). The distance dependence of **C4** and **D4** was similar to that of **A4** (low energy range of **C4**: 1.8–2.7 Å, **D4**: 2.0–2.7 Å) (Figs 4B and 5B). In general, H···O distances <2.7 Å are regarded as hydrogen bonds, and (C-)H···O distances in the crystals of small molecules are frequently <2.4 Å^30^. This is also reflected in our calculations, which indicate that interaction energies are low for H···O distances <2.7 Å, while strong N^+^-C-H···O hydrogen bonds were estimated for H···O distances <2.4 Å (Supplementary Fig. S9).

Subsequently, we tested the dependence of the interaction energy of **A4** on the C-H···O angle (*ψ*). For 90° < *ψ* < 180°, the interaction energy is relatively low (Fig. 2C), which is similar to the case of the other hydrogen acceptors (**B4**: 90° < *ψ* < 180°, **C4** and **D4**: 105° < *ψ* < 180°) (Figs 3C, 4C and 5C). However, the trends for the changes of the interaction energy in N^+^-C-H···O hydrogen bonds are different to that in O-H···O hydrogen bonds. While for O-H···O angles ~180°, the interaction energy of O-H···O hydrogen bonds decreases (Fig. S8G), a similar trend could not be established for N^+^-C-H···O hydrogen bonds, although some cases involving **3** and **4** revealed a preference for *ψ* ≈ 180° (Figs 4C and 5C). The results of these calculations can be rationalized by examining the electrostatic potential maps of the hydrogen donors. The electrostatic potential map of **5** suggests that the positive charges for the H atoms of the N^+^-C-H groups are widely distributed (Supplementary Fig. S2A), while the positive charge of the hydroxyl hydrogen atom of e.g. ethanol is limited to its O-H line (Supplementary Fig. S2F). Therefore, the *ψ* angles of N^+^-C-H···O hydrogen bonds may vary in a wide range, whereas the O-H···O angles of O-H···O hydrogen bonds are required to be ~180°. Additionally, the O-H···O hydrogen bonds can be weakened by exchange repulsion between the O-H group and H···O hydrogen bond when the O-H···O angle is ~90°, while the exchange repulsion in N^+^-C-H···O hydrogen bonds should be weaker in comparison.

Then, we tested the dependence of the interaction energies on the H···O=C/H···O-C angles (*ξ*). Calculating the interaction energies, we discovered that these energies depend on the type of hydrogen acceptors. In the case of amide acceptors, i.e., for 105° < *ξ* < 180°, the interaction energies are low and the preferred angles are *ξ* > 135° (Fig. 2D and Supplementary Fig. S9A). Conversely, heteroatom-hydrogen bonds between ethanol and *N*-methylacetamide showed preferences for 105° < *ξ* < 120° (Supplementary Fig. S8H). In the case of carboxylate acceptors, a wide angle range (90° < *ξ* < 270°) was permissible, and **B4** exhibited a preference for *ξ* > 240° (Fig. 3D). This should be attributed to the fact that the H atom can interact with two O atoms of the carboxylate moiety. In the case of alcohol acceptors, the *ξ* angles strongly affect the interaction energies (permissible angle range: 105° < *ξ* < 150°, preferential angle range: *ξ* = 105°–120°) (Figs 4D and 5D and Supplementary Fig. S9C,D). For carboxylate and alcohol acceptors, the properties of the *ξ* angles of the N^+^-C-H···O hydrogen bonds are similar to those of the O-H···O hydrogen bonds (Supplementary Fig. S8H).

Furthermore, we investigated the H-elevation angles (*θ*). For *θ* < 70° (**A4**), *θ* < 60° (**C4**), and *θ* < 75° (**D4**), low interaction energies were calculated (Figs 2E, 4E and 5E). Conversely, *θ* has no effect on the interaction energy in **B4** (Fig. 3E).

The *ξ* and *θ* angles of the N^+^-C-H···O hydrogen bonds are also affected by the negative charge on the O atom of the hydrogen acceptor. The negative charges in *N*-methylacetamide (**1**) and propanoate (**2**) are widely distributed (Supplementary Fig. S2D,E), which leads to a concomitant wide range of the *ξ* and *θ* angles (Figs 2D,E and 3D,E). The most negatively charged part of **1** coincides with the C=O line and the plane of the carbonyl group (Supplementary Fig. S2D), which is consistent with the estimated preferences of *ξ* > 135°and *θ* = ~0° (Fig. 2D,E and Supplementary Fig. S9A). The negative charge of the O atom in ethanol (**3**) and phenol (**4**) is centered on the area where the plane bisecting the C-O-H angle intersects with the C-O-H plane (Supplementary Fig. S2F,G). Because the H atom in the N^+^-C-H···O hydrogen bonds with alcohol acceptors shows positional preferences in proximity to the planes, *ξ* = 105°–120° and *θ* = ~0° were estimated (Figs 4D,E and S5D,E and Supplementary Fig. S9).

Based on the results of these quantum chemical calculations, we were able to establish, for the first time, selection criteria for N^+^-C-H···O hydrogen bonds (Table 1).

### PDB survey

**Figure 6:**
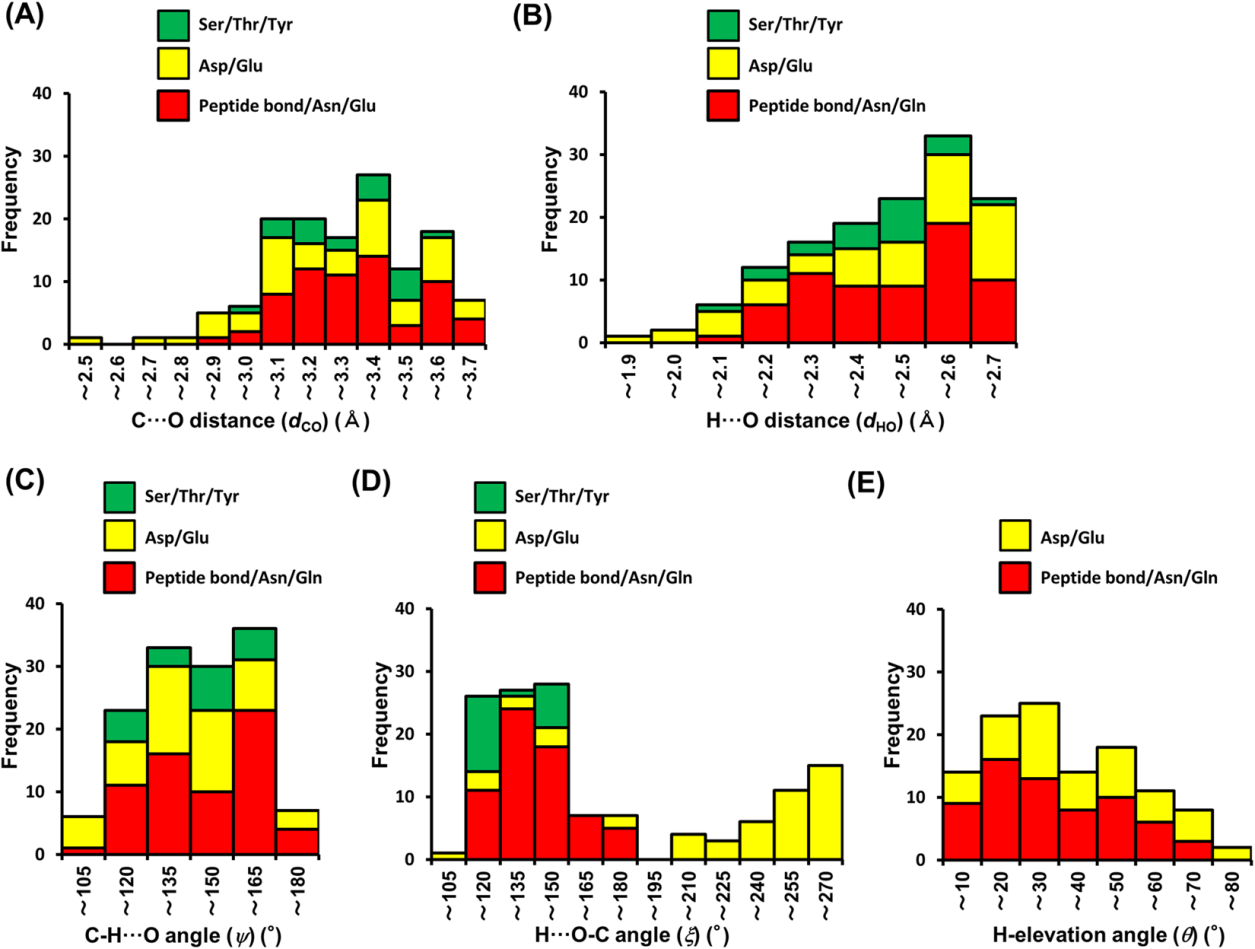
Histogram for the geometry of the N^+^-C-H···O hydrogen bonds. (**A**) C···O distance (*d*_CO_). (**B**) H···O distance (*d*_HO_). (**C**) C-H···O angle (*ψ*). (**D**) H···O-C angle (*ξ*). (**E**) H-elevation angle (*θ*).

To verify the existence of N^+^-C-H···O hydrogen bonds in protein-ligand complexes, we examined protein-ligand interactions in X-ray structures registered on the PDB. In this survey, we analyzed structures with a resolution of ≤2.80 Å. The positions of hydrogen atoms were determined using the drug discovery studio 3.5 software, as information on hydrogen positions in X-ray structures is generally not included. Therefore, the survey was conducted not only in accordance with the selection criteria established in the previous section (Table 1), but also under consideration of the following two points: (i) as the positions of the H atoms in protein structures cannot be determined accurately, we also used a carbon criteria set (C···O distance, N-C···O, and C···O=C/C···O-C angles, as well as C-elevation angles; Supplementary Table S2), which is based on the criteria shown in Table 1; (ii) H- and C-elevation angles of the interaction with the O atoms of Ser/Thr/Tyr were not surveyed, due to the difficulties associated with the determination of the position of the H atoms of the hydroxyl groups. In this study, we analyzed 159 X-ray crystal structures of complexes with ligands that contain aliphatic amines. This survey of 373 carbon atoms that are covalently bound to an aliphatic amino group in 159 ligands allowed us to identify 135 N^+^-C-H···O hydrogen bond interactions in 86 structures (Fig. 6, Supplementary Fig. S10, and Supplementary Tables S3–S6) and representative examples are shown in Supplementary Figs S11–S17.

The distribution of the C···O (*d*_CO_) and H···O (*d*_HO_) bond distances (Fig. 6A,B) shows that ~40% of the 135 interactions include distances of <3.2 Å and <2.4 Å, respectively. According to our calculations (Figs 2B, 3B, 4B, 5B and Table 1), and considering that strong hydrogen bonds (X-H···Y) exhibit bond lengths that are shorter than the sum of van der Waals radii of X and Y (sum of van der Waals radii for C and O: 3.22 Å)^31^, these N^+^-C-H···O hydrogen bonds can hence be classified as strong hydrogen bonds.

In 90% of the examined cases, we observed 105° < *ψ* < 165° (Fig. 6C), which is consistent with our calculations. These results suggest that the strength of the N^+^-C-H···O hydrogen bond does not depend on *ψ* (Figs 2C, 3C, 4C and 5C).

Subsequently, we examined the *ξ* angles (Fig. 6D). In peptide bond/Asn/Gln acceptors, ~45% of the cases, i.e., 30 out of the 65 peptide bond/Asn/Gln acceptors, exhibit *ξ* > 135°. In Asp/Glu acceptors, 26 out of the 50 cases exhibit *ξ* > 240°. Although we did not encounter many cases involving alcohol acceptors (20 cases) in the *ξ* dataset, we observed 13 interactions with 105° < *ξ* < 135°. According to our calculations (Figs 2D, 3D, 4D and 5D), these three types of contacts should be classified as strong N^+^-C-H···O hydrogen bonds.

Finally, we focused on the elevation angles of amide and carboxylate acceptors. The H-elevation (*θ*) and C-elevation (*ϕ*) angles are subject to a certain degree of correlation (Supplementary Fig. S18A). Among 65 cases of amide acceptors, we found 36 contacts with small elevation angles (*θ* < 30°, *ϕ* < 30°), whereas contacts of carboxylate acceptors exhibited a wide distribution of these angles (Fig. 6E and Supplementary Fig. S18A). The results of this survey are consistent with those of our calculations (Figs 2E and 3E). It should be especially noteworthy that we found 12 contacts among the Asp/Glu acceptors with *θ* < 30° and *ξ* > 240° (Supplementary Fig. S18B). According to our calculations, these contacts should be classified as especially strong N^+^-C-H···O hydrogen bonds, as the H atom can interact with two O atoms of the carboxylate group (Fig. 3D and Supplementary Fig. S17). In summary, the PDB survey allowed us to identify numerous examples of N^+^-C-H···O hydrogen bonds formed between proteins and their ligands. In combination with our energy calculations, these results suggest that N^+^-C-H···O hydrogen bonds should contribute significantly to the formation of protein-ligand complexes and to the activity of the ligand.

### Experimental investigation

**Figure 7:**
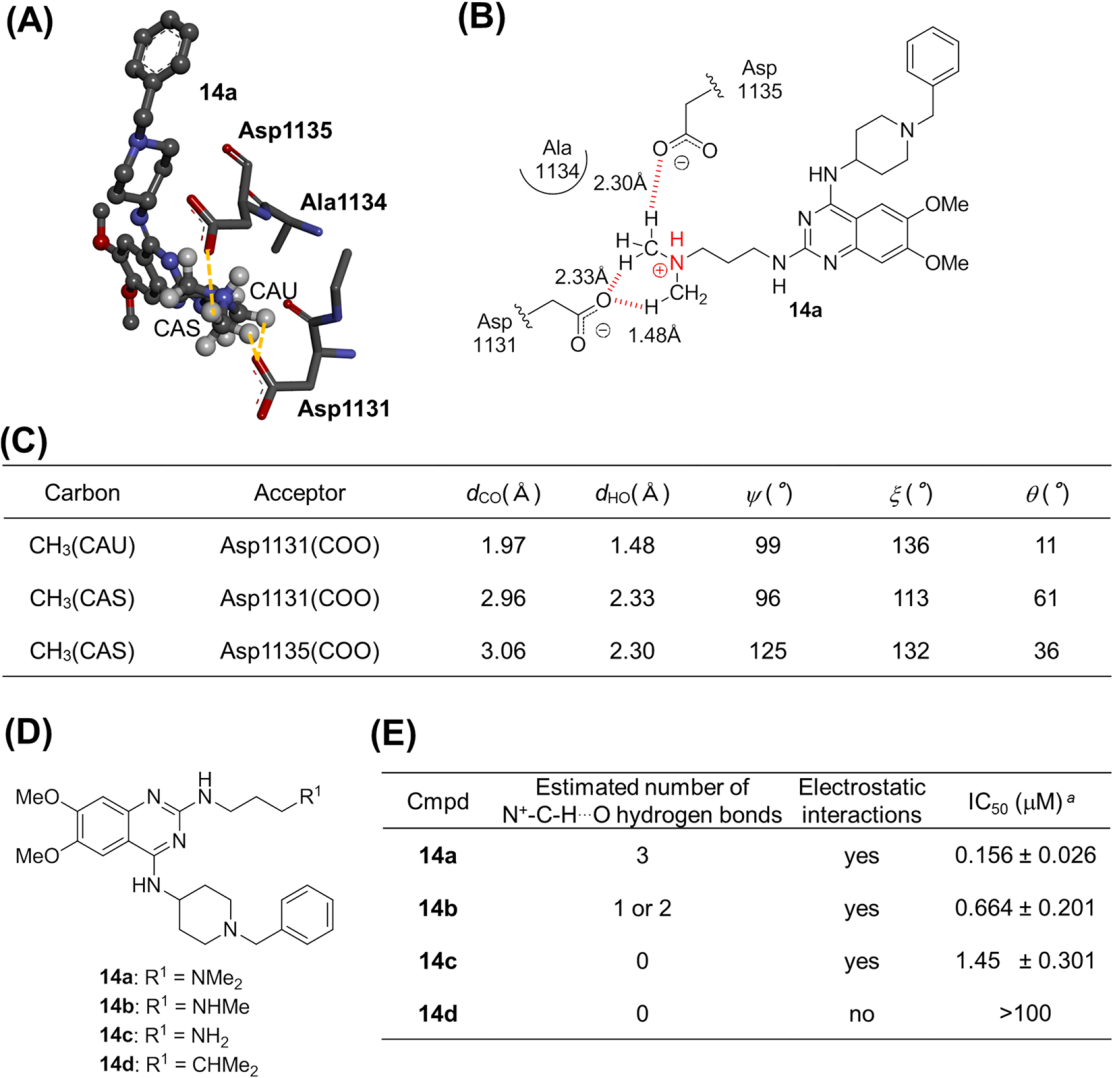
Experimental investigation of the N^+^-CH···O hydrogen bonds focusing on the G9a-like protein (GLP)-inhibitor complex. (**A**) X-ray structure of GLP complexed with **14a** (PDB ID: 3MO0). This figure shows **14a** (ball-and-stick) and amino acid residues (stick) within a 5 Å radius from the nitrogen atom of **14a**. (**B**) Schematic diagram of the binding mode between **14a** and GLP. The red dotted lines indicate C-H···O hydrogen bonds. (**C**) Geometry profile for the N^+^-C-H···O contacts between GLP and **14a**. The X-ray structure of GLP complexed with inhibitor **14a** suggests that the C-H groups adjacent to the nitrogen atom in the dimethylamino group of **14a** engage in three C-H···O hydrogen bonds with the Asp1131 and Asp1135 residues of GLP. The hydrogen atom of the ammonium cation does not form any hetero hydrogen bonds. (**D**) Chemical structures of **14a**–**d**. (**E**) *In vitro* GLP-inhibitory activity of **14a**–**d**. ^*a*^Values represent mean values of at least three experiments.

To experimentally validate the importance of the N^+^-C-H···O hydrogen bonds in protein-ligand complexes, we investigated if the N^+^-C-H···O hydrogen bonds between proteins and ligands affect the activity of the ligands. Among the X-ray crystal structures in the aforementioned PDB survey, we focused on the G9a-like protein (GLP)-inhibitor complex **14a** (Fig. 7A), one of histone methylransferases^32^. Its X-ray crystal structure suggests that the C-H groups adjacent to the nitrogen atom in the dimethylamino group of **14a** engages in three C-H···O hydrogen bonds with two Asp residues of GLP (Fig. 7A–C). In order to examine the potential importance of N^+^-C-H···O hydrogen bonds for the formation of the protein-ligand complex and its effect on the GLP-inhibitory activity of **14a**, we designed and synthesized monomethylamine **14b** and amine **14c**, both of which lack C-H groups adjacent to the nitrogen atom, as well as alkyl compound **14d**, in which the nitrogen atom of **14a** is replaced by a carbon atom (Fig. 7D). Compounds **14b**–**d** were expected to exhibit two or one, no, and no C-H···O hydrogen bonds, respectively (Fig. 7E). If the C-H···O hydrogen bonds of **14a** are responsible for the GLP-inhibitory activity, the activity of compounds **14b**–**d** should be weaker than **14a**. Accordingly, **14b**–**d** were also examined with respect to their GLP-inhibitory activity (Fig. 7E and Supplementary Fig. S19). The parent compound **14a** exhibits a dose-dependent inhibitory activity of GLP (IC_50_ = 0.156 μM). Relative to **14a**, **14b** significantly reduced the GLP-inhibitory activity (IC_50_ = 0.664 μM) in the 0.1–1 μM concentration range. In the same concentration range, **14c** showed a decreased GLP-inhibitory activity (IC_50_ = 1.45 μM) compared to **14a** and **14b**, while **14d** did not show any GLP inhibition up to 3 μM. The latter result should most likely be rationalized in terms of a lack in both electrostatic interactions and C-H···O hydrogen bonds between the ligand and GLP. Moreover, we determined the dissociation constant and thermodynamic parameters of **14a**–**c** by means of isothermal titration calorimetry (ITC). As shown in Supplementary Fig. S20, the dissociation constant of **14a**–**c** was distinctly dependent on the number of methyl group (*K*_D_: 0.171 μM for dimethyl **14a**, 0.617 μM for monomethyl **14b**, 0.944 μM for non-methyl **14c**), which is consistent with the IC_50_ values of **14a**–**c**. The thermodynamic parameters were also dependent on the number of methyl group: the ΔH values for dimethyl **14a**, monomethyl **14b**, and non-methyl **14c** were −10.7 kcal/mol, −8.58 kcal/mol, and −7.04 kcal/mol, respectively; the −TΔS values for **14a**, **14b**, and **14c** were 1.42 kcal/mol, 0.10 kcal/mol, and −1.19 kcal/mol, respectively (Supplementary Fig. S20). The ITC data revealed that the removal of the methyl group resulted in both an unfavorable enthalpy change and a favorable entropy change. However, the change in enthalpy by the removal of the methyl group was larger than that in entropy, which led to the decreased binding affinity (increased *K*_D_ and ΔG). It is well known that enthalpic forces such as hydrogen bond formation can decrease entropic forces by restricting the degrees of freedom of water molecules and protein conformation^33^. Based on this, there is a possibility that the methyl group of **14a** and **14b** reduced the ΔH value by the conformational fixation of GLP through the formation of N^+^-C-H···O hydrogen bonds, which resulted in reduction of the TΔS value by decreasing the degrees of freedom of water molecules and protein conformation, and the effect of enthalpy was larger than that of entropy. Taken together, the observed GLP-inhibitory activity, disassociation constant and thermodynamic parameters for **14a**–**d** strongly suggests that the N^+^-C-H···O hydrogen bonds in the GLP-ligand complex are responsible for their GLP-inhibitory activity, although the effect might not be attributed exclusively to the N^+^-C-H···O hydrogen bonds.

**Figure 8:**
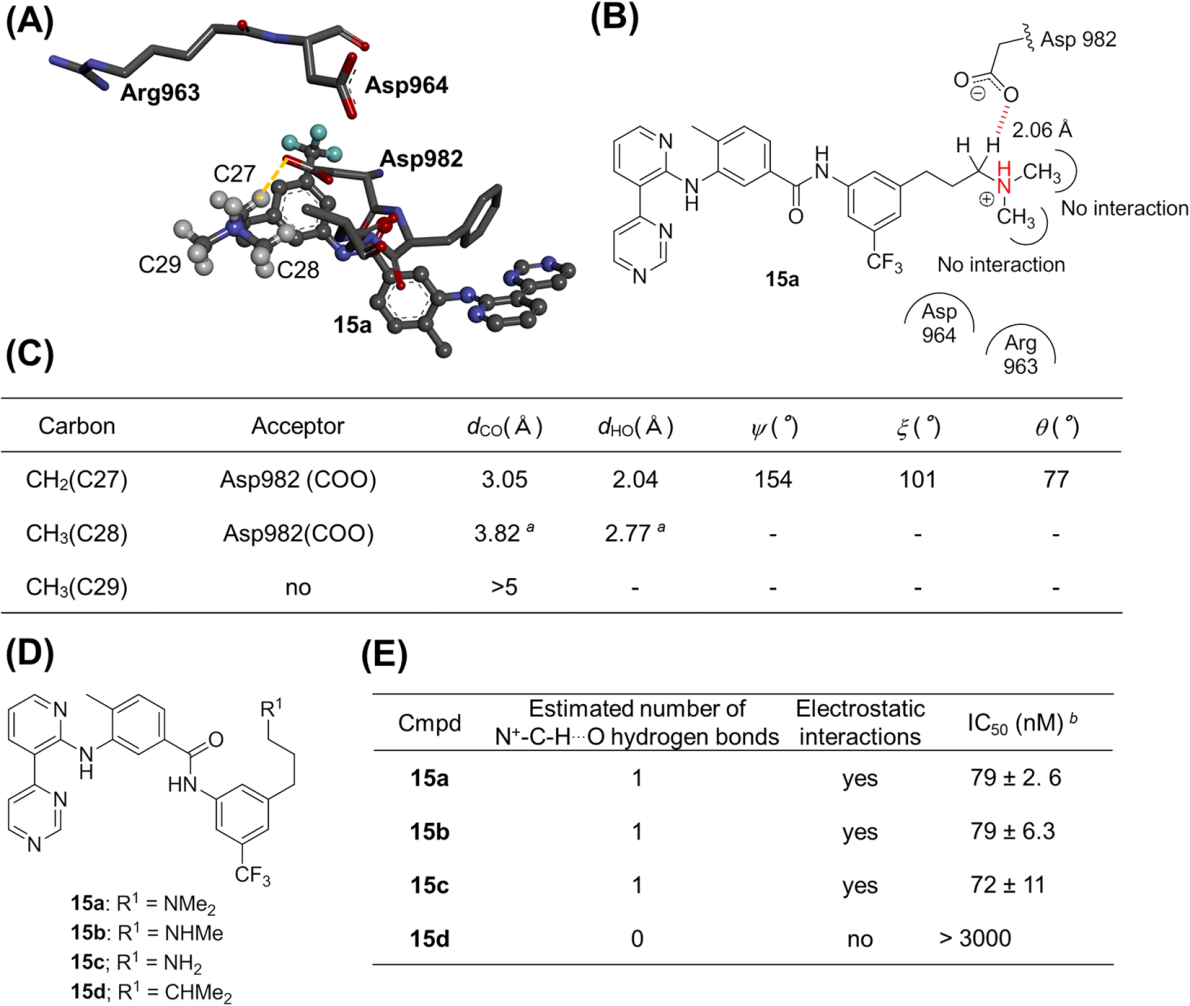
Experimental investigation of N^+^-CH···O hydrogen bonds focusing on tyrosine kinase with Ig and EGF homology domains-2 (tie-2). (**A**) X-ray structure of tie-2 kinase complexed with inhibitor **15a** (PDB ID: 2OO8). This figure shows **15a** (ball-and-stick) and amino acid residues (stick) within a 5 Å radius from the nitrogen atom of **15a**. (**B**) Schematic diagram of the binding mode of **15a** to tie-2. The red dotted line indicates a C-H···O hydrogen bond. (**C**) The geometry profile of N^+^-C-H···O contacts between tie-2 and **15a**. The methylene group that is in conjugation with the aliphatic amino group forms an N^+^-C-H···O hydrogen bond, but the methyl groups do not. The hydrogen atom of the ammonium cation does not form any hetero hydrogen bonds. (**D**) Chemical structures of **15a**–**d**. (**E**) Tie-2-inhibitory activity of **15a**–**d**. ^*a*^Out of criteria for N^+^-C-H···O hydrogen bonds. ^*b*^Values represent mean values (±SD) of at least three experiments.

In addition, we investigated a ligand, whose N^+^-CH_3_ groups do not engage in N^+^-C-H···O hydrogen bonds with any protein-based oxygen atom. The X-ray crystal structure of tyrosine kinase with Ig and EGF homology domains-2 (tie-2) complexed to its inhibitor **15a** indicates that the methylene group that is in conjugation with the aliphatic amino group forms an N^+^-C-H···O hydrogen bond, but the methyl groups do not (Fig. 8A–C)^34^. We furthermore prepared monomethylamine **15b**, amine **15c**, and alkyl compound **15d** (Fig. 8D), and evaluated their inhibitory activity. As expected, we did not observe any significant difference among the IC_50_ values of **15a**–**c** (Fig. 8E), although the activity of **15d** was lower than that of **15a**–**c**. In their entirety, the experimental results (Figs 7 and 8) highlight the importance of the N^+^-C-H···O hydrogen bonds in protein-ligand complexes and for the activity of the ligands.

## Conclusions

Herein, we have demonstrated for the first time the significance of N^+^-C-H···O hydrogen bonds in protein-ligand complexes by establishing selection criteria based on quantum chemical calculations, a PDB survey, and experimental investigations. Our calculations revealed that hydrogen bonds can be formed between the H atom of N^+^-C-H groups and the oxygen atoms of amides, carboxylates, or alcohols. The low interaction energies of these bonds are comparable to heteroatom-hydrogen bonds, π/π interactions, cation/π interactions, or CH/π interactions, all of which are routinely considered in drug design. Based on the geometric analysis of the quantum chemical calculations, we theoretically established selection criteria for the N^+^-C-H···O hydrogen bonds. A PDB survey of X-ray structures based on the thus obtained criteria revealed that numerous proteins-ligand complexes contain such N^+^-C-H···O hydrogen bonds. Thus, we used a simple method to qualitatively and comprehensively estimate N^+^-C-H···O hydrogen bonds based on a minimum of information required, although more detailed data such as environmental interactions should be helpful for further understanding of N^+^-C-H···O hydrogen bonds. Finally, we experimentally corroborated our hypothesis that the presence and magnitude of the N^+^-C-H···O hydrogen bonds strongly affects the activity of the proteins-ligand complexes. The results of this study should thus help to further the understanding of ligand recognition by proteins, which should be beneficial for the drug design.

## Methods

### Quantum chemical calculations

All calculations were performed using the Gaussian 09 package^35^. Structural optimizations and single-point calculations were carried out using the M06-2X variant of density functional theory (DFT)^36^, which is often used for long distance interactions, with the 6-311++G** basis set in the gas phase or in the water phase^37^. Structures were considered as minima in case of all harmonic frequencies being positive. All interaction energies were corrected for basis set superposition errors (BSSE) by the counterpoise procedure^38^. To gauge the accuracy of the M06-2X/6-311++G** level of theory, the thus obtained binding energies were compared to those obtained from MP2/aug-cc-pVTZ (Supplementary Table S7). Natural bond orbital (NBO)^39,40^ analyses were performed via the procedures contained within Gaussian 09. Electrostatic potential surfaces were created using the GaussView 5.0 software package. The electrostatic potential for each structure was mapped onto a total electron density surface. The borders for the criteria were determined based on the energy/angle slope and the lowest energy; the borders were set at the point where the slope is >1.5% of the lowest energy of each curve, or where the energy is <60% of the lowest energy of each curve.

### PDB survey

All X-ray structures of proteins bound to amine-containing ligands were downloaded from the Protein Data Bank (PDB). To gain a better understanding of N^+^-C-H···O interactions in protein-ligand complexes, we analyzed structures that were refined at a resolution of 2.80 Å. Duplicate structures and water molecules were omitted. The positions of hydrogen atoms were determined using the hydrogen position function of the drug discovery studio 3.5 software package. We confirmed the accuracy of the hydrogen positions by comparison with the positions obtained from the Molegro Virtual Docker 6.0 software package. N^+^-C-H···O hydrogen bond criteria were established based on the results of the quantum chemical calculations: (i) C···O distance (*d*_CO_): <3.7 Å^27^; (ii) H···O distance (*d*_HO_): <2.7 Å; (iii) C-H···O angle (*ψ*): >90° for the peptide bond/Asn/Gln and Asp/Glu acceptors, or >105° for the Ser/Thr/Tyr acceptors; (iv) H···O=C angle (*ξ*): >105° for the peptide bond/Asn/Gln acceptors, >90° for the Asp/Glu acceptors, or 105°–150° for the Ser/Thr/Tyr acceptors; (v) H···elevation angle (*θ*): <70° for the peptide bond/Asn/Gln acceptors, <80° for the Asp/Glu acceptors; (vi) N^+^-C···O angle (*α*): 60°–160°; (vii) C···O-C angle (*β*): >95° for the peptide bond/Asn/Gln, >90° for the Asp/Glu acceptors, or 95°–160° for the Ser/Thr/Tyr acceptors; (viii) C-elevation (*ϕ*): <80° for the peptide bond/Asn/Gln and <90° for Asp/Glu. The H-elevation angle (*θ*) was calculated according to sin*θ* = sin*ξ* · sin*χ*, whereby *χ* refers to the average of the two corresponding dihedral angles H···O=C-O(N) and H···O=C-C. Similarly, the C-elevation angle (*ϕ*) was calculated by sin*ϕ* = sin*β* · sin*δ*, whereby *δ* refers to the average of the two corresponding dihedral angles C···O=C-O(N) and C···O=C-C.

### GLP activity assay

The GLP activity assay was carried out using a GLP Chemiluminescent Assay Kit (Catalog #53007, BPS Bioscience, Inc.). Microwells were rehydrated by adding 200 μL of tris buffered saline with Tween 20 (TBS-T: 1x TBS, pH = 8.0, containing 0.05% Tween-20) to every well, followed by incubation at room temperature for 45 minutes. After removing TBS-T, the inhibitors were incubated in the presence of 2 μM SAM and 40 ng of GLP in the supplied buffer on the microwells of (120 min, room temperature, total volume: 50 μL). After the enzymatic reaction, every well was washed three times with TBS-T (100 μL) and blocked for 10 min with blocking buffer. Then, 100 μL of primary anti-body solution (1:400 dilution) were added to the microwells, followed by incubation (2 h). The wells were probed with the primary antibody, washed three times with TBS-T (100 μL), incubated (2 h, room temperature) with sheep secondary anti-body (1:1000 diluted), and again washed three times with TBS-T (100 μL). The chemiluminescence of the wells, to which detection reagents were added, was measured on a chemiluminescence reader (ARVO X3 Multilabel Plate Reader), and the values of % inhibition were calculated from the chemiluminescence readings of inhibited wells relative to those of control wells.

### ITC experiment

A GLP recombinant protein solution (Catalog #31920, Active Motif, Inc.) was concentrated by ultrafiltration and the buffer was replaced with test buffer (20 mM Tris pH 8.0, 150 mM NaCl, 2% DMSO and 50 μM AdoMet). ITC measurements of GLP inhibitors binding to the GLP were recorded at 25 °C using a MicroCal PEAQ-ITC microcalorimeter (Malvern Panalytical) in the test buffer. The titrations were performed using 10 μM GLP solution in the sample cell and 100 μM inhibitor solution in the syringe. A typical experiment consisted of 19 injections under the following parameters: one injection of 0.4 μL followed by 18 injections of 2.0 μL; 150 s spacing between injections; 750 rpm stirring speed; and reference power set to 5 μcal s^−1^. The heat of dilution of the ligand into the buffer measured independently was essentially similar to the heat signal obtained at the end of the titration; so the signal of the last injection was used as the background heat signal. The experimental data were analyzed using the MicroCal PEAQ-ITC Analysis software (Malvern Panalytical), and fitted using the one-site binding model. Values of *ΔG*, *K*_D_, and −T*ΔS* were calculated using the thermodynamic relationships, *ΔG* = −RT ln*K*_a_, *K*_D_ = 1/*K*_a_, and *ΔG* = *ΔH* − *TΔ*S.

### Tie-2 activity assay

The tie-2 activity assay was carried out using human recombinant active enzyme (Catalog #40370, BPS Bioscience, Inc.) and Universal Tyrosine Kinase Assay Kit (Catalog # MK410, TAKARA BIO, Inc.). The inhibitors (final concentration: 3, 0.3, 0.1, 0.07, or 0.03 μM) were incubated in the presence of 40 mM ATP and 20 ng of tie-2 in the supplied buffer including 5 mM DTT on the microwells of the supplier (45 min, 37 °C, total volume: 50 μL). After the enzymatic reaction, the mixtures were removed from the wells and all wells were washed four times with phosphate buffered saline with Tween 20 (PBS-T, 100 μL). Then, the wells were blocked (30 min) with blocking buffer, before 50 μL of anti-phosphated Tyrosine-POD were added to the wells, and incubation was continued (30 min, 37 °C). The wells were probed, washed four times with PBS-T (100 μL), and subsequently incubated with HRP substrate solution (15 min, 37 °C). Finally, the reaction was stopped by addition of aqueous H_2_SO_4_ (1N). The absorbance at 450 nm of the wells, to which detection reagents were added, was measured in a chemiluminescence reader (ARVO X3 Multilabel Plate Reader), and the values of % inhibition were calculated from the absorbance readings of inhibited wells relative to those of control wells.

## Supplementary information

Supplementary Information
