# Supplementary material for: N+-C-H···O Hydrogen bonds in protein-ligand complexes
Source: Sci Rep. 2019 Jan 25;9:767. doi: 10.1038/s41598-018-36987-9 (PMC6347603; doi:10.1038/s41598-018-36987-9)
Supplement: Supplementary file 1 — Supplementary Information [file 41598_2018_36987_MOESM1_ESM.docx]

Supplementary Information for

**N^+^-C-H···O Hydrogen bonds in protein-ligand complexes**

Yukihiro Itoh^1^,Yusuke Nakashima^1^,Shuichiro Tsukamoto^2^, Takashi Kurohara^1^, Miki Suzuki^1^, Yoshitake Sakae^2^, Masayuki Oda^3^, Yuko Okamoto^2,4^, & Takayoshi Suzuki,^1,4,^*

^1^Graduate School of Medical Science, Kyoto Prefectural University of Medicine, 1-5 Shimogamohangi-cho, Sakyo-ku, Kyoto 606-0823, Japan

^2^Department of Physics, Nagoya University, Furo-cho, Chikusa-ku, Nagoya, Aichi 464-8602, Japan

^3^Graduate School of Life and Environmental Sciences, Kyoto Prefectural University, Kyoto. , 1-5 Shimogamohangi-cho, Sakyo-ku, Kyoto, 606-8522, Japan.

**^4^**CREST, Japan Science and Technology Agency (JST), 4-1-8 Honcho Kawaguchi, Saitama 332-0012,

*Corresponding author. Email: suzukit@koto.kpu-m.ac.jp

Table of Contents S2

SI Tables S3

Table S1. Summary of the C-H···O hydrogen bond profiling of complexes **A**−**D**. S3

Table S2. Summary of the geometry profiling for N^+^-C-H···O hydrogen bonds with respect to the

carbon atom S4

Table S3. PDB survey list. S5

Table S4. Results of the PDB survey on peptide bond/Asn/Gln hydrogen acceptors. S6

Table S5. Results of the PDB survey on Asp/Glu hydrogen acceptors. S8

Table S6. Results of the PDB survey on Ser/Thr/Tyr hydrogen acceptors. S10

Table S7. Interaction energies calculated at the M06-2X/6-311++G** or

MP2/aug-cc-pVTZ level of theory. S11

SI Figures S12

Figure S1. Examples for C-H···Y hydrogen bonds activated by a cationically charged atom and

working hypotheses. S12

Figure S2. Formal charges and/or electrostatic potential maps for **1**–**5**, **8** and **9**. S13

Figure S3. Optimized geometries for trimethylammonium (**7**) complexed with phenol (**4**) S14

Figure S4. Theoretically optimized geometries and counterpoise-corrected interaction energies for

N^+^-C-H··O hydrogen bond models (calculated in water). S15

Figure S5. Optimized geometries and interaction energies for heteroatom-hydrogen bond,

/ interaction, cation/ interaction, or CH/ interaction models. S16

Figure S6. Theoretically optimized geometries and counterpoise-corrected interaction energies

of **10**–**13** complexed with **1**–**4**. S17

Figure S7. Theoretically optimized geometries and counterpoise-corrected interaction energies for

N^+^-C-H··O hydrogen bond models (ternary complex). S18

Figure S8. Theoretical analysis of the dependence of the interaction energies on the

geometry of the O-H···O hydrogen bonds between **1**–**4** and ethanol (**3**). S19

Figure S9. Interaction models and their preferred geometries of complexes **A4**–**D4**. S20

Figure S10. Histogram for the geometry of N^+^-C-H···O hydrogen bonds with respect to

N^+^-C···O angle, C···O-C angle, and C-elevation angle. S21

Figure S11–S17. Representative examples of N^+^-C-H···O contacts found in the PDB survey. S22

Figure S18. Correlation between the H-elevation and C-elevation angles, or the H-elevation and

H···O-C angles. S29

Figure S19. GLP-inhibitory activity of **14a**–**14d**. S30

Figure S20. Determination of disassociation constants and thermodynamic parameters of **14a**–**14c**

by isothermal titration calorimetry (ITC). S31

Synthesis of compounds 14 and 15. S32

General synthetic methods. S32

Scheme S1. Reaction scheme for the synthesis of **14a**−**d**. S33

Scheme S2. Reaction scheme for the synthesis of **15a**−**d**. S39

NMR spectra S49

**SI References** S57

**Table S1. Summary of the C-H···O hydrogen bond profiling of complexes A**−**D**.

| Complex | X | Calculation condition | C-H distance ( Å) | | | | | Interaction energy  (kcal/mol) | Figure |
| --- | --- | --- | --- | --- | --- | --- | --- | --- | --- |
|  |  |  | a | b | c | d | e |  |  |
| **A1** | NH^+^ | gas phase | 2.20 | 2.23 | 2.23 | - | - | -22.01 | 1 |
| **A1** | NH^+^ | water | 2.30 | 2.30 | 2.30 | - | - | -21.02 | S4 |
| **A1** | NH^+^···OH_2_ | gas phase | 2.23 | 2.25 | 2.25 | - | - | -19.21 | S7 |
| **A2** | N | gas phase | 2.64 | 2.61 | No | - | - | -3.05 | S6 |
| **A3** | CH | gas phase | 2.58 | 2.58 | No | - | - | -3.54 | S6 |
| **B1** | NH^+^ | gas phase | 2.07 | 2.17 | 2.20 | 2.41 | 2.39 | -106.20 | 1 |
| **B1** | NH^+^ | water | 2.18 | 2.45 | 2.40 | 2.50 | 2.56 | -102.28 | S4 |
| **B1** | NH^+^···OH_2_ | gas phase | 2.12 | 2.20 | 2.20 | 2.43 | 2.44 | -99.43 | S7 |
| **B2** | N | gas phase | 2.33 | 2.53 | 2.54 | 2.59 | 2.59 | -9.41 | S6 |
| **B3** | CH | gas phase | 2.57 | 2.62 | 2.56 | 2.66 | 2.29 | -8.68 | S6 |
| **C1** | NH^+^ | gas phase | 2.31 | 2.31 | 2.51 | - | - | -13.49 | 1 |
| **C1** | NH^+^ | water | 2.36 | 2.36 | 2.55 | - | - | -12.87 | S4 |
| **C1** | NH^+^···OH_2_ | gas phase | 2.35 | 2.58 | 2.31 | - | - | -11.94 | S7 |
| **C2** | N | gas phase | 2.63 | 2.51 | 2.97 | - | - | -1.99 | S6 |
| **C3** | CH | gas phase | 2.55 | 2.54 | 2.91 | - | - | -2.43 | S6 |
| **D1** | NH^+^ | gas phase | 2.45 | - | - | - | - | -15.46 | 1 |
| **D1** | NH^+^ | water | 2.66 | - | - | - | - | -11.66 | S4 |
| **D1** | NH^+^···OH_2_ | gas phase | 2.54 | - | - | - | - | -12.38 | S7 |
| **D2** | N | gas phase | 3.08 | - | - | - | - | -4.09 | S6 |
| **D3** | CH | gas phase | 2.82 | - | - | - | - | -4.25 | S6 |

**Table S2. Summary of the geometry profiling for N^+^-C-H···O hydrogen bonds with respect to the carbon atom.**

| Hydrogen acceptor | Geometry | Range |
| --- | --- | --- |
| Peptide bond/Asn/Gln | C···O distance (*d*_CO_) | 2.3 Å < *d*_CO_ < 3.7 Å |
|  | N^+^-C···O angle (*α*) | 60° < *α * < 160° |
|  | C···O=C angle (*β*) | 95° < *β * < 180° |
|  | C-elevation angle (*φ*) | *φ* < 80° |
| Asp/Glu | *d*_CO_ | 1.8 Å < *d*_CO_ < 3.7 Å |
|  | *α* | 60° < *α * < 160° |
|  | *β* | 90° < *β * < 180° |
|  | *φ* | * * < 90° |
| Ser/Thr | *d*_CO_ | 2.4 Å < *d*_CO_ < 3.7 Å |
|  | *α* | 60° < *α * < 160° |
|  | *β* | 95° < *β * < 160° |
|  | *φ* | * * < 75° |
| Tyr | *d*_CO_ | 2.5 Å < *d*_CO_ < 3.7 Å |
|  | *α* | 60° < *α * < 160° |
|  | *β* | 95° < *β * < 160° |
|  | *φ* | * * < 85° |

**Table S3. PDB survey list.**

| PDB code | Resolution | Number of carbon atoms under survey | PDB code | Resolution | Number of carbon atoms under survey | PDB code | Resolution | Number of carbon atoms under survey |
| --- | --- | --- | --- | --- | --- | --- | --- | --- |
| 1BO4 | 2.30 | 4 | 4CK3 | 1.79 | 1 | 4WRH | 1.60 | 1 |
| 1D6Q | 2.80 | 3 | 4CP9 | 1.65 | 4 | 4X6M | 2.40 | 1 |
| 1EL5 | 1.80 | 3 | 4CQE | 2.30 | 3 | 4X8E | 1.60 | 4 |
| 1H39 | 2.80 | 3 | 4G0V | 2.55 | 4 | 4XCZ | 1.50 | 1 |
| 1IYK | 2.30 | 1 | 4GOL | 2.57 | 4 | 4XHX | 2.10 | 2 |
| 1M0W | 1.80 | 1 | 4I71 | 1.28 | 3 | 4XNB | 1.95 | 1 |
| 1N22 | 2.40 | 3 | 4IKR | 1.78 | 3 | 4XT0 | 2.07 | 1 |
| 1OHR | 2.10 | 3 | 4ITO | 1.16 | 3 | 4XWA | 1.89 | 3 |
| 1TMN | 1.90 | 2 | 4IXV | 2.30 | 3 | 4Y73 | 2.14 | 1 |
| 1UDT | 2.30 | 3 | 4JBS | 2.79 | 1 | 4YDF | 2.80 | 2 |
| 1WRK | 2.15 | 6 | 4JE5 | 1.91 | 1 | 4YML | 1.75 | 3 |
| 1X70 | 2.10 | 1 | 4JRV | 2.80 | 3 | 4YND | 2.79 | 4 |
| 1XH0 | 2.00 | 2 | 4KMN | 1.52 | 4 | 4YVF | 2.70 | 2 |
| 1YDR | 2.20 | 2 | 4KQP | 2.10 | 4 | 4YW5 | 2.30 | 1 |
| 1ZGI | 2.20 | 2 | 4KQQ | 2.10 | 2 | 4ZEG | 2.33 | 3 |
| 2AGW | 1.45 | 1 | 4L02 | 2.75 | 3 | 4ZFL | 1.70 | 5 |
| 2DFP | 2.30 | 3 | 4LDE | 2.79 | 1 | 4ZOL | 2.50 | 3 |
| 2GPU | 1.70 | 3 | 4LM5 | 2.25 | 1 | 4ZUP | 1.33 | 1 |
| 2JFA | 2.55 | 3 | 4MAN | 2.07 | 5 | 5A81 | 2.03 | 2 |
| 2OO8 | 2.20 | 3 | 4MDD | 2.40 | 3 | 5A8E | 2.40 | 1 |
| 2Q8C | 2.05 | 4 | 4MLN | 2.10 | 1 | 5AI9 | 1.80 | 2 |
| 2QB4 | 1.90 | 2 | 4MN3 | 1.54 | 4 | 5AJW | 2.50 | 1 |
| 2QEO | 2.32 | 1 | 4MQ4 | 2.20 | 2 | 5AM6 | 1.96 | 3 |
| 2V61 | 1.70 | 2 | 4MQF | 2.22 | 1 | 5AYF | 2.01 | 3 |
| 2VIN | 1.90 | 1 | 4MRG | 1.69 | 2 | 5BR1 | 1.85 | 1 |
| 2VNW | 2.09 | 1 | 4MTB | 1.22 | 1 | 5BS3 | 2.65 | 1 |
| 2X92 | 2.11 | 2 | 4MWZ | 1.50 | 3 | 5C0K | 2.20 | 1 |
| 2XHR | 2.20 | 3 | 4NLD | 2.75 | 3 | 5C1M | 2.10 | 4 |
| 3APR | 1.80 | 4 | 4OBQ | 2.19 | 3 | 5CFT | 1.50 | 7 |
| 3APV | 2.15 | 3 | 4OGU | 2.10 | 1 | 5CI7 | 1.74 | 1 |
| 3BRN | 2.00 | 1 | 4OQ0 | 1.42 | 1 | 5CSX | 2.51 | 3 |
| 3BU1 | 1.40 | 1 | 4P4B | 1.93 | 1 | 5DB1 | 1.86 | 3 |
| 3D4S | 2.80 | 1 | 4PCR | 2.15 | 1 | 5DFP | 2.20 | 3 |
| 3FUI | 2.20 | 2 | 4PF7 | 2.33 | 1 | 5DM0 | 1.75 | 1 |
| 3GKZ | 1.90 | 2 | 4PI3 | 1.27 | 3 | 5DP5 | 2.03 | 1 |
| 3GWS | 2.20 | 1 | 4PNI | 1.85 | 3 | 5DQC | 2.47 | 2 |
| 3HCC | 2.30 | 1 | 4PY2 | 2.15 | 2 | 5DSG | 2.60 | 4 |
| 3KM4 | 1.90 | 2 | 4Q3S | 2.11 | 3 | 5EAM | 1.80 | 3 |
| 3MO0 | 2.78 | 6 | 4Q8O | 1.89 | 3 | 5EMJ | 2.27 | 3 |
| 3NN6 | 2.19 | 3 | 4QF9 | 2.28 | 1 | 5EUD | 2.24 | 2 |
| 3NWW | 2.09 | 3 | 4QMS | 1.88 | 3 | 5EYM | 2.70 | 3 |
| 3PAH | 2.00 | 2 | 4QNQ | 2.30 | 6 | 5FHM | 1.55 | 2 |
| 3WC7 | 1.90 | 1 | 4QQK | 1.88 | 1 | 5FTQ | 1.70 | 3 |
| 3WHS | 1.80 | 1 | 4R0I | 1.90 | 1 | 5H8E | 2.15 | 1 |
| 3WZ6 | 1.40 | 2 | 4RVX | 1.96 | 1 | 5HBG | 1.90 | 1 |
| 3ZDH | 2.19 | 3 | 4RYZ | 2.50 | 1 | 5HHW | 1.79 | 3 |
| 4ABZ | 1.89 | 4 | 4U5N | 2.31 | 3 | 5HI7 | 2.15 | 7 |
| 4BKY | 1.83 | 3 | 4U6R | 2.50 | 1 | 5HJ9 | 1.28 | 3 |
| 4BWA | 2.45 | 1 | 4UAC | 1.60 | 2 | 5HX8 | 2.20 | 1 |
| 4C2V | 1.49 | 3 | 4UIB | 1.94 | 1 | 5IC2 | 2.10 | 1 |
| 4C39 | 1.98 | 2 | 4UVU | 1.95 | 3 | 5JYZ | 1.80 | 2 |
| 4C7F | 2.00 | 3 | 4WH5 | 1.82 | 3 | 5L2T | 2.37 | 2 |
| 4CGO | 1.30 | 3 | 4WKN | 2.00 | 3 | 5LJ1 | 1.90 | 2 |

**Table S4. Results of the PDB survey on peptide bond/Asn/Gln hydrogen acceptors.**

| Entry | PDB code | Protein | Carbon | Acceptor | C···O distance  (*d*_CO_) | H···O distance (*d*_HO_) | C-H···O  angle (**) | H···O=C angle (**) | H-  elevation angle (*θ*) | N^+^-C···O angle (*α*) | C···O=C angle (*β*) | C-  elevation angle (*φ*) |
| --- | --- | --- | --- | --- | --- | --- | --- | --- | --- | --- | --- | --- |
| 1 | 1EL5 | Sarcosine oxidase | CH_3_(C4) | Gly344 | 3.06 | 2.49 | 112 | 121 | 59 | 69 | 135 | 44 |
| 2 | 1N22 | (+)-Bornyl diphosphate synthase | CH(C14) | Val452 | 3.59 | 2.52 | 168 | 119 | 33 | 108 | 115 | 35 |
| 3 | 1OHR | Aspartylprotease | CH_2_(C10) | Gly27 | 3.51 | 2.49 | 153 | 113 | 51 | 91 | 112 | 58 |
| 4 | 1OHR | Aspartylprotease | CH(C8) | Gly27 | 3.62 | 2.64 | 148 | 124 | 46 | 86 | 121 | 54 |
| 5 | 1WRK | Human cardiac troponin C | CH_2_(C16) | Ser84 | 3.23 | 2.67 | 112 | 176 | 2 | 101 | 162 | 15 |
| 6 | 1WRK | Human cardiac troponin C | CH_2_(C16) | Met81 | 3.24 | 2.64 | 114 | 123 | 36 | 62 | 125 | 49 |
| 7 | 2DFP | Acetylcholinesterase | CH_2_(C5) | Gln500 | 3.61 | 2.6 | 154 | 124 | 8 | 97 | 119 | 15 |
| 8 | 2GPU | Estrogen Related Receptor-gamma | CH_3_(C25) | Leu440 | 3.18 | 2.13 | 163 | 148 | 32 | 116 | 154 | 26 |
| 9 | 2GPU | Estrogen related receptor-gamma | CH_3_(C26) | Glu441 | 3.67 | 2.69 | 150 | 118 | 44 | 124 | 123 | 46 |
| 10 | 2Q8C | JMJD2A | CH_3_(CM2) | Gly170 | 3.21 | 2.2 | 153 | 118 | 19 | 102 | 127 | 17 |
| 11 | 2VNW | Protein kinase B | CH_2_(C9) | Glu170 | 2.92 | 2.54 | 99 | 159 | 9 | 72 | 163 | 12 |
| 12 | 2X92 | Angiotensin converting enzyme | CH(CAX) | Ala138 | 3.1 | 2.32 | 127 | 141 | 20 | 71 | 155 | 19 |
| 13 | 2XHR | Heat shock protein HSP 90-alpha | CH_2_(C21) | Gly135 | 3.49 | 2.5 | 150 | 157 | 22 | 94 | 162 | 14 |
| 14 | 2XHR | Heat shock protein HSP 90-alpha | CH_2_(C14) | Gly135 | 3.55 | 2.52 | 156 | 133 | 23 | 92 | 137 | 16 |
| 15 | 3FUI | Leukotriene A4 hydrolase | CH_2_(C1S) | Gly269 | 3.24 | 2.48 | 126 | 129 | 31 | 147 | 143 | 17 |
| 16 | 3GWS | T3-bound thyroid hormone receptor | CH(C15) | Met313 | 3.54 | 2.49 | 160 | 127 | 11 | 104 | 126 | 17 |
| 17 | 3KM4 | Renin | CH_3_(C28) | Met16 | 3.15 | 2.5 | 117 | 127 | 44 | 108 | 144 | 33 |
| 18 | 3NN6 | 6-Hydroxy-L-nicotine oxidase | CH_2_(C10) | Gly406 | 3.17 | 2.54 | 116 | 123 | 49 | 157 | 137 | 42 |
| 19 | 3NN6 | 6-Hydroxy-L-nicotine oxidase | CH_3_(C12) | Met167 | 3.4 | 2.43 | 148 | 131 | 12 | 131 | 131 | 2 |
| 20 | 3NWW | P38 Alpha kinase | CH_3_(C26) | Ser38 | 3.26 | 2.2 | 164 | 145 | 4 | 118 | 146 | 1 |
| 21 | 3PAH | Phenylalanine hydroxylase | CH_3_(C9) | Leu249 | 3.08 | 2.52 | 110 | 152 | 4 | 117 | 143 | 24 |
| 22 | 4ABZ | TETR(D) | CH(C4) | Gln116 | 3.59 | 2.58 | 153 | 117 | 26 | 113 | 113 | 20 |
| 23 | 4BKY | Maternal embryonic leucine zipper kinase | CH_2_(326) | Asn137 | 3.37 | 2.31 | 164 | 147 | 32 | 122 | 143 | 37 |
| 24 | 4CGO | Glycylpeptide *N*-tetradecanoyltransferase | CH_3_(C14) | Asn167 | 3.08 | 2.27 | 130 | 129 | 45 | 113 | 115 | 51 |
| 25 | 4CGO | Glycylpeptide *N*-tetradecanoyltransferase | CH_3_(C14) | Phe232 | 3.16 | 2.37 | 128 | 137 | 42 | 112 | 152 | 28 |
| 26 | 4CP9 | PA-I galactophilic lectin | CH_2_(C26) | Gln40 | 3.05 | 2.28 | 126 | 132 | 44 | 75 | 116 | 60 |
| 27 | 4G0V | DNA topoisomerase 2-beta | CH_2_(CAM) | Lys505 | 3.07 | 2.44 | 115 | 166 | 12 | 150 | 154 | 23 |
| 28 | 4G0V | DNA topoisomerase 2-beta | CH_2_(CAP) | Arg503 | 3.13 | 2.07 | 162 | 175 | 3 | 120 | 175 | 5 |
| 29 | 4GOL | DNA adenine methylase | CH_2_(CG) | Asp181 | 3.12 | 2.12 | 152 | 133 | 47 | 89 | 142 | 37 |
| 30 | 4GOL | DNA adenine methylase | CH_3_(CE1) | Ala11 | 3.34 | 2.36 | 148 | 121 | 59 | 131 | 122 | 56 |
| 31 | 4JRV | EGFR kinase | CH_3_(C36) | Asn818 | 3.32 | 2.32 | 151 | 115 | 22 | 92 | 123 | 16 |
| 32 | 4JRV | EGFR kinase | CH_2_(C34) | Asp813 | 3.37 | 2.6 | 127 | 139 | 25 | 90 | 153 | 13 |
| 33 | 4KMN | Baculoviral IAP repeat-containing protein 2 | CH(CA1) | Arg308 | 3.27 | 2.55 | 123 | 146 | 21 | 150 | 156 | 24 |
| 34 | 4L02 | Sphingosine kinase 1 | CH_2_(C22) | Leu268 | 3.52 | 2.52 | 153 | 158 | 22 | 98 | 164 | 16 |
| 35 | 4MDD | Glucocorticoid receptor | CH_2_(C15) | Leu563 | 3.39 | 2.34 | 163 | 107 | 62 | 116 | 104 | 67 |
| 36 | 4MDD | Glucocorticoid receptor | CH_3_(C16) | Asn564 | 3.59 | 2.53 | 164 | 109 | 21 | 118 | 112 | 17 |
| 37 | 4MQF | Gamma-aminobutyric acid type B receptor subunit 2 | CH_2_(C8) | Aly151 | 3.34 | 2.57 | 127 | 131 | 1 | 111 | 146 | 1 |
| 38 | 4U5N | Importin subunit alpha-1 | CH_2_(CE) | Gly323 | 3.23 | 2.42 | 130 | 161 | 17 | 96 | 157 | 22 |

**Table S4. Results of the PDB survey on peptide bond/Asn/Gln hydrogen acceptors (continued)**.

| Entry | PDB code | Protein | Carbon | Acceptor | C···O distance  (*d*_CO_) | H···O distance (*d*_HO_) | C-H···O  angle (**) | H···O=C angle (**) | H-  elevation angle (*θ*) | N^+^-C···O angle (*α*) | C···O=C angle (*β*) | C-  elevation angle (*φ*) |
| --- | --- | --- | --- | --- | --- | --- | --- | --- | --- | --- | --- | --- |
| 39 | 4U6R | Endoribonuclease IRE1 | CH(C23) | Ala646 | 3.61 | 2.56 | 160 | 124 | 41 | 123 | 120 | 47 |
| 40 | 4UVU | Tankyrase-2 | CH_2_(C16) | Ala1049 | 2.86 | 2.19 | 117 | 142 | 22 | 146 | 140 | 36 |
| 41 | 4WKN | Aminodeoxyfutalosine nucleosidase | CH_2_(C10) | Val78 | 3.14 | 2.22 | 140 | 108 | 69 | 119 | 117 | 56 |
| 42 | 4X8E | Sulfoxide synthase EgtB | CH_3_(CAC) | Glu137 | 3.33 | 2.62 | 130 | 139 | 27 | 94 | 152 | 19 |
| 43 | 4X8E | Sulfoxide synthase EgtB | CH_3_(CAB) | Glu137 | 3.34 | 2.65 | 127 | 141 | 17 | 93 | 154 | 13 |
| 44 | 4X8E | Sulfoxide synthase EgtB | CH_3_(CAC) | Asn414 | 3.5 | 2.61 | 150 | 125 | 53 | 97 | 133 | 45 |
| 45 | 4YML | 5'-Methylthioadenosine/  S-adenosylhomocysteine nucleosidase | CH_2_(C10) | Ser76 | 3.32 | 2.24 | 171 | 111 | 65 | 106 | 108 | 68 |
| 46 | 4YND | *N*-Lysine methyltransferase SMYD2 | CH_2_(C26) | Gly183 | 3.35 | 2.34 | 153 | 119 | 55 | 100 | 121 | 47 |
| 47 | 4YND | *N*-Lysine methyltransferase SMYD2 | CH_2_(C28) | Gly183 | 3.52 | 2.52 | 152 | 144 | 0 | 93 | 142 | 8 |
| 48 | 4ZEG | TTK kinase | CH_2_(C4) | Asn606 | 3.27 | 2.2 | 165 | 146 | 30 | 99 | 145 | 33 |
| 49 | 4ZFL | Amidohydrolase EgtC | CH_3_(CAB) | Ala90 | 3.2 | 2.51 | 122 | 165 | 10 | 119 | 166 | 13 |
| 50 | 5AJW | 6-Phosphofructo-2-kinase/fructose-2,6-  bisphosphatase 3 | CH_2_(C23) | Gly218 | 3.38 | 2.47 | 140 | 142 | 18 | 131 | 131 | 19 |
| 51 | 5AM6 | Fibroblast growth factor receptor 1 | CH_2_(C3) | Ser565 | 2.99 | 2.39 | 113 | 176 | 3 | 156 | 157 | 9 |
| 52 | 5AYF | SET7/9 | CH_2_(C18) | Gly264 | 3.09 | 2.28 | 130 | 142 | 31 | 136 | 139 | 40 |
| 53 | 5C0K | E3 ubiquitin-protein ligase XIAP | CH(C8) | Thr308 | 3.19 | 2.52 | 125 | 148 | 18 | 147 | 156 | 21 |
| 54 | 5CI7 | Serine/threonine-protein kinase ULK1 | CH_2_(C02) | Ile22 | 3.16 | 2.26 | 155 | 145 | 16 | 119 | 149 | 20 |
| 55 | 5DQC | Beta-secretase 1 | CH(C24) | Gly34 | 3.36 | 2.34 | 154 | 132 | 24 | 111 | 139 | 25 |
| 56 | 5EAM | WD repeat-containing protein 5 | CH_3_ | Ser91 | 3.28 | 2.29 | 150 | 150 | 14 | 89 | 152 | 21 |
| 57 | 5EMJ | Protein arginine *N*-methyltransferase 5 | CH_2_(CAV) | Ser578 | 3.18 | 2.65 | 109 | 125 | 26 | 104 | 122 | 44 |
| 58 | 5EMJ | Protein arginine *N*-methyltransferase 5 | CH_2_(CAT) | Ser578 | 3.26 | 2.66 | 114 | 139 | 35 | 101 | 154 | 25 |
| 59 | 5HHW | Insulin receptor | CH_2_(C23) | Gln1031 | 3.58 | 2.57 | 154 | 129 | 51 | 117 | 130 | 49 |
| 60 | 5HI7 | Human SMYD3 | CH_2_(C8) | Ser202 | 3.05 | 2.25 | 129 | 160 | 20 | 104 | 173 | 4 |
| 61 | 5HI7 | Human SMYD3 | CH_2_(C4) | Cys180 | 3.25 | 2.18 | 167 | 135 | 12 | 114 | 136 | 16 |
| 62 | 5HI7 | Human SMYD3 | CH_2_(C6) | Ser202 | 3.31 | 2.47 | 133 | 132 | 19 | 93 | 141 | 6 |
| 63 | 5IYZ | Tubulin beta-2B chain | CH_3_(CAH) | Phe351 | 3.18 | 2.21 | 147 | 138 | 30 | 101 | 148 | 26 |
| 64 | 5JYZ | Tubulin beta-2B chain | CH(CA) | Asn329 | 3.46 | 2.54 | 142 | 135 | 44 | 128 | 136 | 39 |
| 65 | 5LJ1 | Bromodomain-containing protein 4 | CH_2_(C08) | Asn140 | 3.33 | 2.29 | 158 | 126 | 16 | 105 | 132 | 12 |

**Table S4. Results of the PDB survey on Asp/Glu hydrogen acceptors.**

| Entry | PDB code | Protein | Carbon | Acceptor | C···O distance  (*d*_CO_) | H···O distance (*d*_HO_) | C-H···O  angle (**) | H···O-C angle (**) | H-  elevation angle (*θ*) | N^+^-C···O angle (*α*) | C···O-C angle (*β*) | C-  elevation angle (*φ*) |
| --- | --- | --- | --- | --- | --- | --- | --- | --- | --- | --- | --- | --- |
| 1 | 1BO4 | GCN5-related *N*-acetyltransferase | CH_2_(C5) | Asp110 | 2.72 | 2.06 | 116 | 210 | 21 | 117 | 142 | 7 |
| 2 | 1D6Q | Squalene-hopene cyclas | CH_2_(C1B) | Asp374 | 3.48 | 2.42 | 166 | 244 | 56 | 105 | 114 | 60 |
| 3 | 1OHR | Aspartylprotease | CH_2_(C10) | Asp25 | 3.41 | 2.43 | 149 | 217 | 31 | 111 | 134 | 40 |
| 4 | 1WRK | Human cardiac troponin C | CH_2_(C15) | Glu19 | 3.06 | 2.2 | 135 | 230 | 28 | 96 | 131 | 38 |
| 5 | 1XH0 | Human pancreatic alpha-amylase | CH(C1H) | Glu233 | 3.04 | 2.04 | 152 | 232 | 41 | 90 | 120 | 41 |
| 6 | 2OO8 | Tie-2 | CH_2_(C27) | Asp982 | 3.05 | 2.04 | 154 | 101 | 77 | 100 | 109 | 71 |
| 7 | 2Q8C | JMJD2A | CH_3_(CM1) | Glu190 | 3.3 | 2.24 | 165 | 206 | 23 | 118 | 156 | 23 |
| 8 | 2VNW | Protein Kinase B | CH_2_(C9) | Glu127 | 3.34 | 2.43 | 141 | 114 | 59 | 130 | 108 | 56 |
| 9 | 3APR | Fhizopuspepsin | CH_2_(C2) | Asp35 | 3.29 | 2.51 | 127 | 249 | 11 | 143 | 101 | 23 |
| 10 | 3APR | Rhizopuspepsin | CH_2_(C2) | Asp218 | 3.57 | 2.67 | 140 | 256 | 0 | 87 | 97 | 9 |
| 11 | 3MO0 | Human G9a-like protein | CH_3_(CAU) | Asp1131 | 1.97 | 1.48 | 99 | 136 | 11 | 110 | 166 | 4 |
| 12 | 3MO0 | Human G9a-like protein | CH_3_(CAS) | Asp1131 | 2.68 | 2.33 | 96 | 247 | 61 | 80 | 131 | 37 |
| 13 | 3MO0 | Human G9a-like protein | CH_3_(CAS) | Asp1135 | 3.06 | 2.3 | 125 | 132 | 36 | 74 | 116 | 42 |
| 14 | 3NWW | P38 alpha kinase | CH_3_(C24) | Asp168 | 2.85 | 2.11 | 122 | 144 | 31 | 74 | 146 | 34 |
| 15 | 3NWW | P38 alpha kinase | CH_3_(C28) | Asp168 | 3.05 | 2.4 | 117 | 150 | 25 | 67 | 151 | 9 |
| 16 | 3WC7 | Carboxypeptidase B | CH_2_(C2) | Asp255 | 3.11 | 2.3 | 130 | 235 | 31 | 79 | 110 | 34 |
| 17 | 3WZ6 | Endothiapepsin | CH_2_(C19) | Asp35 | 3.05 | 2.59 | 104 | 260 | 13 | 84 | 102 | 7 |
| 18 | 3WZ6 | Endothiapepsin | CH_2_(C19) | Asp219 | 3.21 | 2.43 | 127 | 264 | 3 | 144 | 105 | 11 |
| 19 | 4C39 | Nitric oxide synthase | CH_2_(C5') | heme | 2.82 | 2.17 | 115 | 199 | 18 | 79 | 146 | 31 |
| 20 | 4C7F | Beta-*N*-acetylhexosaminidase | CH_2_(C9) | Glu302 | 3.00 | 2.37 | 115 | 203 | 23 | 67 | 143 | 31 |
| 21 | 4CGO | Glycylpeptide *N*-tetradecanoyltransferase | CH_2_(C13) | Asp396 | 3.33 | 2.31 | 155 | 260 | 64 | 103 | 104 | 56 |
| 22 | 4G0V | DNA topoisomerase 2-beta | CH_2_(CAP) | Glu522 | 3.14 | 2.21 | 142 | 248 | 26 | 110 | 110 | 38 |
| 23 | 4I71 | Inosine-adenosine-guanosine-nucleoside hydrolase | CH_2_(C7) | Asp40 | 3.3 | 2.55 | 126 | 253 | 72 | 82 | 123 | 57 |
| 24 | 4I71 | Inosine-adenosine-guanosine-nucleoside hydrolase | CH_2_(C1') | Asp40 | 3.38 | 2.52 | 135 | 170 | 1 | 80 | 164 | 12 |
| 25 | 4KQP | Alpha-L-fucosidase | CH(CAB) | Glu288 | 3.23 | 2.52 | 122 | 251 | 70 | 70 | 105 | 62 |
| 26 | 4MQ4 | Phenylethanolamine *N*-methyltransferase | CH_2_(C27) | Asp267 | 3.56 | 2.66 | 150 | 224 | 36 | 90 | 132 | 44 |
| 27 | 4OBQ | Mitogen-activated protein kinase 4 | CH_2_(C22) | Asp171 | 3.44 | 2.48 | 147 | 255 | 63 | 100 | 96 | 62 |
| 28 | 4Q3S | Arginase | CH_2_(C13) | Asp211 | 3.57 | 2.63 | 144 | 240 | 59 | 114 | 123 | 53 |
| 29 | 4QNQ | Bcl-2-like protein 1 | CH_2_(C35) | Glu96(L) | 2.88 | 2.01 | 134 | 231 | 13 | 86 | 114 | 11 |
| 30 | 4RVX | A02 HIV-1 protease | CH(C14) | Asp29 | 3.58 | 2.54 | 159 | 134 | 38 | 122 | 131 | 37 |
| 31 | 4RYZ | Retinoid isomerohydrolase | CH_2_ | Glu148 | 3.37 | 2.51 | 135 | 261 | 44 | 82 | 99 | 57 |
| 32 | 4UIB | Carboxypeptidase B | CH_2_(C23) | Asp255 | 3.33 | 2.54 | 128 | 250 | 7 | 75 | 101 | 20 |
| 33 | 4WH5 | Lincosamide resistance protein | CH(C11) | Asp28 | 3.01 | 1.96 | 160 | 256 | 40 | 111 | 110 | 36 |
| 34 | 4WH5 | Lincosamide resistance protein | CH_2_(C14) | Asp50 | 3.67 | 2.68 | 151 | 265 | 55 | 96 | 103 | 53 |
| 35 | 4WKN | Aminodeoxyfutalosine nucleosidase | CH_2_(C2') | Glu175 | 3.58 | 2.57 | 154 | 257 | 12 | 99 | 102 | 4 |

**Table S5. Results of the PDB survey on Asp/Glu hydrogen acceptors (continued).**

| Entry | PDB code | Protein | Carbon | Acceptor | C···O distance  (*d*_CO_) | H···O distance (*d*_HO_) | C-H···O  angle (**) | H···O-C angle (**) | H-  elevation angle (*θ*) | N^+^-C···O angle (*α*) | C···O-C angle (*β*) | C-  elevation angle (*φ*) |
| --- | --- | --- | --- | --- | --- | --- | --- | --- | --- | --- | --- | --- |
| 36 | 4XCZ | TDP-3-aminoquinovose-N-  formyltransferase | CH(C3Q) | Glu75 | 3.67 | 2.62 | 165 | 254 | 46 | 101 | 102 | 48 |
| 37 | 4YML | 5'-Methylthioadenosine/  S-adenosylhomocysteine nucleosidase | CH_2_(C2') | Glu12 | 3.24 | 2.7 | 110 | 254 | 41 | 110 | 120 | 34 |
| 38 | 4ZOL | Tubulin alpha-1B chain | CH(CA) | Asp179 | 2.93 | 1.94 | 148 | 216 | 22 | 87 | 145 | 12 |
| 39 | 4ZOL | Tubulin alpha-1B chain | CH_2_(CAG) | Asp179 | 3.1 | 2.22 | 136 | 246 | 48 | 81 | 122 | 35 |
| 40 | 4ZOL | Tubulin alpha-1B chain | CH_2_(CAH) | Asp179 | 3.39 | 2.64 | 126 | 255 | 14 | 108 | 99 | 0 |
| 41 | 5AI9 | Bifunctional epoxide hydrolase 2 | CH_2_(C13) | Asp335 | 3.01 | 2.46 | 110 | 170 | 4 | 63 | 169 | 2 |
| 42 | 5AI9 | Bifunctional epoxide hydrolase 2 | CH_2_(C15) | Asp335 | 3.02 | 2.69 | 97 | 235 | 54 | 62 | 126 | 43 |
| 43 | 5C1M | Mu-type opioid receptor | CH_2_(CAO) | Asp147 | 2.97 | 2.65 | 96 | 117 | 62 | 63 | 135 | 45 |
| 44 | 5DFP | Serine/threonine-protein kinase PAK 1 | CH_3_ | Asp354 | 3.13 | 2.46 | 118 | 237 | 24 | 94 | 106 | 33 |
| 45 | 5DM0 | Plantazolicin methyltransferase BamL | CH(CA) | Asp34 | 3.56 | 2.6 | 146 | 267 | 28 | 84 | 92 | 38 |
| 46 | 5DQC | Beta-secretase 1 | CH_2_(C22) | Asp228 | 2.87 | 2.17 | 120 | 260 | 20 | 106 | 111 | 4 |
| 47 | 5DSG | M1 and M4 muscarinic acetylcholine receptors | CH_3_(C12) | Asp112 | 3.49 | 2.6 | 138 | 114 | 48 | 138 | 103 | 56 |
| 48 | 5HJ9 | Human Arginase-1 | CH_2_(C11) | Asp194 | 3.39 | 2.63 | 132 | 269 | 27 | 136 | 104 | 28 |
| 49 | 5HJ9 | Human Arginase-1 | CH_2_(C11) | Asp194 | 3.65 | 2.65 | 173 | 270 | 27 | 111 | 91 | 26 |
| 50 | 5L2T | Cyclin-dependent kinase 6 | CH_2_(C29) | Asp104 | 3.53 | 2.57 | 146 | 262 | 42 | 101 | 101 | 51 |

**Table S6. Results of the PDB survey on Ser/Thr/Tyr hydrogen acceptors.**

| Entry | PDB code | Protein | Carbon | Acceptor | C···O distance  (*d*_CO_) | H···O distance (*d*_HO_) | C-H···O  angle (**) | H···O-C angle (**) | H-  elevation angle (*θ*) | N^+^-C···O angle (*α*) | C···O-C angle (*β*) | C-  elevation angle (*φ*) |
| --- | --- | --- | --- | --- | --- | --- | --- | --- | --- | --- | --- | --- |
| 1 | 1BO4 | GCN5-related *N*-acetyltransferase | CH_2_(C7) | Tyr109 | 3.48 | 2.46 | 156 | 140 | - | 125 | 144 | - |
| 2 | 1EL5 | Sarcosine oxidase | CH_3_(C5) | Tyr317 | 3.31 | 2.43 | 137 | 115 | - | 133 | 128 | - |
| 3 | 1UDT | cGMP-specific 3',5'-cyclic Phosphodiesterase | CH_2_(C16) | Tyr664 | 3.19 | 2.20 | 149 | 110 | - | 119 | 119 | - |
| 4 | 2Q8C | JMJD2A | CH_3_(CM3) | Tyr177 | 3.16 | 2.16 | 151 | 110 | - | 90 | 116 | - |
| 5 | 2Q8C | JMJD2A | CH_3_(CM3) | Ser288 | 2.98 | 2.16 | 130 | 108 | - | 101 | 124 | - |
| 6 | 2Q8C | JMJD2A | CH_3_(CM2) | Tyr177 | 3.35 | 2.42 | 142 | 115 | - | 84 | 121 | - |
| 7 | 2Q8C | JMJD2A | CH_3_(CM2) | Ser288 | 3.08 | 2.33 | 124 | 108 | - | 97 | 124 | - |
| 8 | 3APV | Alpha-1-acid glycoprotein 2 | CH_2_(C14) | Ser125 | 3.41 | 2.55 | 135 | 115 | - | 133 | 121 | - |
| 9 | 3ZDH | Acetylcholine binding protein | CH_3_(C1) | Tyr89 | 3.16 | 2.36 | 128 | 128 | - | 138 | 129 | - |
| 10 | 4ABZ | TETR(D) | CH_3_(C43) | Ser138 | 3.49 | 2.51 | 150 | 117 | - | 98 | 123 | - |
| 11 | 4JE5 | Aromatic/aminoadipate aminotransferase 1 | CH_2_(C4A) | Tyr251 | 3.33 | 2.63 | 122 | 146 | - | 150 | 130 | - |
| 12 | 4MN3 | Chromobox protein homolog 7 | CH_3_(CM3) | Tyr33 | 3.37 | 2.46 | 141 | 139 | - | 134 | 135 | - |
| 13 | 4QF9 | Glutamate receptor ionotropic, kainate 1 | CH(C11) | Ser193 | 3.07 | 2.31 | 125 | 144 | - | 79 | 154 | - |
| 14 | 5AYF | SET7/9 | CH_3_(C21) | Tyr245 | 3.49 | 2.47 | 156 | 106 | - | 117 | 100 | - |
| 15 | 5AYF | SET7/9 | CH_3_(C21) | Tyr305 | 3.13 | 2.29 | 132 | 114 | - | 134 | 101 | - |
| 16 | 5CFT | Aminoglycoside Nucleotidyltransferase (2")-Ia | CH(C15) | Tyr74 | 3.47 | 2.42 | 161 | 138 | - | 113 | 134 | - |
| 17 | 5DQC | Beta-secretase 1 | CH_2_(C22) | Thr231 | 3.28 | 2.31 | 147 | 150 | - | 104 | 155 | - |
| 18 | 5FHM | Glutamate receptor 2,Glutamate receptor 2 | CH(C25) | Ser142 | 3.30 | 2.46 | 132 | 149 | - | 118 | 136 | - |
| 19 | 5HBG | Cysteine synthase | CH(CA) | Thr77 | 3.04 | 2.10 | 142 | 117 | - | 89 | 104 | - |
| 20 | 5HI7 | Human SMYD3 | CH_2_(C15) | Tyr239 | 3.52 | 2.52 | 152 | 111 | - | 90 | 113 | - |

**Table S7. Interaction energies for A1, B1, C1, and the H_2_O dimer calculated at the M06-2X/6-311++G** or MP2/aug-cc-pVTZ level of theory.**

| Complex | M06-2X/6-311++G** | MP2/aug-cc-pVTZ |
| --- | --- | --- |
| **A1** | -22.01 kcal/mol | -20.39 kcal/mol |
| **B1** | -106.20 kcal/mol | -103.40 kcal/mol |
| **C1** | -13.49 kcal/mol | -11.90 kcal/mol |
| H_2_O dimer | -5.71 kcal/mol | -4.79 kcal/mol |

**Figure S1.** (A, B) Examples for C-H···Y hydrogen bonds that are activated by a cationically charged atom. (A) Proposed substrate recognition by a tetraalkylammonium salt in Mannich-type reactions. (B) Proposed substrate recognition of histone demethylase JMJD2A: *N*,*N*-dimethyllysine (left) and *N*-methyllysine at the active site of JMJD2A (right). It has been proposed that histone demethylases recognize the number of methyl groups by C-H···O hydrogen bonds between their N^+^-C-H group and the oxygen atoms of serine, tyrosine, or glutamate residues.(C, D) Working hypotheses for the formation of C-H···O hydrogen bonds between proteins and ligands that bear (C) tetraalkylammonium or (D) protonated aliphatic amine moieties.


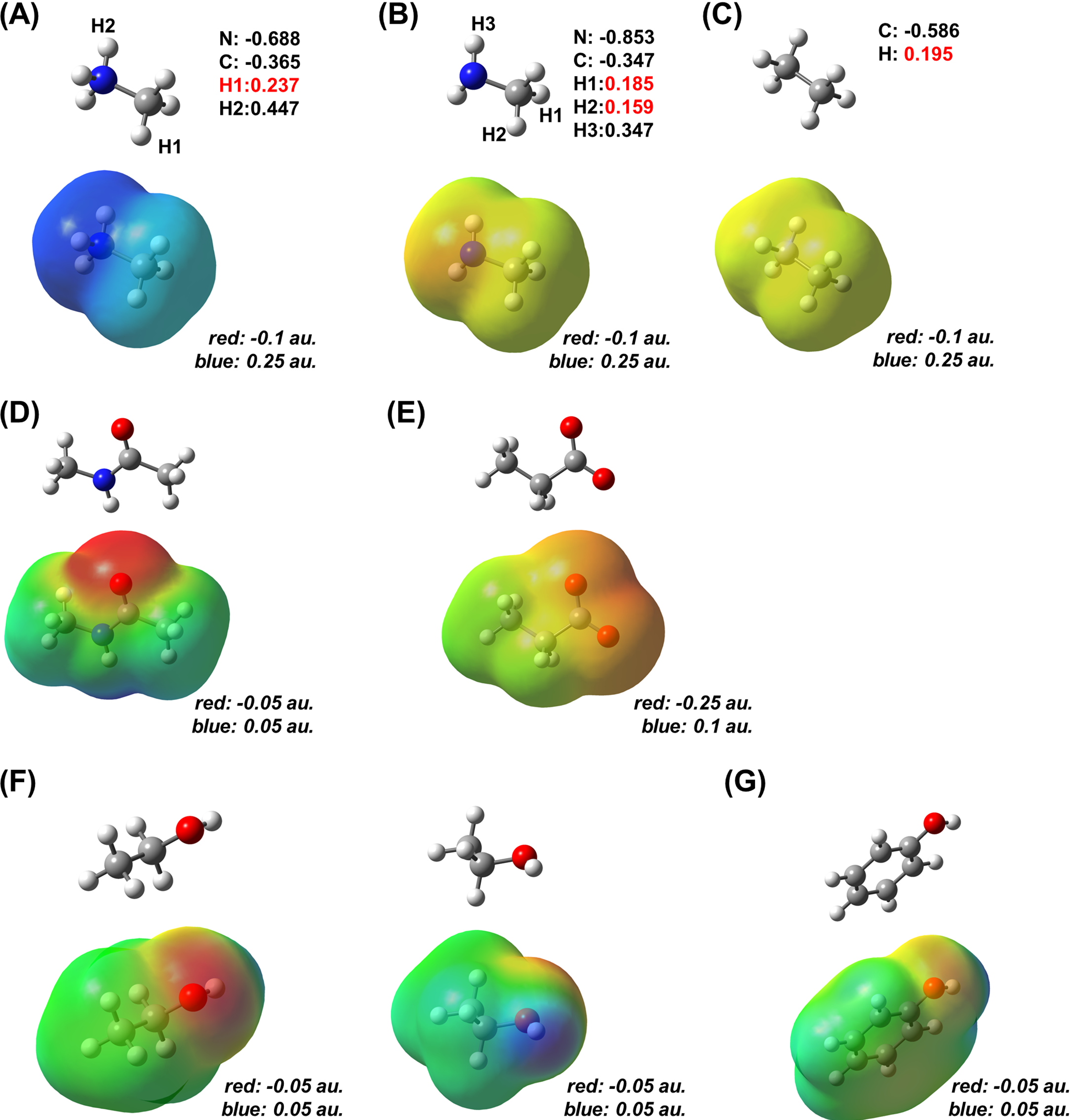


**Figure S2.** Formal charges and/or electrostatic potential maps for **1**–**5**, **8** and **9**. Structures were optimized at the M06-2X/6-311++G** level of theory. Formal charges were estimated by natural bond orbital (NBO) analysis. The contour ranges of the potential maps differ for the individual compounds (**5**, **8**, and **9**: -0.1–0.25 au; **1**, **3**, and **4**: -0.05–0.05 au; **2**: -0.25–0.1 au). Red and blue areas indicate least and most positively charged regions, respectively. (A) Monomethylammoium (**5**). (B) Mnomethylamine (**8**). (C) Ethane (**9**). (D) *N*-Methylacetamide (**1**). (E) Propanoate (**2**). (F) Ethanol (**3**), the map of **3** is shown from two different perspectives. (G) Phenol (**4**).

**
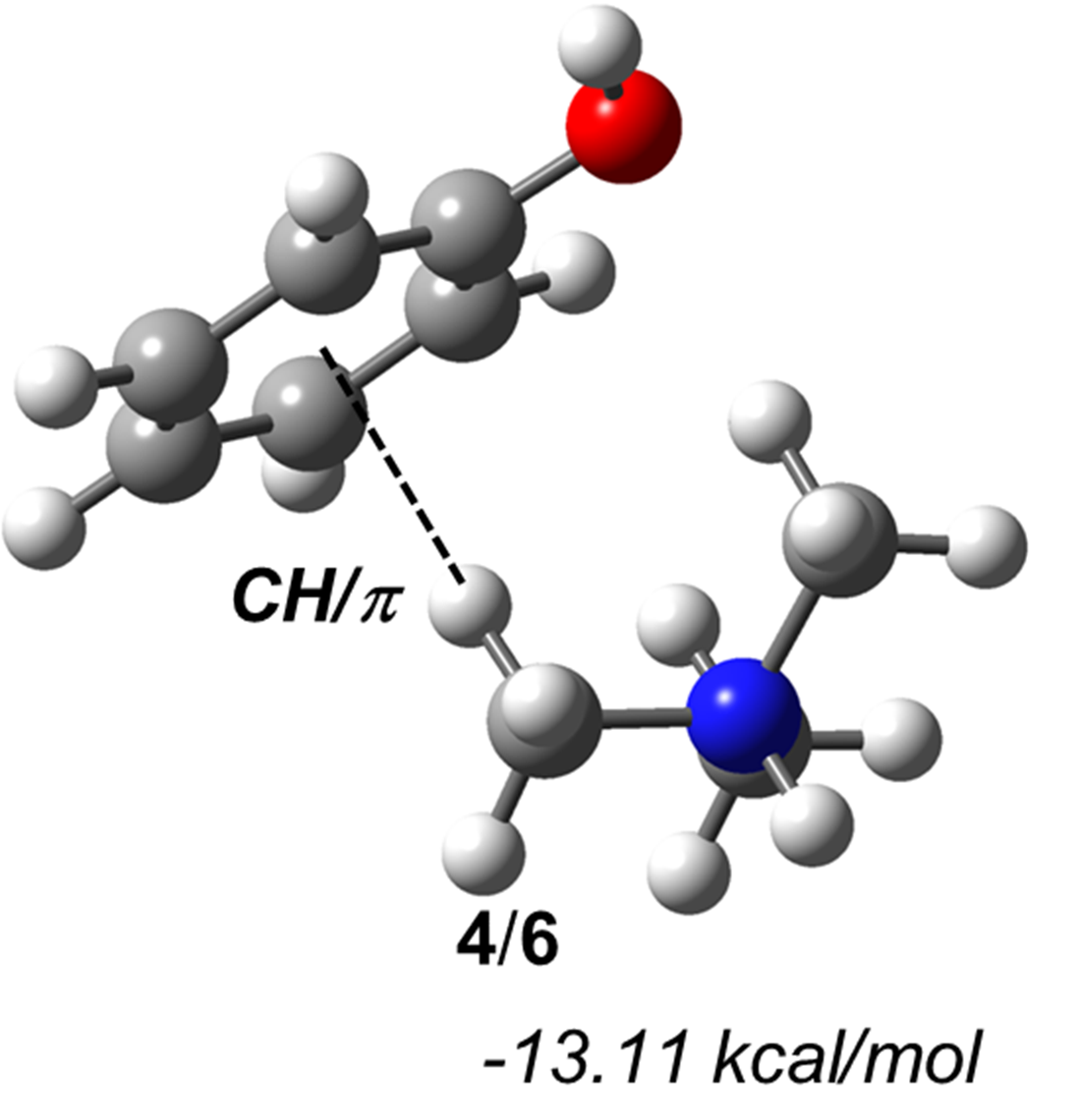
**

**Figure S3.** Optimized geometries for trimethylammonium (**7**) complexed with phenol (**4**). The optimized geometry was calculated at the M06-2X/6-311++G** level of theory and the interaction energy was corrected for basis set superposition errors (BSSE) by counterpoise correction.


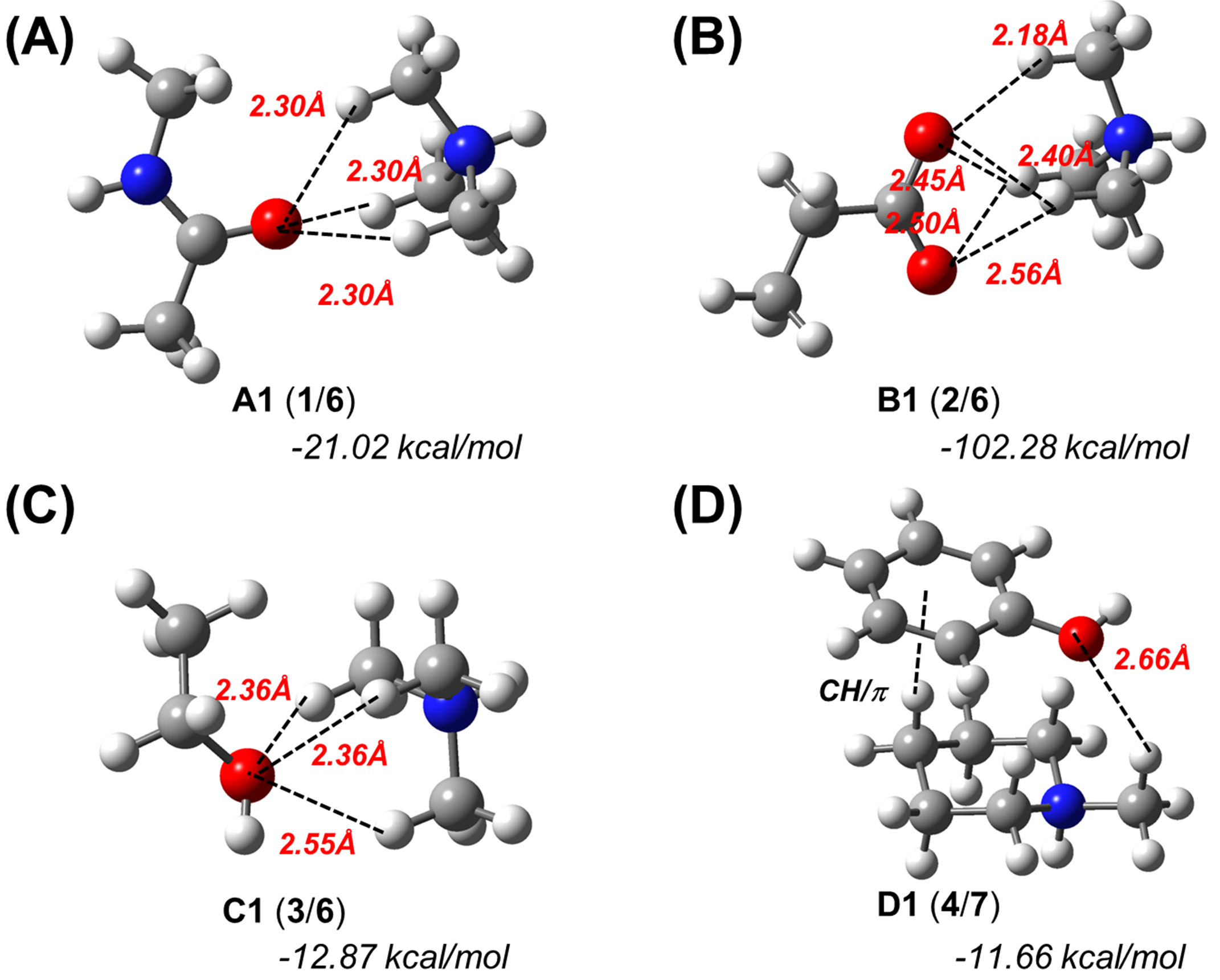


**Figure S4.** Theoretically optimized geometries and counterpoise-corrected interaction energies for N^+^-C-H··O hydrogen bond models. The geometry optimizations and energy calculations were carried out at the M06-2X/6-311++G** level of theory in water. (A) Trimethylammonium (**6**) complexed with *N*-methylacetamide (**1**). (B) **6** complexed with propanoate (**2**). (C) **6** complexed with ethanol (**3**). (D) *N*-methylpiperidium (**7**) complexed with phenol (**4**). Compared with the geometries and energies of the simple complexes **A1**−**D1** (Fig. 1 in the main text), those of **A1**−**D1** in the ternary complexes are slightly higher and longer, respectively. These results suggest that the solvation in water a relatively limited impact on the N^+^-C-H··O hydrogen bonds.


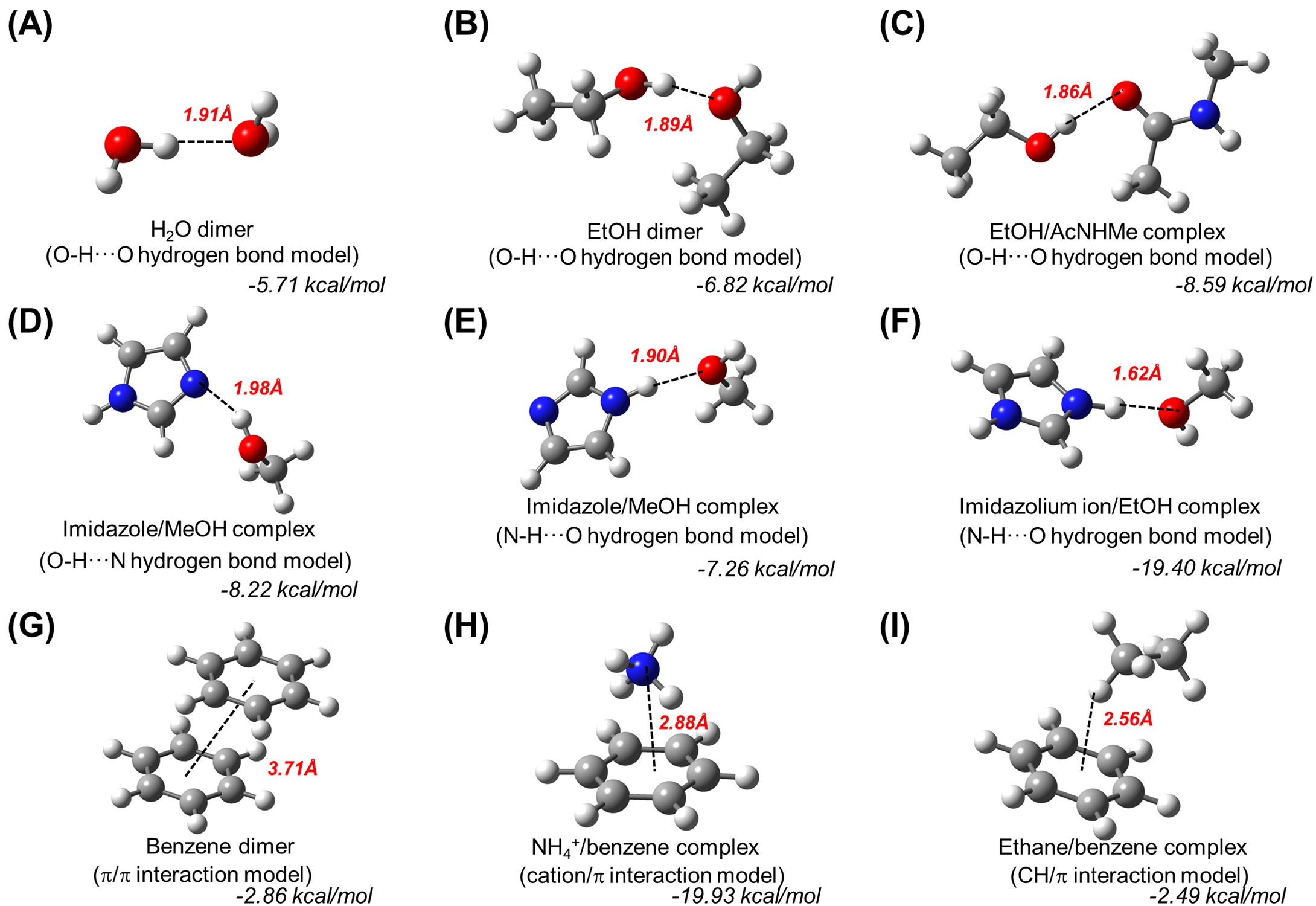


**Figure S5.** Optimized geometries and interaction energies for heteroatom-hydrogen bond, / interaction, cation/ interaction, or CH/ interaction models. Optimized structures were calculated at the M06-2X/6-311++G** level of theory. Interaction energies were corrected for BSSE by counterpoise correction. The energies of the N^+^-C-H···O hydrogen bonds are low relative to those of heteroatom-hydrogen bonds, / interactions, cation/ interactions, or CH/πother interaction models. (A) H_2_O dimer for a heteroatom-hydrogen bond (O-H···O hydrogen bond) model. (B) EtOH dimer for a heteroatom-hydrogen bond (O-H···O hydrogen bond) model. (C) EtOH/AcNHMe complex for a heteroatom-hydrogen bond (N-H···O hydrogen bond) model. (D) Imidazole/MeOH complex for a heteroatom-hydrogen bond (O-H···N hydrogen bond) model. (E)Imidazole/MeOH complex for a heteroatom-hydrogen bond (N-H···O hydrogen bond). (F) Imidazolium ion/MeOH complex for a heteroatom-hydrogen bond (N-H···H hydrogen bond) model. (G)Benzene dimer for a / interaction model. (H) NH_4_^+^/benzene complex for a cation/ interaction model.(I) Ethane/benzene complex for a CH/ interaction model.


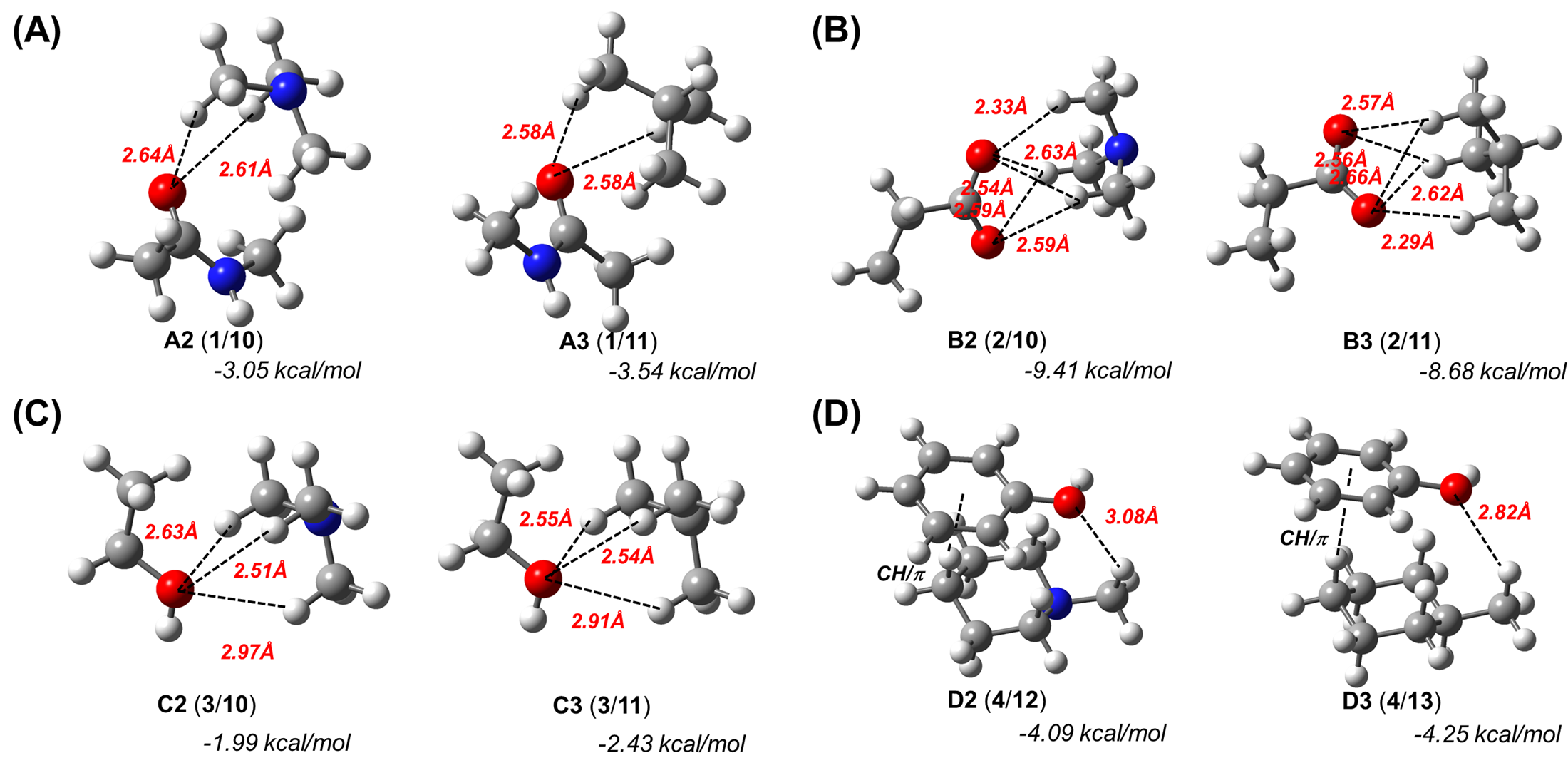


**Figure S6.** Theoretically optimized geometries and counterpoise-corrected interaction energies of **10**–**13** complexed with **1**–**4.** Geometry optimizations and energy calculations were carried out at the M06-2X/6-311++G** level of theory. (A) Triethylamine (**10**) and 2,2-dimethylpropane (**11**) complexed with *N*-methylacetamide (**1**). (B) **10** and **11** complexed with propanoate (**2**). (C) **10** and **11** complexed with ethanol (**3**).(D) *N*-methylpiperidine (**12**) and methylcyclohexane (**13**) complexed with phenol (**4**).


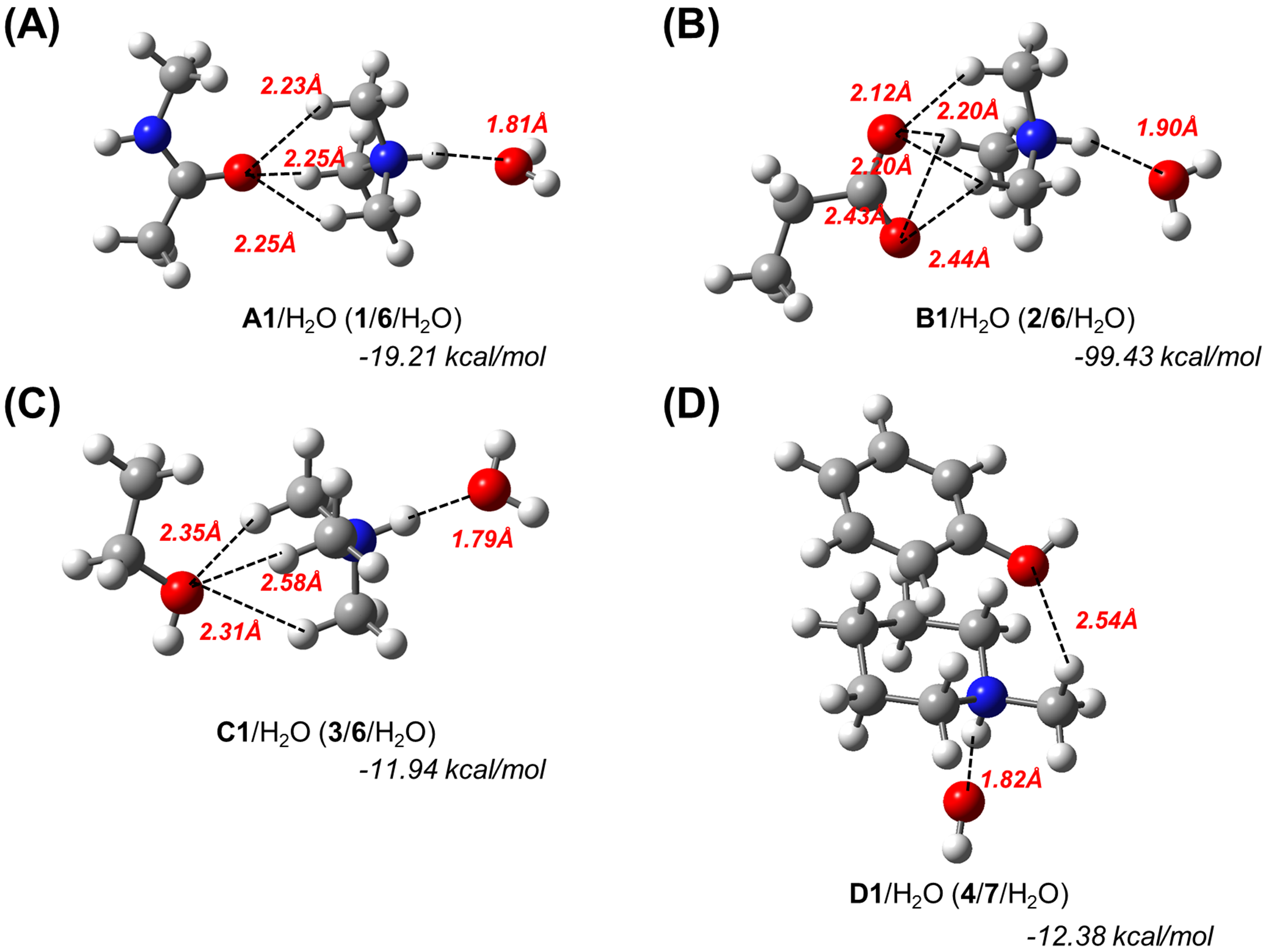


**Figure S7.** Theoretically optimized geometries and counterpoise-corrected interaction energies for N^+^-C-H··O hydrogen bond models. The geometry optimizations and energy calculations were carried out at the M06-2X/6-311++G** level of theory. (A) Trimethylammonium (**6**) complexed with a water molecule and *N*-methylacetamide (**1**). (B) **6** complexed with a water molecule andpropanoate (**2**). (C) **6** complexed with a water molecule andethanol (**3**). (D) *N*-methylpiperidium (**7**) complexed with a water molecule andphenol (**4**). The interaction energies between **A1**−**D1** and **6**/water are indicated.　Compared with the geometries and energies of the simple complexes **A1**−**D1** (Fig. 1 in the main text), those of **A1**−**D1** in the ternary complexes are slightly higher and longer, respectively. These results suggest that the formation of even ideal heteroatom-hydrogen bonds of the ammonium cations exert a relatively limited effect on the N^+^-C-H··O hydrogen bonds.

**
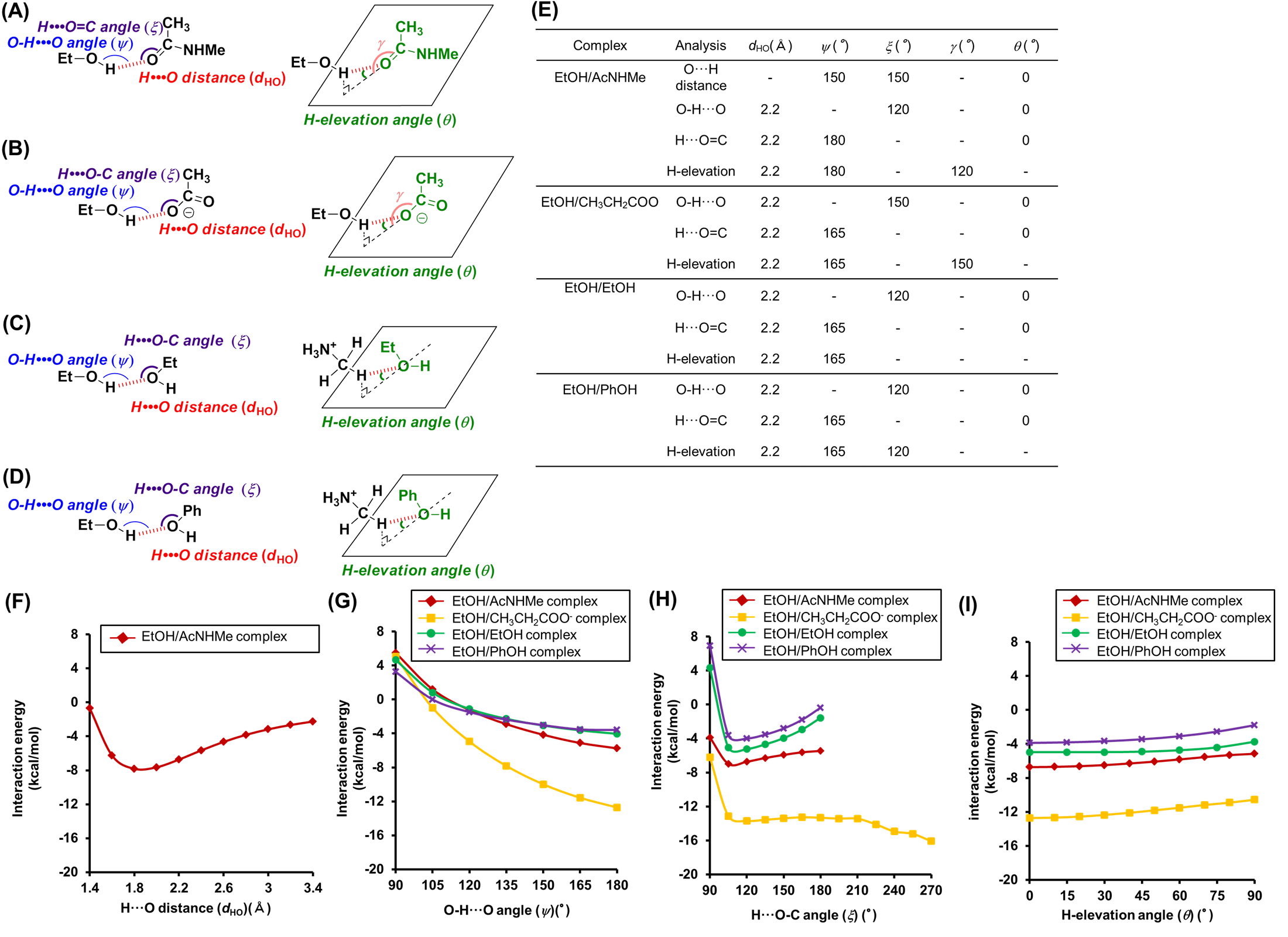
**

**Figure S8.** Theoretical analysis of the dependence of the interaction energies on the geometry of the O-H···O hydrogen bonds between **1**–**4** and ethanol (**3**). (A–D) Geometry of the O-H···O hydrogen bond models. (A) A complex of *N*-methylacetamide (**1**) with **3**.(B) A complex of propanoate (**2**) with **3**.(C) Ethanol (**3**) dimer. (D) A complex of phenol (**4**) with **3**. (E) Distances or angles that remained constant in each geometry analysis. (F–I) Dependence of the interaction energy on the H···O distance or following angles. (F) H···O distance (*d*_HO_). (G) O-H···O angle (**). (H) H···O=C/H···O-C angle (**). (I) H-elevation angle (*θ*).

**
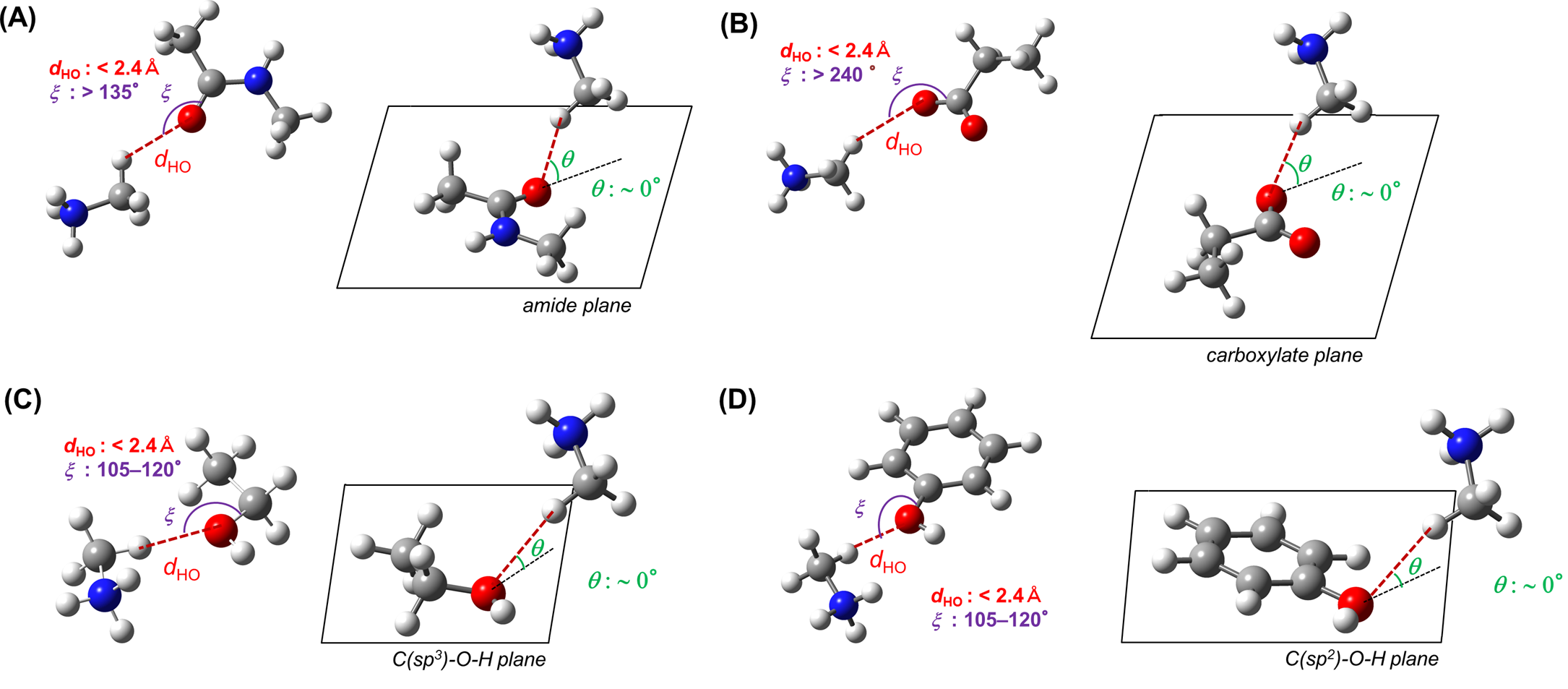
**

**Figure S9.** Interaction models and preferred geometries of complexes of (A) *N*-methylacetamide (**1**)/monomethylamine (**5**) (complex **A4**), (B) propanoate (**2**)/**5** (complex **B4**), (C) ethanol (**3**)/**5** (complex **C4**), and (D) phenol (**4**)/**5** (complex **D4**).

.

**
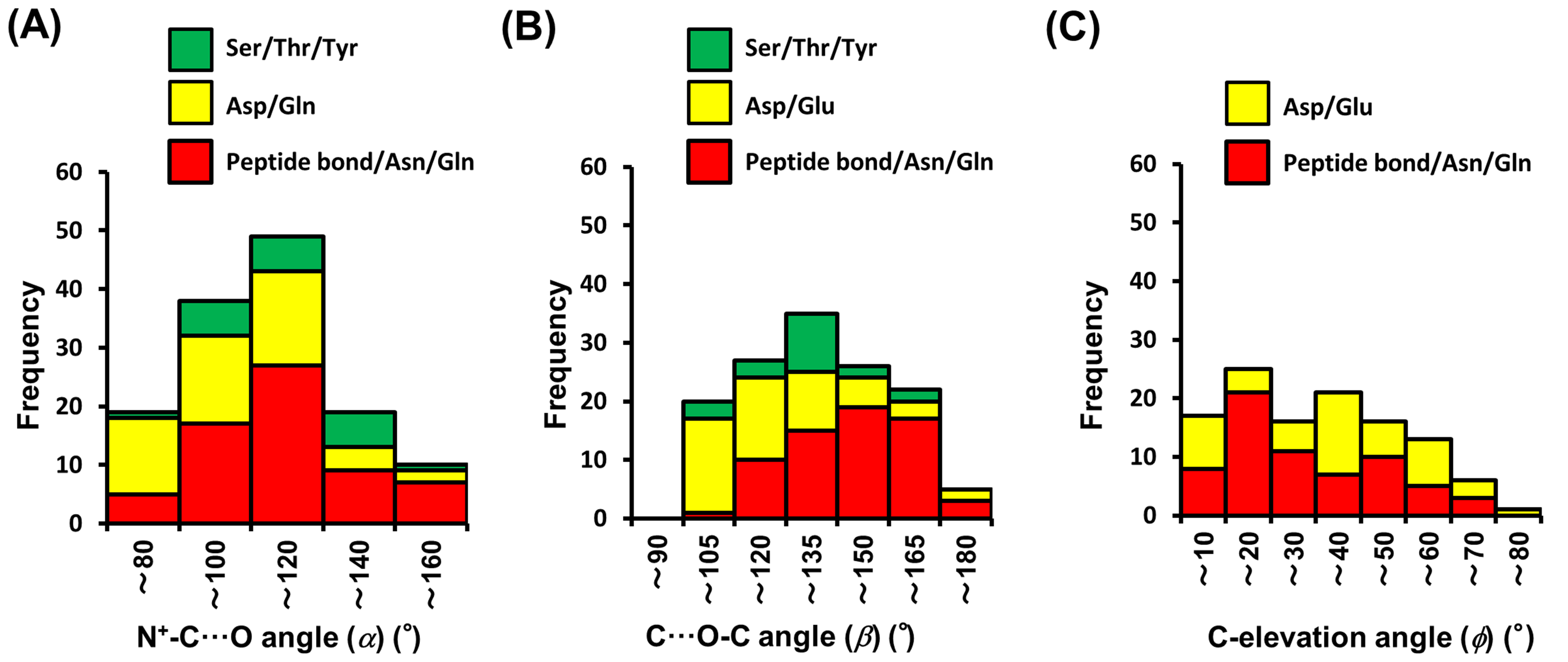
**

**Figure S10.** Histogram for the geometry of N^+^-C-H···O hydrogen bonds with respect to N^+^-C···O angle, C···O-C angle, and C-elevation angle. (A) N^+^-C···O angle (*α*). (B) C···O-C angle (*β*). (C) C-elevation angle (*φ*).


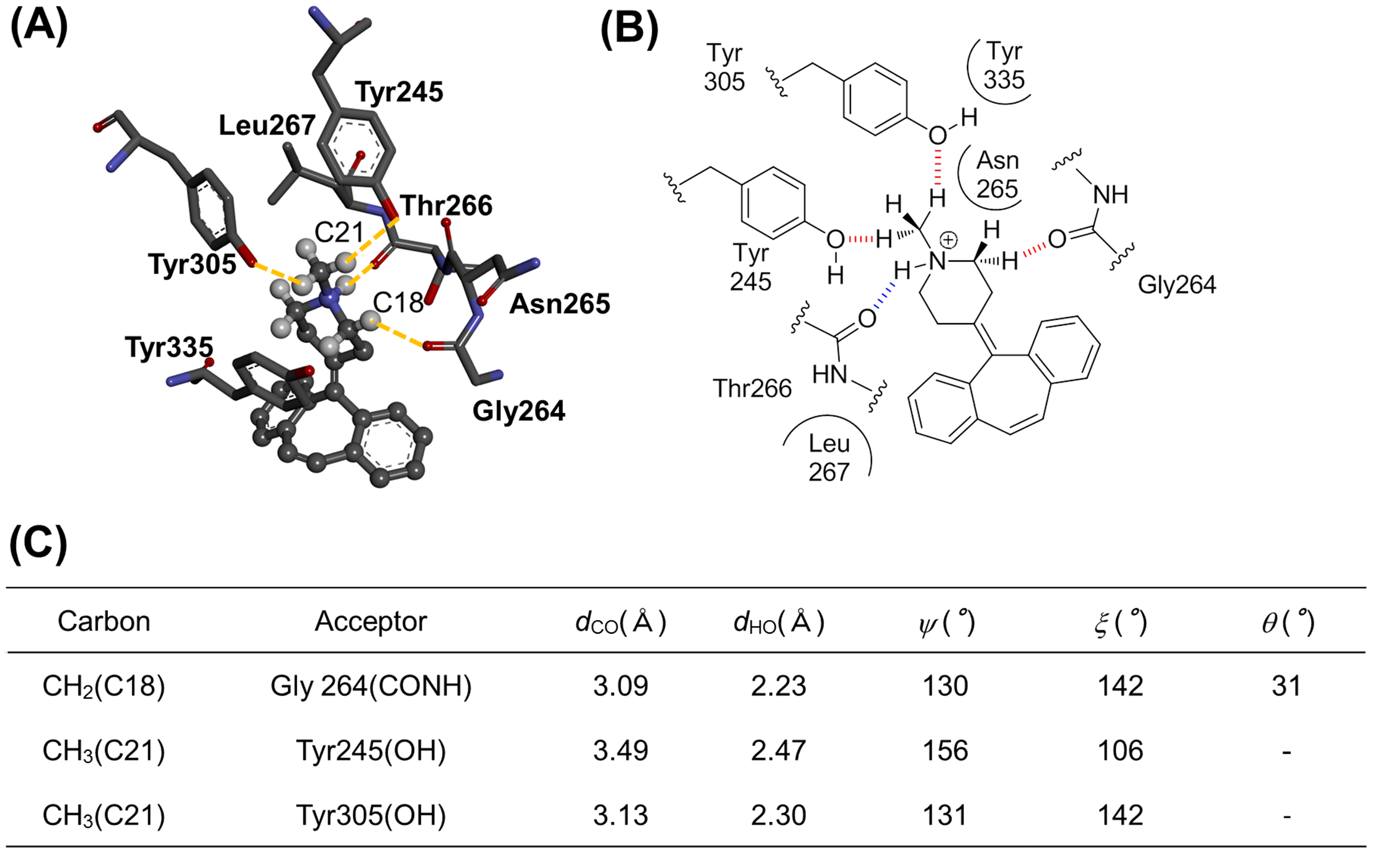


**Figure S11.** Representative examples of N^+^-C-H···O contacts found in the PDB survey. (A) Molecular structure of SET7/9, a histone methyltransferase, complexed with its inhibitor cyproheptadine (PDB ID: 5AYF). This figure shows cyproheptadine (ball-and-stick) and amino acid residues (stick) within a 5 Å radius from the nitrogen atom of cyproheptadine. (B) Schematic diagram for the binding mode of cyproheptadine to SET7/9. The red dotted lines indicate C-H···O hydrogen bonds; the blue dotted line indicates an N-H···O hydrogen bond. (C) Geometry profile for N^+^-C-H···O contacts between SET7/9 and cyproheptidine. The methyl group of cyproheptadine engages in C-H···O hydrogen bonds with Tyr245 and Tyr305, while a methylene group conjugated to the ammonium cation engages in C-H···O hydrogen bonds with the amide oxygen of Gly264. The hydrogen atom of the ammonium cation forms a heteroatom-hydrogen bond with the amide oxygen of Thr266. However, the hydrogen bond should have a large influence on the C-H···O hydrogen bonds (Supplementary Fig. S7).


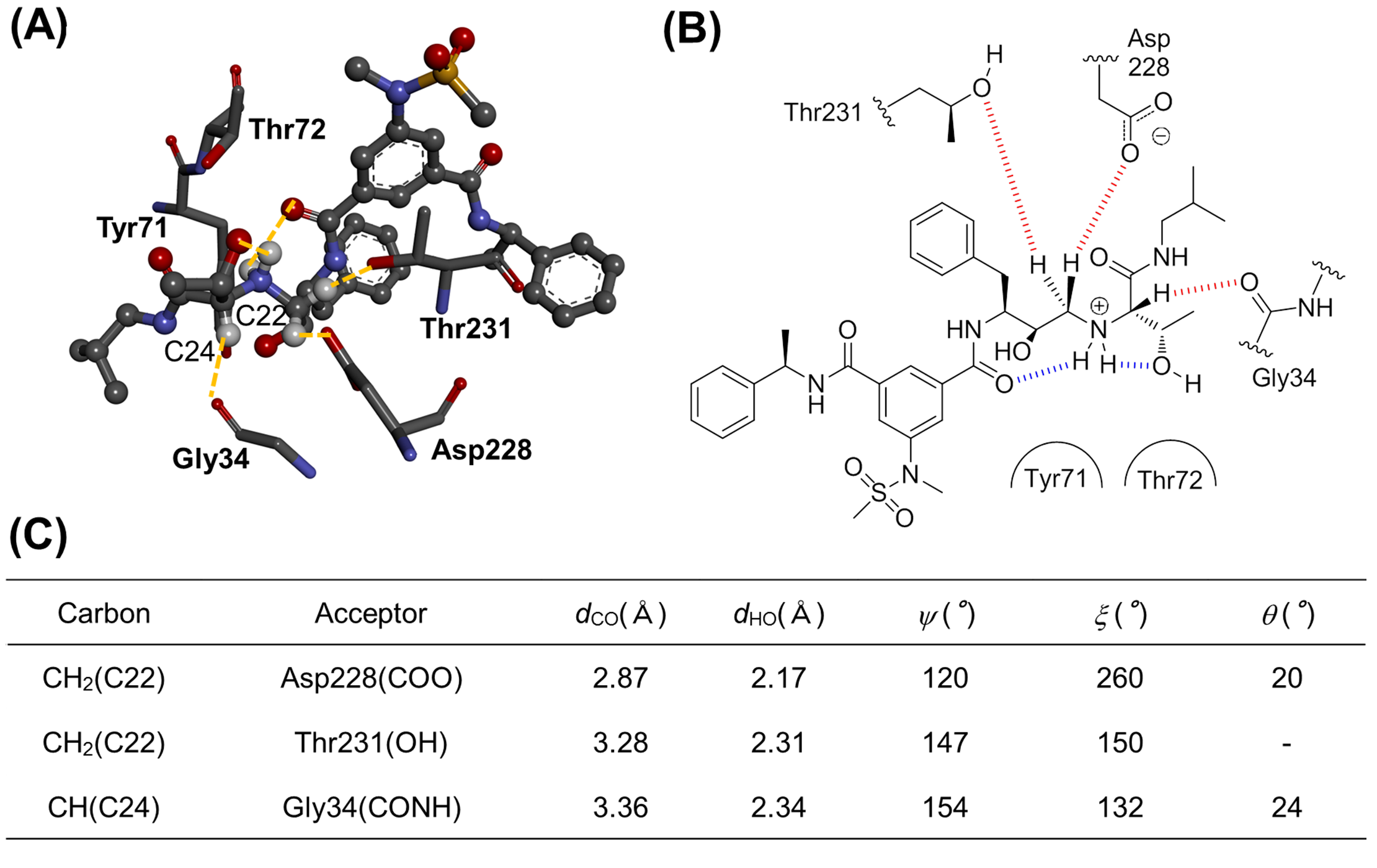


**Figure S12.** Representative examples of N^+^-C-H···O contacts found in the PDB survey. (A) Molecular structure of *β*-secretase-1 (BACE1) complexed with its inhibitor (PDB ID: 5DQC). This figure shows the inhibitor (ball-and-stick) and amino acid residues (stick) within a 5 Å radius from the nitrogen atom of the inhibitor. (B) Schematic diagram for the binding mode of the inhibitor to BACE1. The red dotted lines indicate C-H···O hydrogen bonds; the blue dotted lines indicate N-H···O hydrogen bonds. (C) Geometry profile for the N^+^-C-H···O contacts between BACE1 and its inhibitor. The structure of the *β*-secretase-1/inhibitor complex is stabilized by three C-H···O hydrogen bonds formed between the inhibitor (through a methylene group and a methine group covalently bound to the ammonium cation), and Asp228, Thr231, and Gly34. Both hydrogen atoms of the ammonium cation form intramolecular heteroatom-hydrogen bonds with the oxygen atom of the amide or hydroxyl groups. However, these hydrogen bonds should not have a large influence on the C-H···O hydrogen bonds (Supplementary Fig. S7).

**
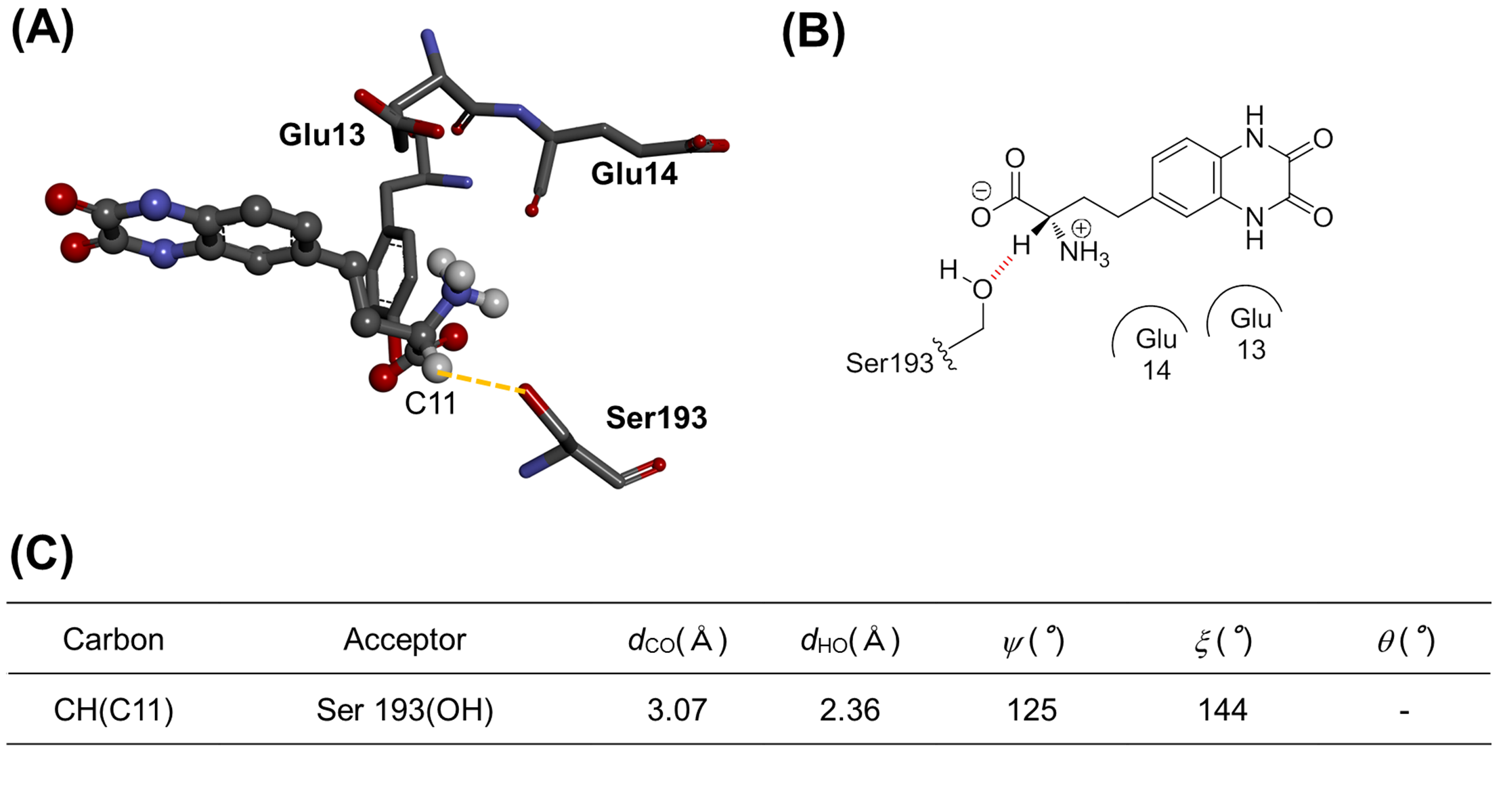
**

**Figure S13.** Representative examples of N^+^-C-H···O contacts found in the PDB survey. (A) Molecular structure of glutamate receptor ionotropic kainite 1 (GluK1) complexed with its ligand (PDB ID: 4QF9). This figure shows the ligand (ball-and-stick) and amino acid residues (stick) within a 5 Å radius from the nitrogen atom of the ligand. (B) Schematic diagram for the binding mode of the ligand to GluK1. The red dotted line indicates a C-H···O hydrogen bond. (C) Geometry profile for the N^+^-C-H···O contacts between GluK1 and its ligand. The complex formed by GluK1 and its ligand is stabilized by one C-H···O hydrogen bond between a methine group that is covalently bound to the ammonium cation in the ligand and Ser193. The three hydrogen atoms of the ammonium cation are removed from any molecules and do not directly interact with any molecules. Even though the ammonium group may be solvated, the solvation should not substantially impact the N^+^-C-H···O hydrogen bond (Supplementary Fig. S4).


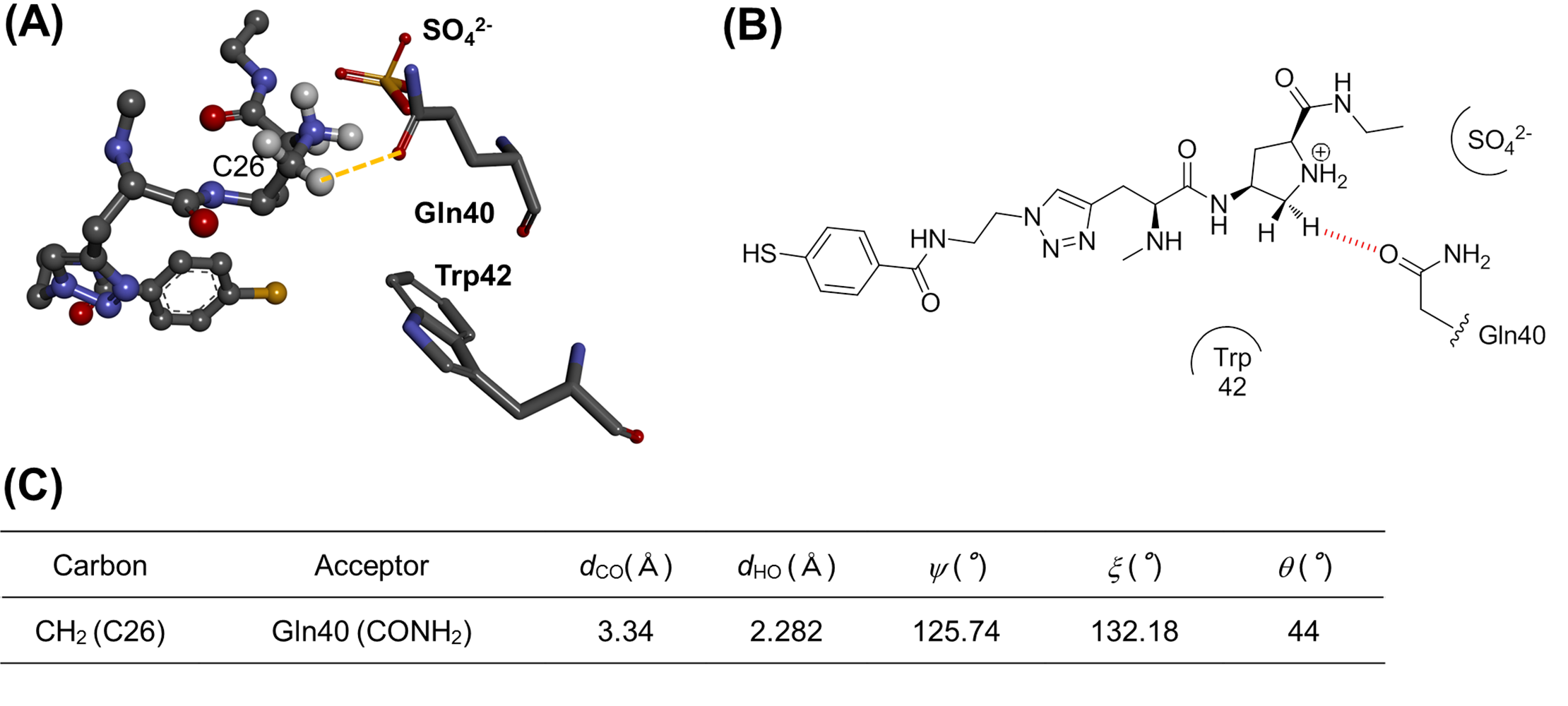


**Figure S14.** Representative examples of N^+^-C-H···O contacts found in the PDB survey. (A) Molecular structure of PA-I galactophilic lectin complexed with its ligand (PDB ID: 4CP9). This figure shows the ligand (ball-and-stick) and amino acid residues/an SO_4_^2-^ molecule (stick) within a 5 Å radius from the nitrogen atom of the ligand. (B) Schematic diagram for the binding mode of the ligand to PA-I galactophilic lectin. The red dotted line indicates C-H···O hydrogen bonds. (C) Geometry profile for the N^+^-C-H···O contacts between PA-I galactophilic lectin and its ligand. The complex formed by PA-I galactophilic lectin and its ligand is stabilized by a C-H···O hydrogen bond between a methylene group that is covalently bound to the ammonium cation in the ligand and Gln40. The two hydrogen atoms of the ammonium cation are removed from any molecules molecule and any molecules including the SO_4_^2-^ anion should not have a large influence on the C-H···O hydrogen bonds (the SO_4_^2-^ molecule is removed from the H atoms by a distance of at least 4.12 Å).


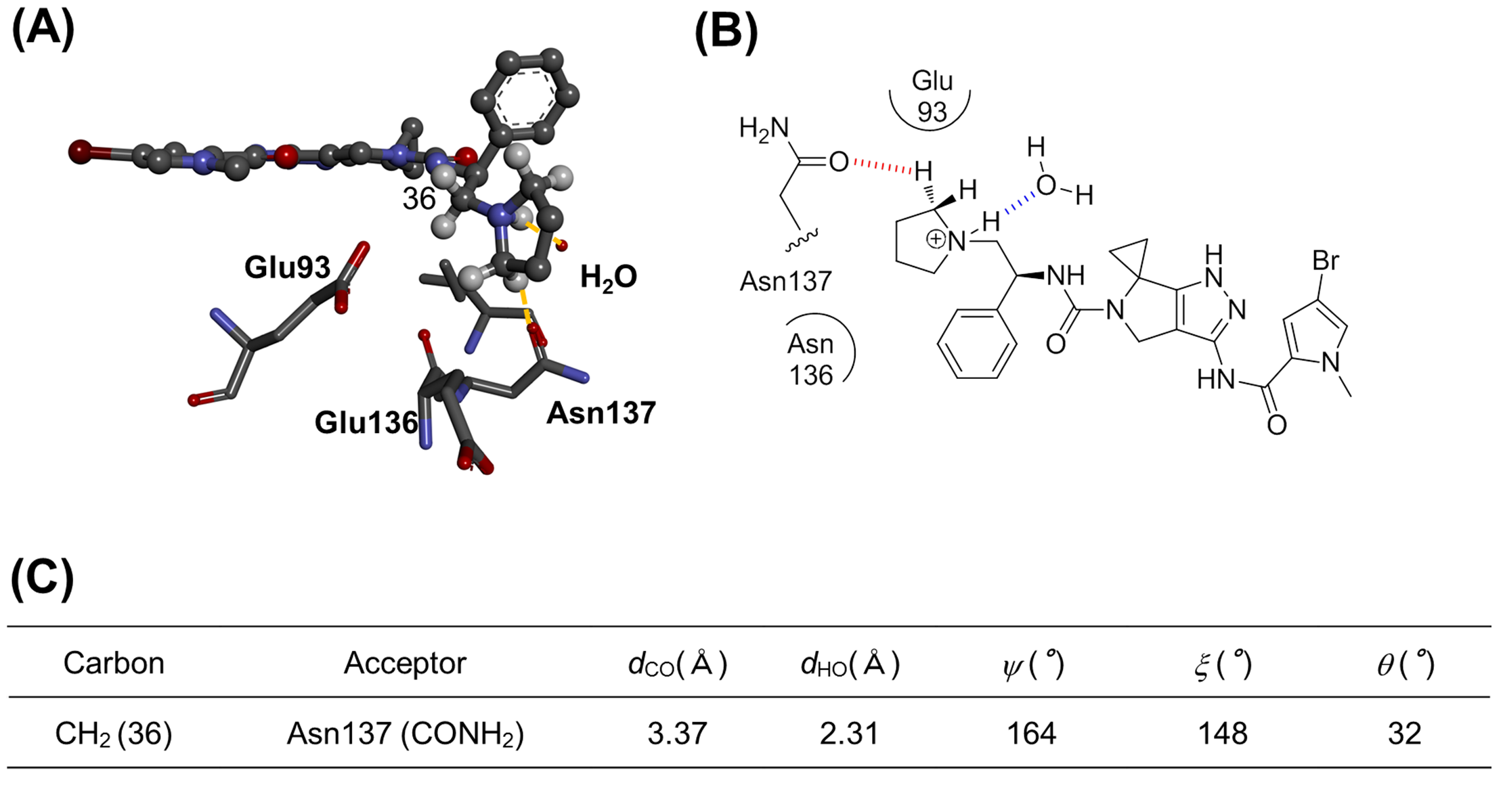


**Figure S15.** Representative examples of N^+^-C-H···O contacts found in the PDB survey. (A) Molecular structure of maternal embryonic leucine zipper kinase (MELK) complexed with its inhibitor (PDB ID: 4BKY). This figure shows the inhibitor (ball-and-stick) and amino acid residues/a water molecule (stick) within a 5 Å radius from the nitrogen atom of the inhibitor. (B) Schematic diagram for the binding mode of the inhibitor to MELK. The red dotted line indicates a C-H···O hydrogen bond; the blue dotted line indicates an N-H···O hydrogen bond. (C) Geometry profile for the N^+^-C-H···O contacts between MELK and its inhibitor. The complex formed by MELK and its inhibitor is stabilized by one C-H···O hydrogen bond between a methylene group that is covalently bound to the ammonium cation in the inhibitor and Asn137. The hydrogen atom of the ammonium cation forms a heteroatom-hydrogen bond with the oxygen atom of the water molecule. However, the hydrogen bond should not have a large influence on the C-H···O hydrogen bonds (Supplementary Fig. S7).


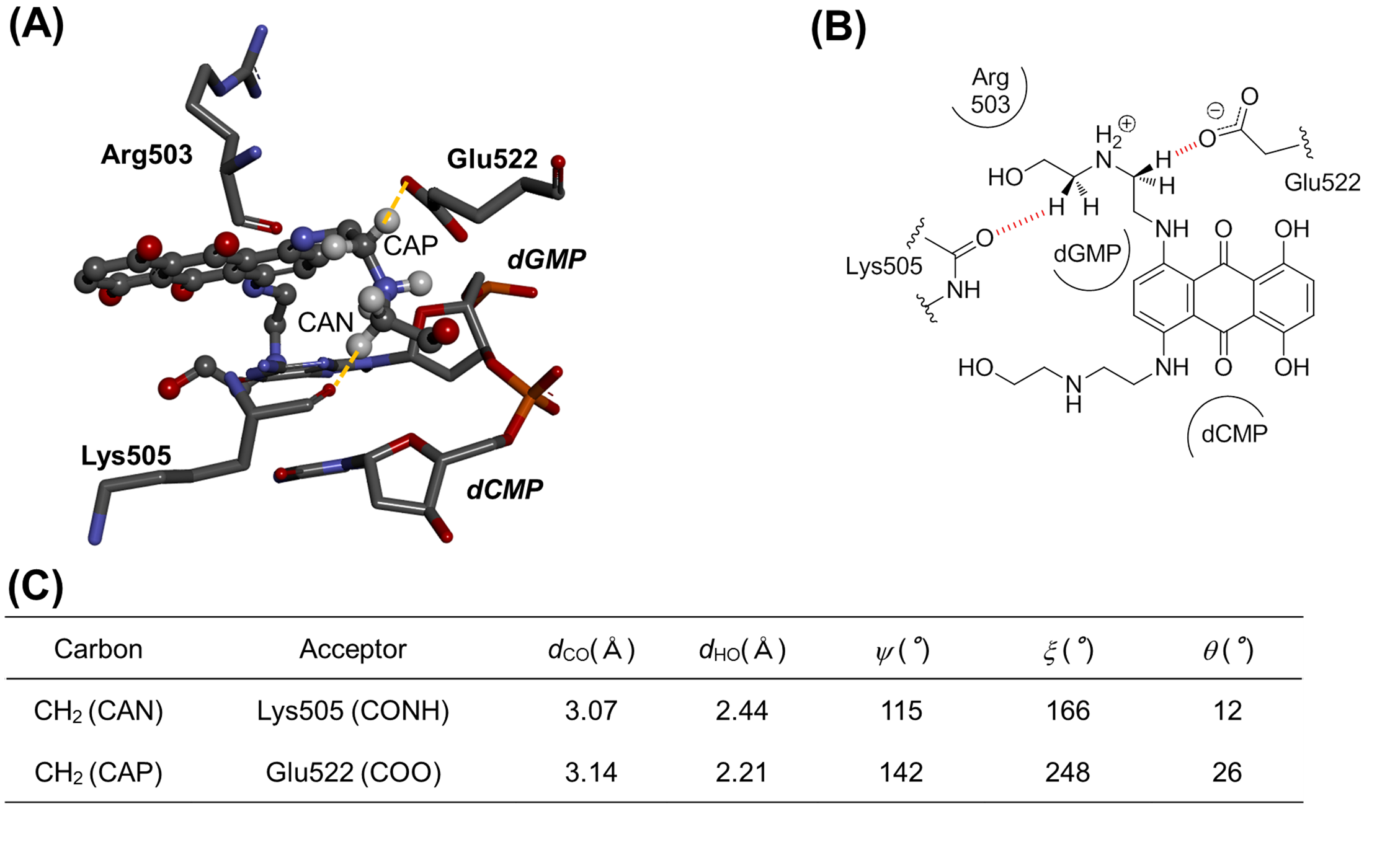


**Figure S16.** Representative examples of N^+^-C-H···O contacts found in the PDB survey. (A) Molecular structure of DNA tomoisomerase 2β complexed with its inhibitor (PDB ID: 4G0V). This figure shows the inhibitor (ball-and-stick) and amino acid residues/deoxyribonucleotides (stick) within a 5 Å radius from the nitrogen atom of the inhibitor; dGMP: deoxyguanosine monophosphate; dCMP: deoxycytidine monophosphate. (B) Schematic diagram for the binding mode of the inhibitor to DNA tomoisomerase 2β. The red dotted lines indicate C-H···O hydrogen bonds. (C) Geometry profile of N^+^-C-H···O contacts between DNA tomoisomerase 2β and its inhibitor. The complex formed by DNA tomoisomerase 2β with its inhibitor is stabilized by two C-H···O hydrogen bonds between the two methylene groups that are covalently bound to the ammonium cation in the inhibitor and the peptide bond of Lys505 and Glu522, respectively. The two hydrogen atoms of the ammonium cation are removed from any molecules and thus do not directly interact with any molecules including the phosphate (the phosphorus atomsare removed from the H atoms by at least 5 Å).


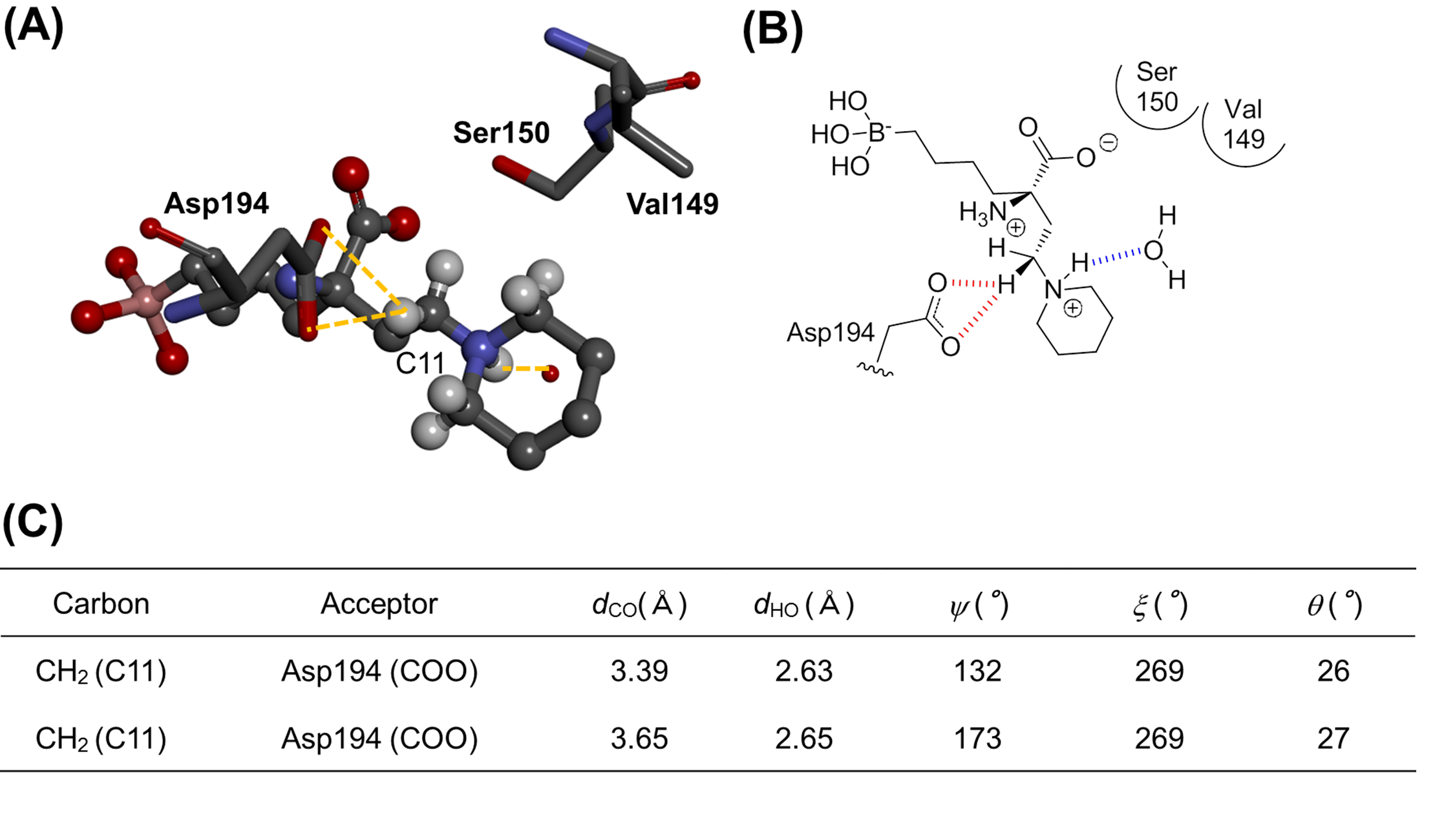


**Figure S17.** Representative examples of N^+^-C-H···O contacts found in the PDB survey. (A) Molecular structure of human arginase-1 complexed with its inhibitor (PDB ID: 5HJ9). This figure shows the inhibitor (ball-and-stick) and amino acid residues/a water molecule (stick) within a 5 Å radius from the nitrogen atom of the inhibitor. (B) Schematic diagram for the binding mode of the inhibitor to human arginase-1. The red dotted lines indicate C-H···O hydrogen bonds; the blue dotted line indicates an N-H···O hydrogen bond. (C) Geometry profile for the N^+^-C-H···O contacts between human arginase-1 and its inhibitor. The complex formed by human arginase-1 and its inhibitor is stabilized by two C-H···O hydrogen bonds between the methylene groups that are covalently bound to the ammonium cation in the inhibitor and the two oxygen atoms in Asp194. The hydrogen atom of the ammonium cation forms a heteroatom-hydrogen bond with the oxygen atom of the water molecule. However, the hydrogen bond should not have a large influence on the C-H···O hydrogen bonds (Supplementary Fig. S7).

**
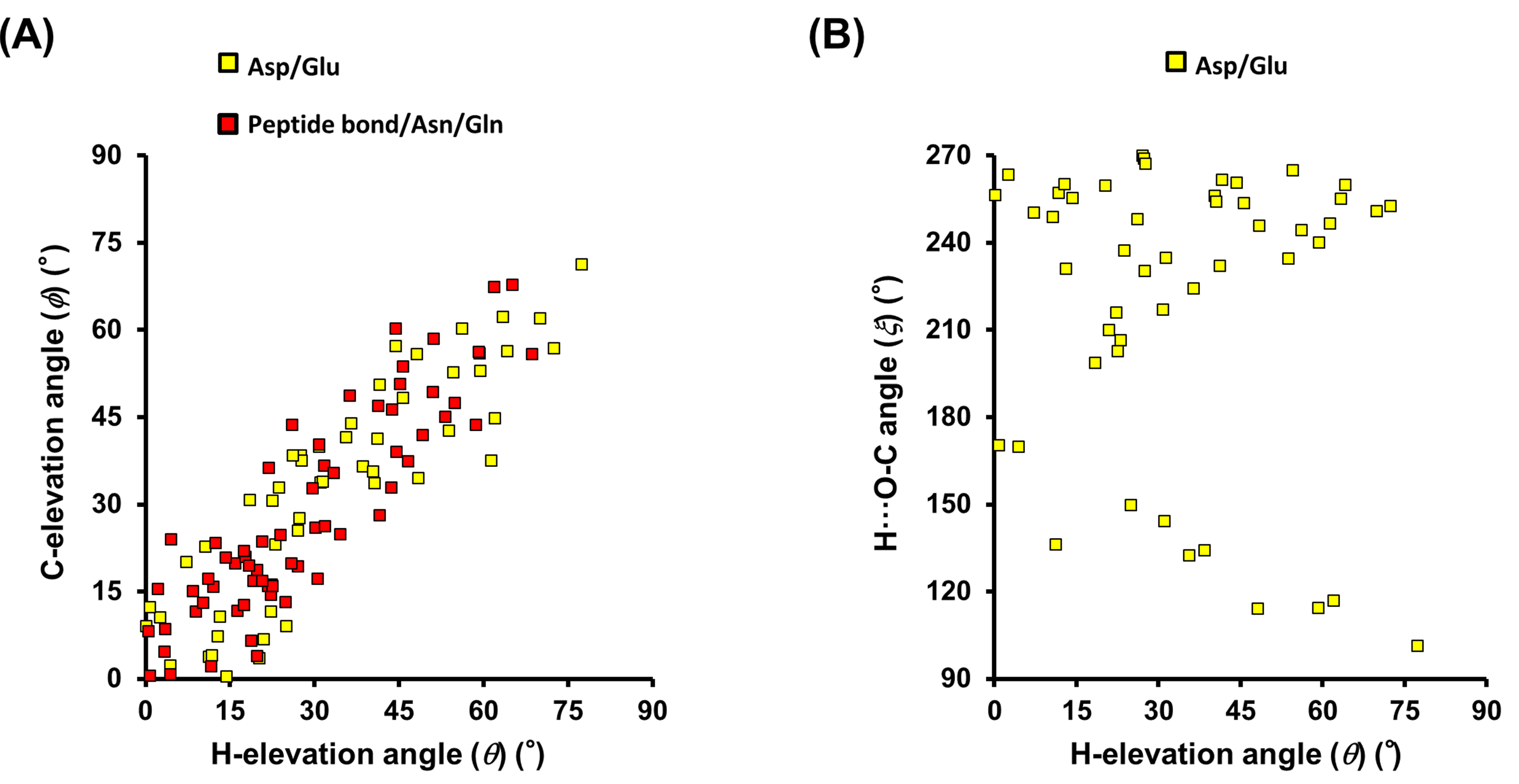
**

**Figure S18.** Correlation between the H-elevation and C-elevation angles, or the H-elevation and H···O-C angles. (A) Correlation between the H-elevation (*θ*) and C-elevation angles (*φ*) using data sets of the peptide bond/Asn/Gln and Asp/Glu acceptors. (B) Correlation between the H-elevation (*θ*)* *and H···O-C angles (**) angles using the data set of Asp/Glu acceptors.

**
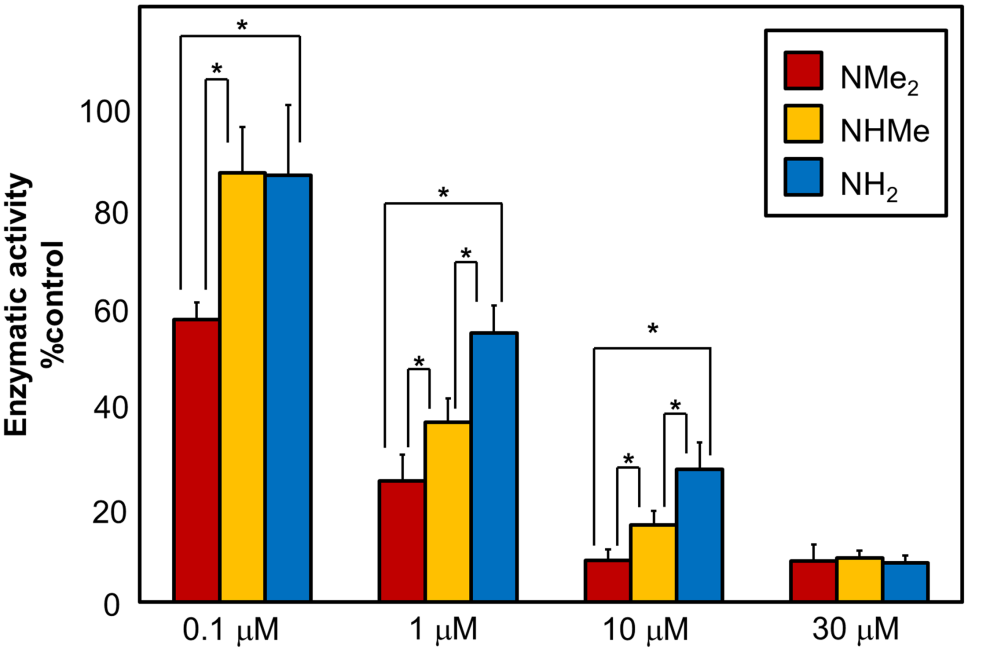
**

**Figure S19.** GLP-inhibitory activity of **14a**–**14d**. ^*^*P* < 0.05; Steel-Dwass tests.


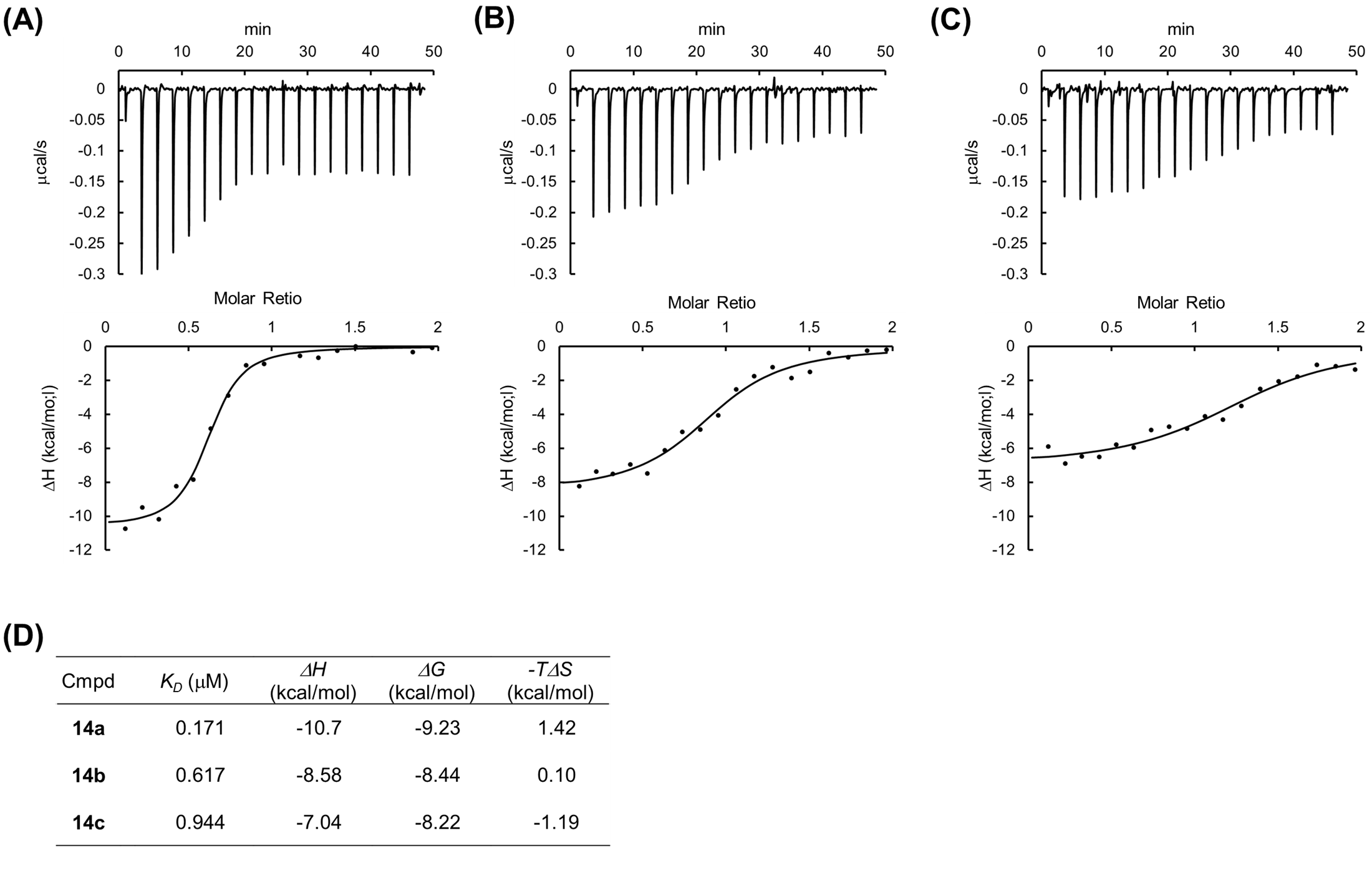


**Figure S20.** Determination of the disassociation constant and thermodynamic parameters of **14a**–**14c** by isothermal titration calorimetry (ITC). (A–C) The binding of (A) **14a**, (B) **14b** and (C) **14c** to GLP are measured by ITC. The titrations were conducted in buffer containing 20 mM Tris pH 8.0, 150 mM NaCl, 2% DMSO and 50 μM AdoMet. The sample chamber and syringe were filled with 10 μM GLP and 100 μM inhibitor, respectively. (D) Summary of disassociation constant and thermodynamic parameter profiles of **14a**–**14c**.

**Synthesis of compounds 14 and 15.**

**General synthetic methods.** NMR spectra were recorded on a BRUKER AVANCE 300 spectrometer (^1^H: 300 MHz, ^13^C: 75 MHz). ^1^H NMR spectra are referenced to the internal standard SiMe_4_ (δ = 0), while ^13^C NMR spectra are expressed relative to the signals of the solvent. High-resolution mass spectra (HRMS) and fast-atom bombardment (FAB) mass spectra were recorded on a JEOL JMS-SX102A mass spectrometer. Electrospray ionization (ESI) mass spectra were recorded on a BRUKER HCTplus mass spectrometer. HPLC analyses and preparative HPLC were performed on an ODS-3 (150 mm x φ4.6 mm, GL Science or Cosmosil) and on an Inertsil ODS-3 (250 mm x φ20 mm, GL Science or Cosmosil), respectively. The HPLC system consisted of a pump (HITACHI, L-6050 intelligent pump) and a detector (HITACHI, L-4000 UV detector). The sample elution required for HPLC was accomplished using linear solvent gradients and the detection wavelength was set to 254 nm. Reagents and solvents were purchased from commercial suppliers (Aldrich, Merck, Nacalai Tesque, Tokyo Kasei Kogyo, Wako Pure Chemical Industries, Kishida Kagaku, and Kanto Kagaku), and used without further purification. DNH PTLC plates were purchased from Fuji Silysia Chemical Ltd. Flash column chromatography was performed using silica gel supplied by TOYOTA SILICA GEL (#AP300D). Compounds **S5**^S1^ and **S6**^S2^ were prepared by previously published methods.

**Scheme S1.** Reaction scheme for the synthesis of **14a**−**d**.

*^a^*Reagents and conditions: (a) *i*-Pr_2_NEt, *N*,*N*-dimethylformamide (DMF), room temperature (51%); (b) amine, 1-butanol, 150 °C (**14a**: 99%, **14d**:88%); (c) HCl, H_2_O, 1,4-dioxane, r.t. (**14b**: 15% over 2 steps, **14c**: 59% over 2 steps).

***N*-(1-Benzylpiperidin-4-yl)-2-chloroquinazolin-4-amine (S3).**

A mixture of 2,4-dichloroquinazoline (**S1**) (2.11 g, 8.15 mmol), 1-benzylpiperidin-4-amine (**S2**) (3.4 mL, 16.6 mmol), and diisopropylethylamine (1.5 mL, 8.70 mmol) in DMF (20 mL) was stirred at room temperature for 3 hours. The reaction was quenched with water and extracted with AcOEt. The organic layer was washed with a 0.5% aq. solution of AcOH, brine, and dried over Na_2_SO_4_. The suspension was filtered and the solvent was removed *in vacuo*. Purification by flash column chromatography (silica gel, CHCl_3_/28% aq. NH_3_/MeOH = 50/3/0 → 2500/150/106) afforded 1.70 g (51%) of **S3** as an orange oil; ^1^H NMR (DMSO-*d_6_*, 300 MHz, δ ppm): 8.13 (1H, *d*, *J* = 7.4 Hz), 7.78 (1H, s), 7.18 (1H, s), 4.28–4.10 (1H, m), 4.00 (3H, s), 4.02 (3H, s), 2.96 (2H, *d*, *J* = 11.5 Hz), 2.32 (3H, s), 2.19–1.95 (4H, m), 1.84–1.71 (2H, m); ^13^C NMR (CDCl_3_, 75 MHz,  ppm) 161.99, 157.90, 157.40, 151.44, 149.27, 139.31, 131.69, 130.21, 129.36, 109.17, 107.45, 103.87, 64.88, 57.69, 54.62, 50.75, 32.98.

***N^4^*-(1-Benzylpiperidin-4-yl)-*N^2^*-[3-(dimethylamino)propyl]quinazoline-2,4-diamine (14a).**

A mixture of *N^1^*,*N^1^*-dimethylpropane-1,3-diamine (0.333 mL, 2.67 mmol) and **S3** (109 mg, 0.264 mmol) in 1-butanol (9.67 mL) was stirred at 150 °C for 3 days, before being cooled to room temperature. The volume of the reaction mixture was reduced *in vacuo*. Purification by flash column chromatography (silica gel, CHCl_3_/28% aqueous NH_3_ solution/MeOH = 10/1/0 → 200/20/11) produced an oil (165 mg). Part of this oil (12.1 mg) was further purified by DNH PTLC (*n*-hexane/2-propanol = 10/3) to afford 9.2 mg (99%) of **14a** as a colorless oil;^1^H NMR (CDCl_3_, 300 MHz,  ppm): 7.36–7.23 (5H, m), 6.88 (1H, s), 6.71 (1H, s), 5.23 (1H, br), 5.07 (1H, d, *J* = 7.6 Hz), 4.18 (1H, m), 3.94 (3H, s), 3.93 (3H, s), 3.55 (2H, s), 3.50 (2H, td, *J* = 6.6, 5.6 Hz), 2.91 (2H, d, *J* = 11.9 Hz), 2.39 (2H, t, *J* = 7.2 Hz), 2.24 (6H, s), 2.22–2.01 (4H, m), 1.79 (2H, quin, *J* = 7.0 Hz), 1.62 (2H, ddd, *J* = 22.8, 11.4, 3.4 Hz); ^13^C NMR (CDCl_3_, 75 MHz,  ppm): 159.02, 158.59, 154.52, 148.30, 145.36, 138.47, 129.08, 128.21, 127.03, 105.32, 103.47, 101.12, 63.10, 57.84, 56.37, 56.01, 52.54, 48.16, 45.61, 40.24, 32.33, 27.95; MS (ESI) *m/z*: 479 (MH^+^); HRMS (FAB): calcd for C_27_H_39_N_6_O_2_^+^, 479.3134; found, 479.3138; HPLC: t*_R_*= 19.50 min, purity 95%; gradient conditions (A, H_2_O containing 0.1% TFA, B, CH_3_CN containing 0.1%TFA): B 5% → B 30% (0 → 20 min), B 30% → 80% (20 → 30 min), B 80% → 100% (30 → 35 min), B 100% (35 → 40 min).

***tert-*Butyl [3-({4-[(1-benzylpiperidin-4-yl)amino]-6,7-dimethoxyquinazolin-2-yl}amino)**

**propyl](methyl)carbamate (S4b).**

A mixture of *tert-*butyl (3-aminopropyl)(methyl)carbamate (433 mg, 2.30 mmol) and **2** (102 mg, 0.247 mmol) in 1-butanol (9 mL) was stirred at 150°C for 1 day, before being cooling to room temperature. The volume of the reaction mixture was reduced *in vacuo* and the residue was purified by PTLC (CHCl_3_/28% aqueous NH_3_ solution/MeOH = 93/7/5) and DNH PTLC (*n*-hexane/2-propanol = 5/1) to obtain crude **S4b** (61.1 mg), which was used in the next step without further purification.

***N*^4^-(1-Benzylpiperidin-4-yl)-6,7-dimethoxy-*N*^2^-[3-(methylamino)propyl]quinazoline**

**-2,4-diamine (14b).**

Crude **S4b** (61.1 mg) was added to a mixture of 8 mL of a 10N aqueous HCl solution and 1,4-dioxane (12 mL) at 0 °C. The resulting mixture was stirred at room temperature for 2 hours and, afterwards, neutralized with a 1M aqueous solution of Na_2_HPO_4_. The organic solvent was removed *in vacuo.* The residue obtained was adsorbed on a short ODS column and subjected to flash chromatography, using MeOH as the eluent. Concentration of the filtrate under reduced pressure afforded an oil that was subsequently purified by DNH PTLC (CHCl_3_/MeOH = 200/1) to furnish **14b** as a colorless oil (16.9 mg, 15% yield over 2 steps); ^1^H NMR (CDCl_3_, 300 MHz,  ppm): 7.34–7.22 (5H, m), 6.86 (1H, s), 6.69 (1H, s), 5.03 (1H, d, *J* = 7.5 Hz), 4.18 (1H, m), 3.96 (3H, s), 3.93 (3H, s), 3.77 (2H, d, *J* = 6.6 Hz), 3.54 (2H, s), 2.96 (3H, s), 2.91 (2H, d, *J* = 12.0 Hz), 2.76 (2H, t, *J* = 6.3 Hz), 2.45–2.07 (6H, m), 1.91–1.79 (2H, m), 1.66 (2H, ddd, *J* = 23.3, 11.7, 3.1 Hz); ^13^C NMR (CDCl_3_, 75 MHz,  ppm): 159.23, 158.26, 154.72, 148.50, 145.43, 138.27, 129.16, 128.26, 127.11, 105.15, 102.74, 101.20, 63.14, 56.51, 56.18, 55.94, 52.63, 48.31, 45.60, 37.78, 34.67, 32.20; MS (ESI) *m/z*: 465 (MH^+^); HRMS (FAB): calcd for C_26_H_37_N_6_O_2_^+^, 465.2978; found, 465.2981; HPLC: t*_R_*= 19.44 min, purity 98%; gradient conditions (A: H_2_O containing 0.1% TFA; B: CH_3_CN containing 0.1%TFA): B 5% → B 30% (0 → 20 min), B 30% → 80% (20 → 30 min), B 80% → 100% (30 → 35 min), B 100% (35 → 40 min).

***tert*-Butyl [3-({4-[(1-benzylpiperidin-4-yl)amino]quinazolin-2-yl}amino)propyl]carbamate (S4c).**

A mixture of *tert*-butyl (3-aminopropyl)carbamate (436 mg, 2.50 mmol) and **S3** (103 mg, 0.249 mmol) in 1-butanol (9 mL) was stirred at 150 °C for 2 days. The reaction mixture was allowed to cool to room temperature, before being concentrated *in vacuo.* The thus obtained residue was purified by flash column chromatography (silica gel, CHCl_3_/28% aq. NH_3_ /MeOH = 250/7/0 → 12500/350/257) to afford an oil (154 mg). Part of this oil (71.1 mg) was further purified by DNH PTLC (hexane/2-propanol = 10/3), and the thus obtained crude **S4c** (70.5 mg) was used in the next reaction step without further purification.

***N^2^*-(3-Aminopropyl)-*N^4^*-(1-benzylpiperidin-4-yl)quinazoline-2,4-diamine (14c).**

Crude **S4c** (obtained as described above) (43.7 mg) was dissolved in 10N aq. HCl/1,4-dioxane (8/12 mL) at 0 °C. The resulting mixture was stirred at room temperature for 2 hours. The reaction mixture was adjusted to pH = 7 using a 1M aqueous solution of Na_2_HPO_4_, before the organic solvent was removed under reduced pressure. The obtained residue was purified by flash chromatography on a short ODS column using MeOH as the eluent, and the obtained organic fractions were concentrated *in vacuo*. The residue was purified by DNH PTLC (CHCl_3_/MeOH =100/1) to give **14c** as a colorless oil (18.9 mg, 59% over 2 steps); ^1^H NMR (CDCl_3_, 300 MHz,  ppm): 7.35–7.22 (5H, m), 6.86 (1H, s), 6.73 (1H, s), 5.12 (1H, d, *J* = 7.5 Hz), 4.97 (1H, br), 4.18 (1H, m), 3.94 (3H, s), 3.92 (3H, s), 3.54 (4H, overlap), 2.91 (2H, d, *J* = 11.8 Hz), 2.82 (2H, t, *J* = 6.7 Hz), 2.26–2.05 (4H, m), 1.79 (2H, quin, *J* = 6.7 Hz), 1.63 (2H, ddd, *J* = 23.0, 11.5, 3.4 Hz); ^13^C NMR (CDCl_3_, 75 MHz,  ppm): 159.12, 158.61, 154.65, 147.90, 145.55, 138.35, 129.13, 128.24, 127.08, 105.14, 103.56, 101.23, 63.11, 56.47, 56.09, 52.55, 48.05, 39.23, 38.80, 33.15, 32.30; MS (ESI) *m/z*: 451 (MH^+^); HRMS (FAB): calcd for C_25_H_34_N_6_O_2_^+^, 451.2821; found, 451.2827; HPLC: t*_R_*= 19.19 min, purity 98%; gradient conditions (A: H_2_O containing 0.1% TFA; B: CH_3_CN containing 0.1%TFA): B 5% → B 30% (0 → 20 min), B 30% → 80% (20 → 30 min), B 80% → 100% (30 → 35 min), B 100% (35 → 40 min).

***N^4^*-(1-Benzylpiperidin-4-yl)-*N^2^*-(4-methylpentyl)quinazoline-2,4-diamine (14d).**

A solution of 4-methylpentanenitrile (5 mL, 41.2 mmol) in Et_2_O (10 mL) was slowly added to a suspension of LiAlH_4_ (1.62 g, 42.7 mmol) in THF (27 mL) at 0 °C. The reaction mixture was stirred at room temperature for 2.5 hours, before being quenched with water and extracted with Et_2_O. The organic layer was washed with brine and dried over Na_2_SO_4_. Filtration and evaporation *in vacuo* afforded 3.41 g of crude 4-methylpentan-1-amine as a colorless oil, which was used in the next reaction without further purification. A mixture of the crude 4-methylpentan-1-amine mentioned above (743 mg) and**S3** (100 mg, 0.242 mmol) in 1-butanol (9 mL) was stirred at 150 °C for 2 days, before being allowed to cool to room temperature. Concentration of the reaction mixture, followed by purification of the thus obtained residue by PTLC (CHCl_3_/28% aq. NH_3_ /MeOH = 550/50/33) furnished crude **14d** (189 mg). Crude **14d** (15.2 mg) was purified by DNH PTLC (hexane/2-propanol = 5/1) to give a colorless oil (8.2 mg, 88% from **S3**); ^1^H NMR (CDCl_3_, 300 MHz,  ppm): 7.35–7.23 (5H, m), 6.87 (1H, s), 6.70 (1H, s), 5.04 (1H, d, *J* = 7.2 Hz), 4.18 (1H, m), 3.95 (3H, s), 3.93 (3H, s), 3.55 (2H, s), 3.43 (2H, q, *J* = 6.7 Hz), 2.91 (2H, d, *J* = 11.9 Hz), 2.26–2.08 (4H, m), 1.68–1.50 (4H, m), 1.33–1.19 (3H, m), 0.90 (6H, d, *J* = 6.6 Hz); ^13^C NMR (CDCl_3_, 75 MHz,  ppm): 159.16, 158.56, 154.53, 148.57, 145.33, 138.48, 129.10, 128.22, 127.04, 105.49, 103.47, 101.01, 63.12, 56.37, 56.01, 52.55, 48.11, 41.88, 36.32, 32.38, 28.01, 27.90, 22.64; MS (ESI) *m/z*: 478 (MH^+^); HRMS (FAB) calcd for C_28_H_40_N_5_O_2_^+^, 478.3182; found, 478.3176; HPLC: t*_R_*= 20.40 min, purity 98%; gradient conditions (A: H_2_O containing 0.1% TFA; B: CH_3_CN containing 0.1%TFA): B 5% → B 30% (0 → 20 min), B 30% → 80% (20 → 30 min), B 80% → 100% (30 → 35 min), B 100% (35 → 40 min).

**Scheme S2.** Reaction scheme for the synthesis of **15a**−**d**.*^a^*

*^a^*Reagents and conditions: (a) Pd(OAc)_2_, 2,2'-bis(diphenylphosphino)-1,1'-binaphthyl (BINAP), CsCO_3_, toluene, 80 °C (87%); (b) NaOH, THF, MeOH, H_2_O, room temperature (96%); (c) (Boc)_2_O, Et_3_N, *N*,*N*-dimethylaminopyridine (DMAP), CH_2_Cl_2_, room temperature (96%); (d) NaH, MeI, DMF, 50 °C (76%); (e) THF, reflux; (f) Pd(dppf)Cl_2_, K_2_CO_3_, DMF, H_2_O, 90–100 °C (**S13b**: 84%; **S13c**: 99%; **S14d** from **S12**: 88%); (g) LiAlH_4_, THF, reflux (46%); (h) 1-Ethyl-3-(3-dimethylaminopropyl)carbodiimide (EDCI), DMAP, DMF, 70–85 °C (**15a**: 45%; **S15b**: 70%; **S15c**: 66%; **15d**: 66%); (i) HCl, 1,4-dioxane, MeOH, room temperature (**15b**: 77%; **15c**: 95%).

**Methyl 4-methyl-3-{[3-(pyrimidin-4-yl)pyridin-2-yl]amino}benzoate (S7).**

A mixture of 4-(2-chloropyridin-3-yl)pyrimidine (**S5**)^32^ (56.3 mg, 0.294 mmol), 3-amino-4-methylbenzoate (**S6**)^33^ (63.3 mg, 0.383 mmol), Pd(OAc)_2_ (6.91 mg, 0.0308 mmol), BINAP (24.7 mg, 0.0397 mmol), and CsCO_3_ (1137 mg, 5.89 mmol) in toluene (5.00 mL) was deoxygenated for 5 minutes using a stream of Argon. The reaction mixture was stirred for 13 hours at 80 °C. After cooling the reaction mixture to room temperature, a standard extraction protocol using AcOEt, water, and brine was applied. The organic layer was dried over Na_2_SO_4,_ filtered, concentrated under reduced pressure, and purified by flash column chromatography (silica gel, AcOEt/*n*-hexane = 1/2 → 1/1), which delivered **S7** as a yellow solid (82.4 mg, 87%); ^1^H NMR (CDCl_3_, 300 MHz,  ppm): 11.50 (1H, br), 9.27 (1H, d, *J* = 1.2 Hz), 8.82 (1H, d, *J* = 2.4 Hz), 8.81 (1H, d, *J* = 1.2 Hz), 8.39 (1H, dd, *J* = 4.8, 1.8 Hz), 8.11 (1H, dd, *J* = 8.0, 2.0 Hz), 7.82 (1H, dd, *J* = 5.7, 1.5 Hz), 7.70 (1H, dd, *J* = 7.8, 1.8 Hz), 7.30 (1H, d, *J* = 7.8 Hz), 6.86 (1H, dd, *J* = 7.8, 4.8 Hz), 3.91 (3H, s), 2.48 (3H, s); ^13^C NMR (CDCl_3_, 75 MHz,  ppm): 167.40, 163.45, 157.31, 157.03, 138.88, 137.18, 134.71, 130.45, 128.53, 124.26, 123.14, 117.47, 114.03, 113.30, 51.98, 19.12.

**4-Methyl-3-{[3-(pyrimidin-4-yl)pyridin-2-yl]amino}benzoic acid (S8).**

An aqueous solution of NaOH (1N, 3.00 mL, 3.00 mmol) was added to a solution of **S7** (79.4 mg, 0.248 mmol) in THF/MeOH (3.00 mL/3.00 mL), and the mixture was stirred at room temperature for 14 hours. The precipitation of a yellow solid was induced by neutralization with 1N HCl (3.00 mL). The solid was washed with water and dried *in vacuo* to yield **S8** (72.9 mg, 96%); ^1^H NMR (DMSO, 300 MHz,  ppm): 12.72 (1H, br), 11.73 (1H, br), 9.36 (1H, d, *J* = 1.2 Hz), 8.92 (1H, d, *J* = 5.1 Hz), 8.88 (1H, d, *J* = 1.5 Hz), 8.49 (1H, dd, *J* = 8.1, 1.8 Hz), 8.37 (1H, dd, *J* = 4.05 1.8 Hz), 8.25 (1H, dd, *J* = 5.6, 1.2 Hz), 7.53 (1H, dd, *J* = 7.8, 1.8 Hz), 7.35 (1H, d, *J* = 8.1 Hz), 6.99 (1H, dd, *J* = 7.8, 4.8 Hz), 2.46 (3H, s); ^13^C NMR (DMSO, 75 MHz,  ppm): 169.47, 164.24, 163.76, 159.80, 155.75, 140.89, 140.17, 135.05, 132.17, 130.74, 124.99, 123.59, 120.11, 116.51, 115.32, 20.68.

***tert*-Butyl allylcarbamate (S10c).**

A solution of (Boc)_2_O (4810 mg, 22.04 mmol) in CH_2_Cl_2_ (50 mL) was added to a solution of allylamine (**S9c**)(2326 mg, 40.74 mmol), Et_3_N (10.00 mL, 72.14 mmol), and DMAP (270 mg, 2.210 mmol) in CH_2_Cl_2_ (50 mL). The resulting mixture was stirred at room temperature for 3 hours, before being poured into water. Then, a standard extraction and washing protocol was applied, followed by further purification of the thus obtained oil by flash column chromatography (silica gel, AcOEt/*n*-hexane = 1/6), which delivered **S10c** as a yellow solid (3326 mg, 96%); ^1^H NMR (CDCl_3_, 300 MHz,  ppm): 5.85 (1H, ddt, *J* = 17.1, 10.2, 5.4 Hz), 5.18 (1H, ddt, *J* = 17.1, 1.6 Hz), 5.12 (1H, ddt, *J* = 10.2, 1.6, 1.6 Hz), 4.59 (1H, br), 3.75 (2H, br), 1.45 (9H, s); ^13^C NMR (CDCl_3_, 75 MHz,  ppm): 155.78, 134.94, 115.70, 79.40, 43.12, 28.41.

***tert*-Butyl allyl(methyl)carbamate (S10b).**

A solution of *tert*-butyl allylcarbamate (**S10c**) (997 mg, 6.34 mmol) in DMF (2.5 mL) was added to a suspension of 60% NaH in oil (603 mg, 15.08 mmol) in DMF (2.5 mL) at 0 °C. Then, MeI (1.50 mL, 24.09 mmol) was added to the mixture in a dropwise fashion, and the resulting mixture was heated to 50 °C for 6 hours. Then, the reaction mixture was poured into water and extracted with AcOEt. The organic layer was washed with brine and dried over Na_2_SO_4_. After filtration, concentration under reduced pressure, and purification by flash column chromatography (silica gel, AcOEt/*n*-hexane = 1/9), **S10b** was obtained as a colorless oil (823 mg, 76%); ^1^H NMR (CDCl_3_, 300 MHz,  ppm): 5.77 (1H, ddt, *J* = 15.4, 11.0, 5.7 Hz), 5.14–5.06 (2H, m), 3.81 (2H, br), 2.82 (3H, s), 1.46 (9H, s); ^13^C NMR (CDCl_3_, 75 MHz,  ppm): 155.75, 133.72, 116.30, 79.40, 51.40, 33.74, 28.44.

***tert*-Butyl {3-[3-amino-5-(trifluoromethyl)phenyl]propyl}(methyl)carbamate (S13b).**

0.5 N 9-BBN in THF (15.00 mL, 2.50 mmol) was added to *tert*-butyl allyl(methyl)carbamate (**S10b**) (806 mg, 4.71 mmol). The resulting mixture was heated to reflux for 7 hours. Then, the reaction was concentrated *in vacuo* to afford crude **S11b**, which was used for the next step without further purification. Pd(dppf)Cl_2_/CH_2_Cl_2_ (53.7 mg, 0.0658 mmol) and **S11b** were sequentially added to a degassed mixture of 3-amino-5-bromo-benzotrifluoride (**S12**) (339 mg, 1.41 mmol) and K_2_CO_3_ (813 mg, 5.88 mmol) in DMF/H_2_O (10.00 mL/1.00 mL). The resulting mixture was heated to 90 °C for 12 hours. The reaction mixture was allowed to cool to room temperature, before being poured into H_2_O. The mixture was extracted with AcOEt, and the organic fraction was washed with 2 N NaOH and brine, before being dried over Na_2_SO_4_. Filtration and concentration *in vacuo* delivered a residue that was purified by flash column chromatography (silica gel, AcOEt/*n*-hexane = 1/4 → 1/3), which afforded **S13b** as a colorless oil (393 mg, 84%); ^1^H NMR (CDCl_3_ 300 MHz,  ppm): 6.81 (1H, s), 6.73 (1H, s), 6.65 (1H, s), 3.80 (2H, br), 3.24 (2H, br), 2.84 (3H, s), 2.54 (2H, quin, *J* = 7.9 Hz), 1.82 (2H, quin, *J* = 7.9 Hz), 1.44 (9H, s); ^13^C NMR (CDCl_3_, 75 MHz,  ppm): 155.85, 146.92, 143.95, 131.68 (q, *J_cf_* = 31.2 Hz), 124. 72 (q, *J_cf_* = 270.8 Hz), 117.92, 114.93 (q, *J_cf_* = 3.8 Hz), 109.08 (q, *J_cf_* = 3.8 Hz), 79.33, 48.42, 34.17, 33.00, 29.18, 28.46; MS (ESI) *m/z*: 233 (MH^+^-Boc).

**3-[3-(Dimethylamino)propyl]-5-(trifluoromethyl)aniline (S13a).**

A solution of *tert*-butyl {3-[3-amino-5-(trifluoromethyl)phenyl]propyl}(methyl)carbamate (**S13b**) (123 mg, 0.370 mmol) in THF (2.5 mL) was added to a suspension of LiAlH_4_ (32.1 mg, 0.846 mmol) in THF (2.5 mL) at 0 °C in a dropwise fashion, and the resulting mixture was heated to reflux and stirred for 7 hours. Water (0.800 mL) and a 15% aqueous solution of NaOH were added consecutively to the reaction mixture. This slurry was filtered, and the thus obtained solid was discharged after having being washed with THF. The THF fractions were added to the filtrate, and this solution was concentrated in *vacuo*. The thus obtained residue was purified by flash column chromatography (DNH silica gel, AcOEt/*n*-hexane = 1/3 → 1/1) to afford **S13a** as a brown oil (42.3 mg, 46%); ^1^H NMR (CDCl_3_, 300 MHz, , ppm): 6.75 (1H, s), 6.65 (1H, s), 6.58 (1H, s), 3.73 (2H, br), 2.51 (2H, d, *J* = 7.8 Hz), 2.23 (2H, t, *J* = 7.4 Hz), 2.17 (6H, s), 1.70 (2H, q, *J* = 7.6 Hz); ^13^C NMR (CDCl_3_, 75 MHz,  ppm): 146.26, 143.76, 130.65 (q, *J_CF_* = 31.5 Hz), 123.74 (q, *J_CF_* = 270.8 Hz), 117.49, 114.67 (q, *J_CF_* = 3.8 Hz), 108.57 (q, *J_CF_* = 3.8 Hz), 58.44, 44.79, 32.88,28.36; MS (ESI) *m/z*: 247 (MH^+^).

***tert*-Butyl {3-[3-amino-5-(trifluoromethyl)phenyl]propyl}carbamate (S13c).**

**S13c** was prepared from **S10c** and **S12** using a similar procedure as described for the preparation of **S13b**; colorless oil (453 mg, 99%); ^1^H NMR (CDCl_3_ 300 MHz,  ppm): 6.81 (1H, s), 6.73 (1H, s), 6.65 (1H, s), 3.80 (2H, br), 3.24 (2H, br), 2.84 (3H, s), 2.54 (2H, quin, *J* = 7.9 Hz), 1.82 (2H, quin, *J* = 7.9 Hz), 1.44 (9H, s); ^13^C NMR (CDCl_3_, 75 MHz,  ppm): 155.85, 146.92, 143.95, 131.68 (q, *J_CF_* = 31.2 Hz), 124. 72 (q, *J_CF_* = 270.8 Hz), 117.92, 114.93 (q, *J_CF_* = 3.8 Hz), 109.08 (q, *J_CF_* = 3.8 Hz), 79.33, 48.42, 34.17, 33.00, 29.18, 28.46; MS (ESI) *m/z*: 233 (MH^+^-Boc).

**3-(4-Methylpentyl)-5-(trifluoromethyl)aniline (S13d).**

**S13d** was prepared from **S10d** and **S12** using a similar procedure to that described for the preparation of **S13b**; colorless oil(272 mg, 88%); ^1^H NMR (CDCl_3_ 300 MHz,  ppm): 6.81 (1H, s), 6.71 (1H, s), 6.64 (1H, s), 3.76 (2H, br), 2.53 (2H, t, *J* = 7.8 Hz), 1.62–1.50 (3H, m), 1.21 (2H, q, *J* = 7.0 Hz), 0.88 (6H, d, *J* = 6.6 Hz); ^13^C NMR (CDCl_3_, 75 MHz,  ppm) 146.64, 145.08, 131.51 (q, *J_CF_* = 31.3 Hz), 124.32 (q, *J_CF_* = 270.8 Hz), 118.05, 115.28 (q, *J_CF_* = 3.8 Hz), 108.96 (q, *J_CF_* = 3.8 Hz), 38.60, 36.10, 29.00, 27.88, 22.57; MS (ESI) *m/z*: 246 (MH^+^).

***N*-{3-[3-(Dimethylamino)propyl]-5-(trifluoromethyl)phenyl}-4-methyl-3-{[3-(pyrimidin-4-yl)pyridin-2-yl]amino}benzamide (15a).**

A mixture of 4-methyl-3-{[3-(pyrimidin-4-yl)pyridin-2-yl]amino}benzoic acid (**S8**) (31.6 mg, 0.103 mmol), 3-[3-(dimethylamino)propyl]-5-(trifluoromethyl)aniline (**S13a**) (42.0 mg, 0.126 mmol), EDCI (61.1 mg, 0.319 mmol), and DMAP (59.3 mg, 0.485 mmol) in DMF (2 mL) was heated to 70 °C for 7 hours. The reaction mixture was allowed to cool to room temperature, before being poured into water. This mixture was extracted with AcOEt, and the organic layer was washed with 2N NaOH and brine, before being dried over Na_2_SO_4_. After filtration and removal of all volatiles under reduced pressure, the thus obtained residue was purified by flash column chromatography (DNH silica gel, AcOEt/*n*-hexane = 1/2 → AcOEt) to afford **15a** as a yellow solid (24.9 mg, 45%); ^1^H NMR (CDCl_3_ 300 MHz,  ppm): 11.62 (1H, br), 9.24 (1H, br), 8.80 (1H, d, *J* = 5.7 Hz), 8.75 (1H, d, *J* = 3.5 Hz), 8.48 (1H, br), 8.35 (1H, dd, *J* = 7.5, 1.5 Hz), 8.10 (1H, dd, *J* = 7.8, 1.8 Hz), 7.81–7.76 (2H, m), 7.74 (1H, s), 7.50 (1H, dd, *J* = 7.8, 1.8 Hz), 7.30 (1H, d, *J* = 8.1 Hz), 7.20 (1H, s), 6.85 (1H, dd, *J* = 7.8, 4.8 Hz), 2.69 (2H, t, *J* = 7.8 Hz), 2.47 (3H, s), 2.30 (2H, t, *J* = 7.4 Hz), 2.23 (6H, s), 1.81 (2H, q, *J* = 7.6 Hz); ^13^C NMR (CDCl_3_, 75 MHz,  ppm): 166.36, 163.20, 157.40, 156.04, 154.54, 151.06, 144.23, 139.10, 139.01, 137.43, 133.02, 132.89, 131.27 (q, *J_CF_* = 31.5 Hz), 130.86, 124.48 (q, *J_CF_* = 270.8 Hz), 123.10, 121.62, 120.73 (q, *J_CF_* = 3.8 Hz), 119.82, 117.51, 114.47 (q, *J_CF_* = 3.8 Hz), 114.31, 113.64, 38.58, 36.20, 29.10, 27.88, 22.58, 19.04; MS (ESI) *m/z*: 535 (MH^+^); HPLC: t*_R_*= 16.64 min, purity 96%; gradient conditions (A: H_2_O containing 0.1% TFA; B: CH_3_CN containing 0.1%TFA): B 5% → B 35% (0 → 10 min), B 35% → 80% (10 → 20 min), B 80% → 95% (20 → 25 min), B 95% (25 → 40 min).

***tert-*Butyl methyl{3-[3-(4-methyl-3-{[3-(pyrimidin-4-yl)pyridin-2-yl]amino}benzamido)-5-**

**(trifluoromethyl)phenyl]propyl}carbamate (S14b).**

**S14b** was prepared from **S8** and **S13b** using a procedure similar to that described for the preparation of **15a**; yellow solid (83.8 mg, 70%); ^1^H NMR (CDCl_3_ 300 MHz,  ppm): 11.61 (1H, br), 9.25 (1H, d, *J* = 1.2 Hz), 8.81 (1H, s), 8.80 (1H, d, *J* = 5.6 Hz), 8.39 (1H, br), 8.36 (1H, dd, *J* = 4.8, 1.8 Hz), 8.10 (1H, dd, *J* = 7.9, 1.8 Hz), 7.79 (1H, dd, *J* = 5.7, 1.3 Hz), 7.76 (1H, br), 7.49 (1H, dd, *J* = 7.8, 1.8 Hz), 7.29 (1H, d, *J* = 7.9 Hz), 7.18 (1H, s), 6.87 (1H, dd, *J* = 7.8, 4.8 Hz), 3.26 (2H, m), 2.84 (3H, s), 2.67 (2H, t, *J* = 7.9 Hz), 2.46 (3H, s), 1.86 (2H, quin, *J* = 7.6 Hz), 1.44 (9H, s); ^13^C NMR (CDCl_3_, 75 MHz,  ppm): 166.31, 163.21, 157.40, 156.95, 155.83, 154.55, 151.09, 143.08, 139.18, 139.09, 137.39, 132.91, 131.34 (q, *J_CF_* = 31.5 Hz), 130.84, 124.00 (q, *J_CF_* = 270.8 Hz), 123.03, 121.52, 120.52 (q, *J_CF_* = 3.8 Hz), 119.85, 117.50, 114.53 (q, *J_CF_* = 3.8 Hz), 114.32, 113.62, 79.38, 48.29,34.19, 33.04, 29.17, 28.47, 19.00; MS (ESI) *m/z*: 621 (MH^+^).

**4-Methyl-*N*-{3-[3-(methylamino)propyl]-5-(trifluoromethyl)phenyl}-3-{[3-(pyrimidin-4-yl)pyridin-2-yl]**

**amino}benzamide (15b).**

A 4N solution of HCl in dioxane (2 mL) was added to a solution of **S14b** (66.5 mg, 0.107 mmol) in MeOH (0.50 mL), which was cooled in an ice-bath. The resulting mixture was stirred at room temperature for 4 hours. The reaction mixture was then adjusted to pH = 7 using a 1N aqueous solution of NaOH. Then, the reaction mixture was extracted with AcOEt, and the organic layer was washed with brine and dried over Na_2_SO_4_. Filtration and evaporation *in vacuo*, followed by purification via PTLC (DNH silica gel, AcOEt) afforded **15a** as a yellow amorphous solid (53.1 mg, 95%); ^1^H NMR (CDCl_3_, 300 MHz,  ppm): 11.66 (1H, s), 9.29 (1H, d, *J* = 5.4 Hz), 8.83 (1H, d, *J* = 5.4 Hz), 8.81 (1H, s), 8.41 (1H, s), 8.40 (1H, dd, *J* = 4.8, 1.8 Hz), 8.1４ (1H, dd, *J* = 7.8, 1.8 Hz), 7.84–7.75 (3H, m), 7.52 (1H, dd, *J* = 7.8, 1.8 Hz), 7.33 (1H, dd, *J* = 5.1, 2.4 Hz), 6.89 (1H, dd, *J* = 7.8, 4.8 Hz), 2.74 (2H, t, *J* = 7.7 Hz), 2.63 (2H, t, *J* = 7.2 Hz), 2.52 (3H, s), 2.45 (3H, s), 1.87 (2H, q, *J* = 7.5 Hz); ^13^C NMR (CDCl_3_, 75 MHz,  ppm): 166.33, 163.21, 157.40, 156.95, 154.57, 151.08, 144.22, 139.20, 139.03, 137.36, 132.95, 132.88, 131.28 (q, *J_CF_* = 31.5 Hz), 130.82, 124.04 (q, *J_CF_* = 270.8 Hz), 123.12, 121.53, 120.65 (q, *J_CF_* = 3.8 Hz), 119.86, 117.49, 114.47 (q, *J_CF_* = 3.8 Hz), 114.32, 113.62, 51.39, 36.44, 33.52, 31.20. 18.99; MS (ESI) *m/z*: 534 (MH^+^); HPLC: *t_R_*= 16.42 min, purity 98%; gradient conditions (A: H_2_O containing 0.1% TFA; B: CH_3_CN containing 0.1%TFA): B 5% → B 35% (0 → 10 min), B 35% → 80% (10 → 20 min), B 80% → 95% (20 → 25 min), B 95% (25 → 40 min).

***tert*-Butyl　{3-[3-(4-methyl-3-{[3-(pyrimidin-4-yl)pyridin-2-yl]amino}benzamido)-5-(trifluoromethyl)**

**phenyl]propyl}carbamate (S14c).**

**S14c** was prepared from **S8** and **S13c** using a procedure similar to that described for the preparation of **15a**;yellow solid (81.9 mg, 66%); ^1^H NMR (CDCl_3_ 300 MHz,  ppm): 11.61 (1H, s), 9.24 (1H, d, *J* = 1.2 Hz), 8.80 (1H, d, *J* = 3.4 Hz), 8.79 (1H, s), 8.48 (1H, br), 8.35 (1H, dd, *J* = 4.8, 1.8 Hz), 8.10 (1H, dd, *J* = 7.9, 1.8 Hz), 7.85–7.74 (2H, m), 7.49 (1H, dd, *J* = 7.9, 1.8 Hz), 7.27 (1H, d, *J* = 7.8 Hz), 7.17 (1H, s), 6.85 (1H, dd, *J* = 7.8, 4.8 Hz), 4.66 (1H, br), 3.14 (2H, q, *J* = 6.3 Hz), 2.68 (2H, t, *J* = 7.8 Hz), 2.45 (3H, s), 1.79 (2H, quin, *J* = 7.4 Hz), 1.44 (9H, s); ^13^C NMR (CDCl_3_, 75 MHz,  ppm): 166.38, 163.20, 157.38, 156.94, 156.06, 154.54, 151.07, 143.55, 139.13, 137.40, 132.93, 132.90, 131.30 (q, *J_CF_* = 31.5 Hz), 130.81, 124.00 (q, *J_CF_* = 270.8 Hz), 123.11, 121.58, 120.58 (q, *J_CF_* = 3.8 Hz), 119.89, 117.50, 114.62, (q, *J_CF_* = 3.8 Hz), 114.31, 113.62, 79.28, 40.11, 32.98, 31.47, 28.42, 18.98; MS (ESI) *m/z:* 607 (MH^+^).

***N*-[3-(3-Aminopropyl)-5-(trifluoromethyl)phenyl]-4-methyl-3-{[3-(pyrimidin-4-yl)pyridin-2-yl]amino}**

**benzamide (15c).**

**15c** was prepared from **S14c** using a procedure similar to that described for **15b**;yellow solid (36.4 mg, 77%); ^1^H NMR (CDCl_3_, 300 MHz,  ppm): 11.66 (1H, s), 9.29 (1H, s), 8.85–8.81 (2H, m), 8.41 (1H, m), 8.16–8.12 (2H, m), 7.84–7.78 (3H, m), 7.50 (1H, d, *J* = 7.5 Hz), 7.35 (1H, d, *J* = 6.6 Hz), 7.21 (1H, s), 6.92–6.87 (1H, m), 2.78–2.70 (4H, m), 2.52 (3H, s), 1.81 (2H, q, *J* = 6.9 Hz); ^13^C NMR (CDCl_3_, 75 MHz,  ppm): 166.69, 163.50, 157.24, 154.87, 151.35, 144.46, 139.48,139.39, 137.26, 133.26, 133.17, 131.56 (q, *J_CF_* = 31.5 Hz), 131.11, 124.33 (q, *J_CF_* = 270.8 Hz), 123.48, 121.85, 120.92 (q, *J_CF_* = 3.8 Hz), 120.19, 117.79, 114.78 (q, *J_CF_* = 3.8 Hz), 114.61, 113.91, 41.92, 35.28, 33.48, 19.27; MS (ESI) *m/z*: 534 (MH^+^); HPLC: *t_R_*= 16.18 min, purity 98%; gradient conditions (A: H_2_O containing 0.1% TFA; B: CH_3_CN containing 0.1%TFA): B 5% → B 35% (0 → 10 min), B 35% → 80% (10 → 20 min), B 80% → 95% (20 → 25 min), B 95% (25 → 40 min).

**4-Methyl-*N*-[3-(4-methylpentyl)-5-(trifluoromethyl)phenyl]-3-{[3-(pyrimidin-4-yl)pyridin-2-**

**yl]amino}benzamide (15d).**

**15d** was prepared from **S8** and **S13d** using a procedure similar to that described forthe preparation of **15a**; yellow solid (97.1 mg, 78%); ^1^H NMR (CDCl_3_ 300 MHz,  ppm): 11.65 (1H, br), 9.28 (1H, d, *J* = 1.3 Hz), 8.84 (1H, s), 8.83 (1H, d, *J* = 4.7 Hz), 8.40 (1H, dd, *J* = 5.7, 1.8 Hz), 8.13 (1H, dd, *J* = 7.9, 1.8 Hz), 8.05 (1H, s), 7.82 (1H, dd, *J* = 5.7, 1.3 Hz), 7.50 (1H, dd, *J* = 7.8, 1.9 Hz), 7.34 (1H, d, *J* = 8.0 Hz), 7.20 (1H, s), 6.89 (1H, dd, *J* = 7.8, 4.8 Hz), 2.65 (2H, t, *J* = 7.8 Hz), 2.51 (3H, s), 1.68–1.50 (3H, m), 0.88 (6H, d, *J* = 6.6 Hz); ^13^C NMR (CDCl_3_, 75 MHz,  ppm): 166.07, 163.29, 157.44, 157.01, 154.62, 151.20, 145.04, 139.36, 138.71, 137.34, 132.99, 132.86, 131.26 (q, *J_CF_* = 31.5 Hz), 130.89, 124.05 (q, *J_CF_* = 270.8 Hz), 123.07, 121.33, 120.81 (q, *J_CF_* = 3.8 Hz), 119.78, 117.54, 114.35, 114.23 (q, *J_CF_* = 3.8 Hz), 113.64, 38.58, 36.20, 29.10, 27.88, 22.58, 19.04; MS (ESI) *m/z*: 534 (MH^+^); HPLC: *t_R_*= 25.11 min, purity 97%; gradient conditions (A: H_2_O containing 0.1% TFA; B: CH_3_CN containing 0.1%TFA): B 5% → B 35% (0 → 10 min), B 35% → 80% (10 → 20 min), B 80% → 95% (20 → 25 min), B 95% (25 → 40 min).

**^1^H NMR (Compound 14a)**


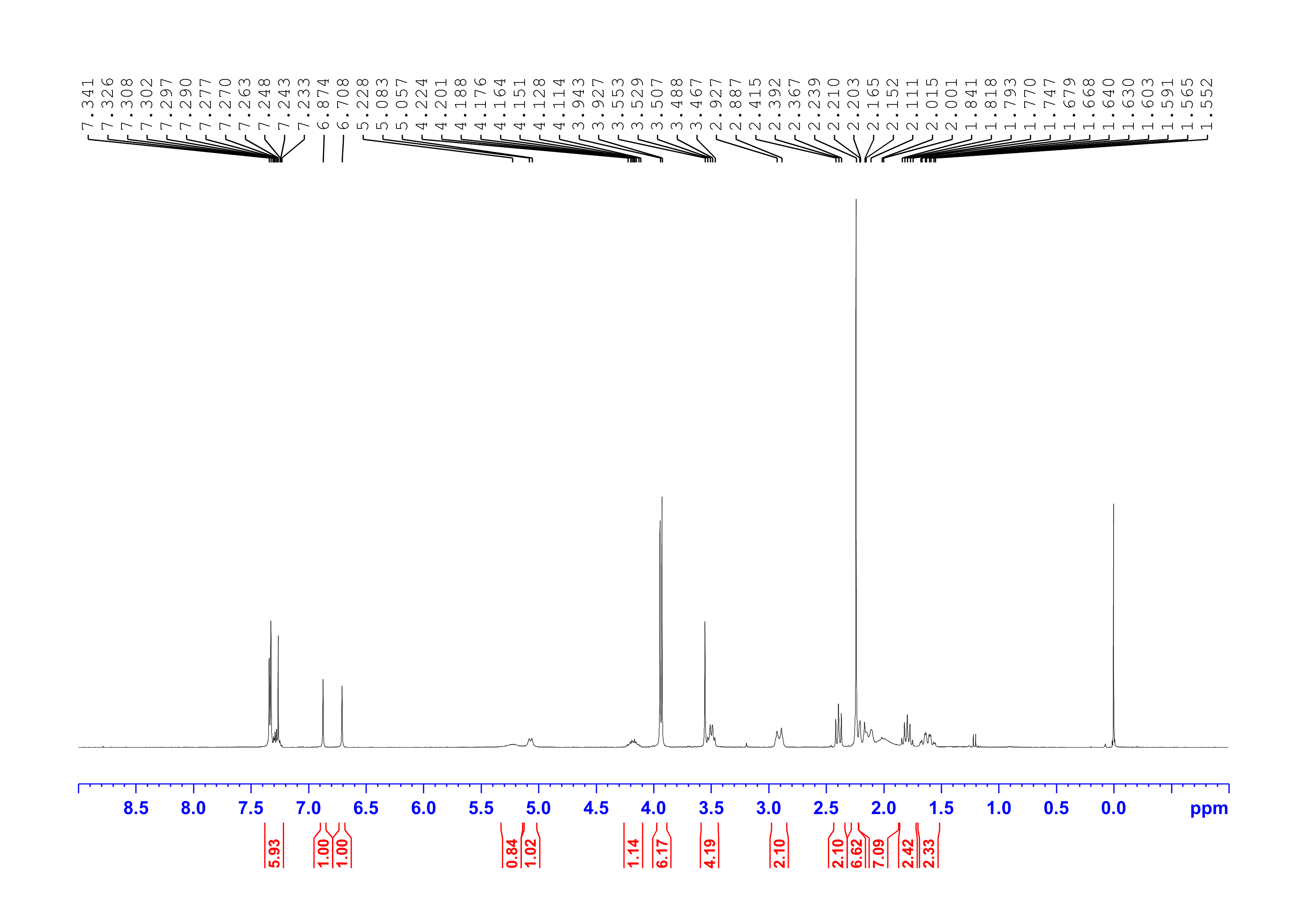


**^13^C NMR (Compound 14a)**

**
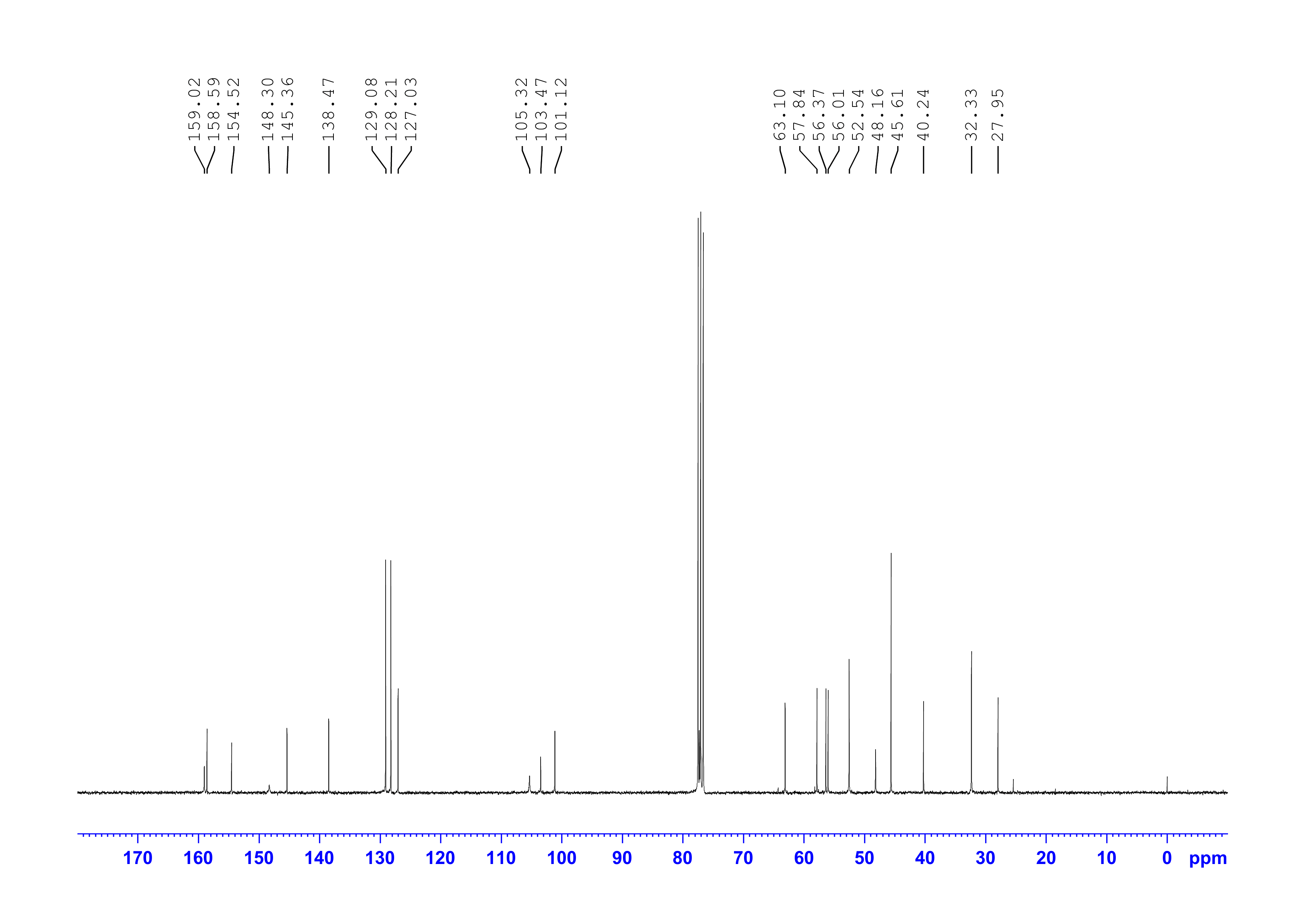
**

**^1^H NMR (Compound 14b)**

**
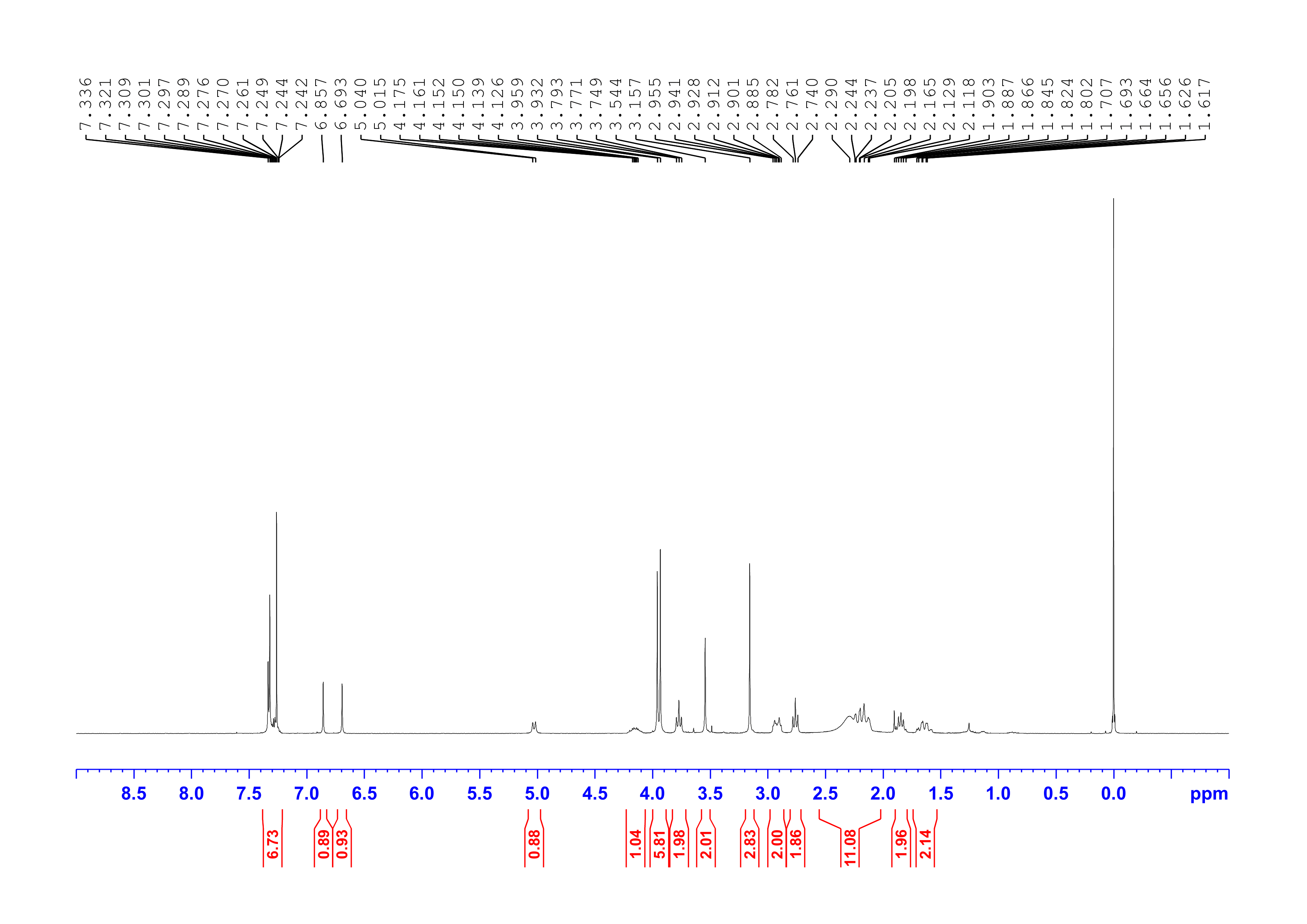
**

**^13^C NMR (Compound 14b)**

**
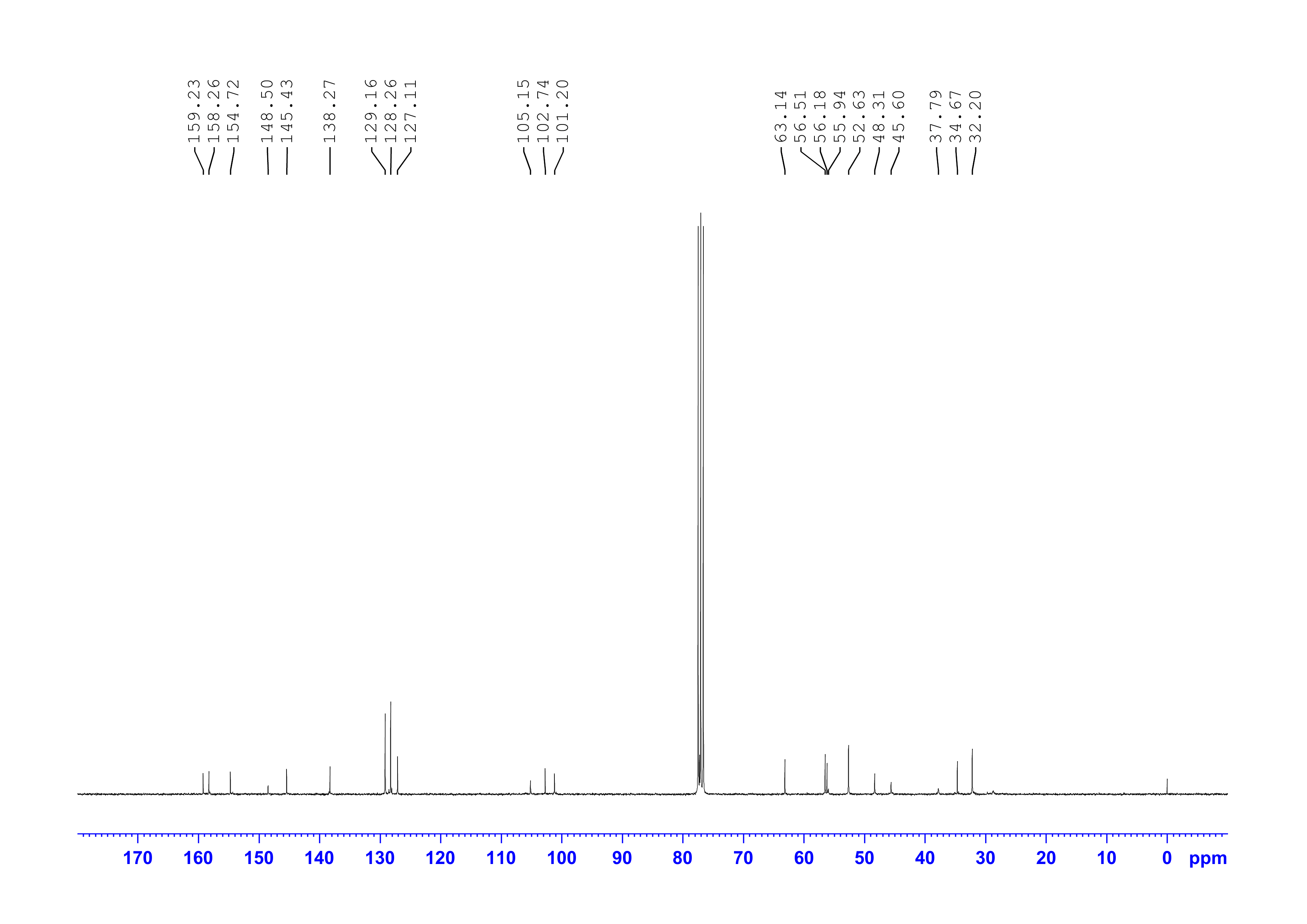
**

**^1^H NMR (Compound 14c)**

**
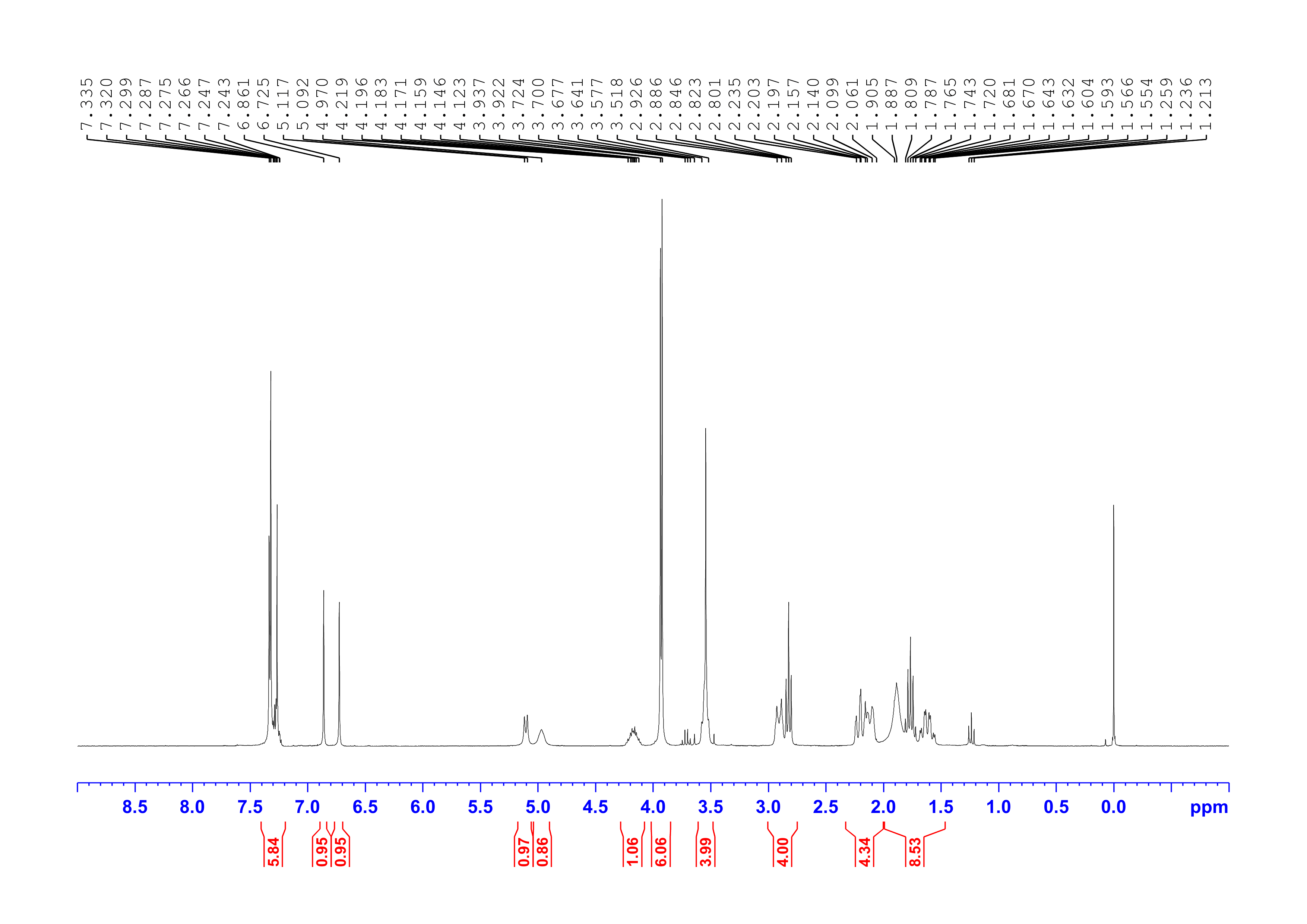
**

**^13^C NMR (Compound 14c)**

**
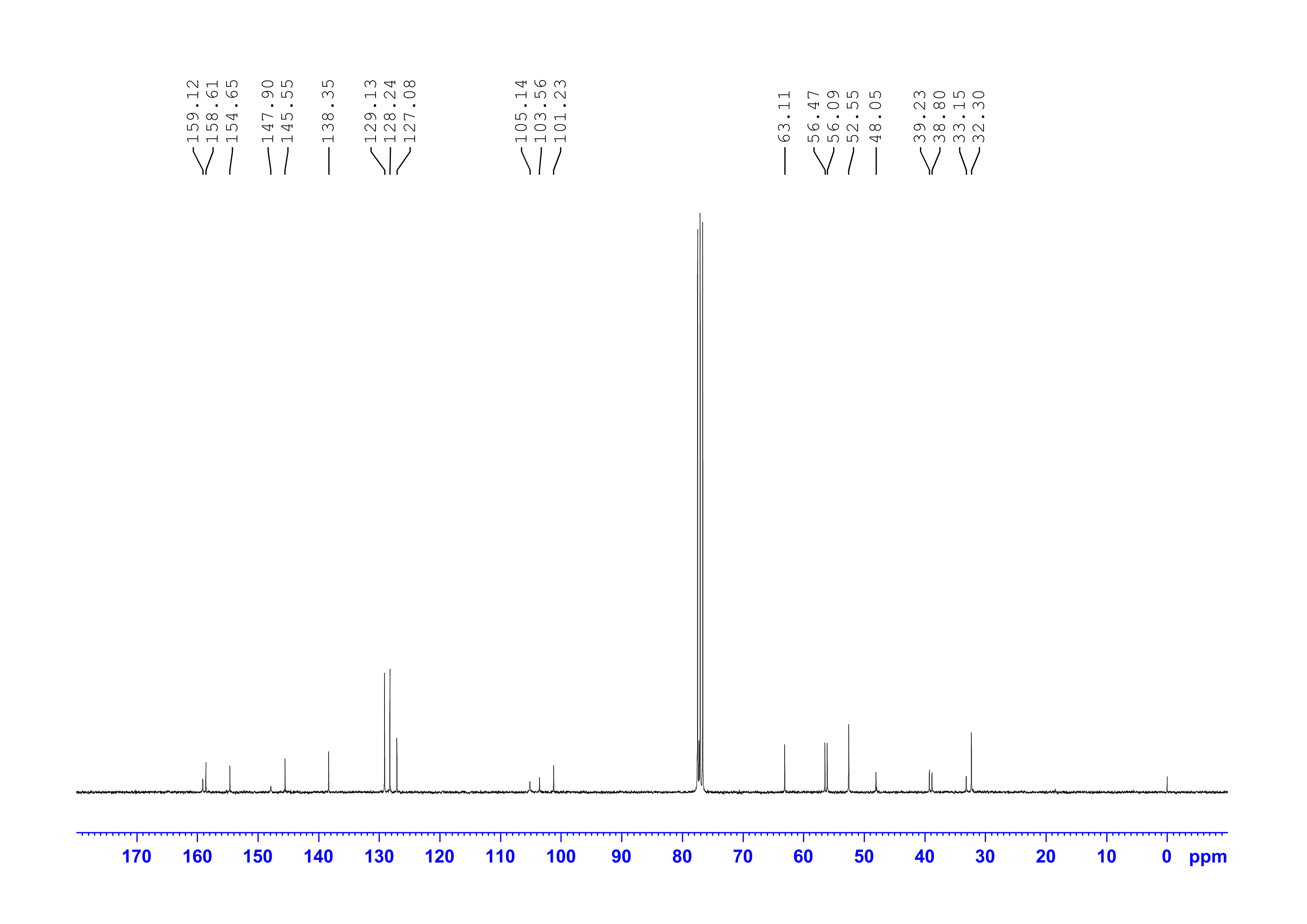
**

**^1^H NMR (Compound 14d)**

**
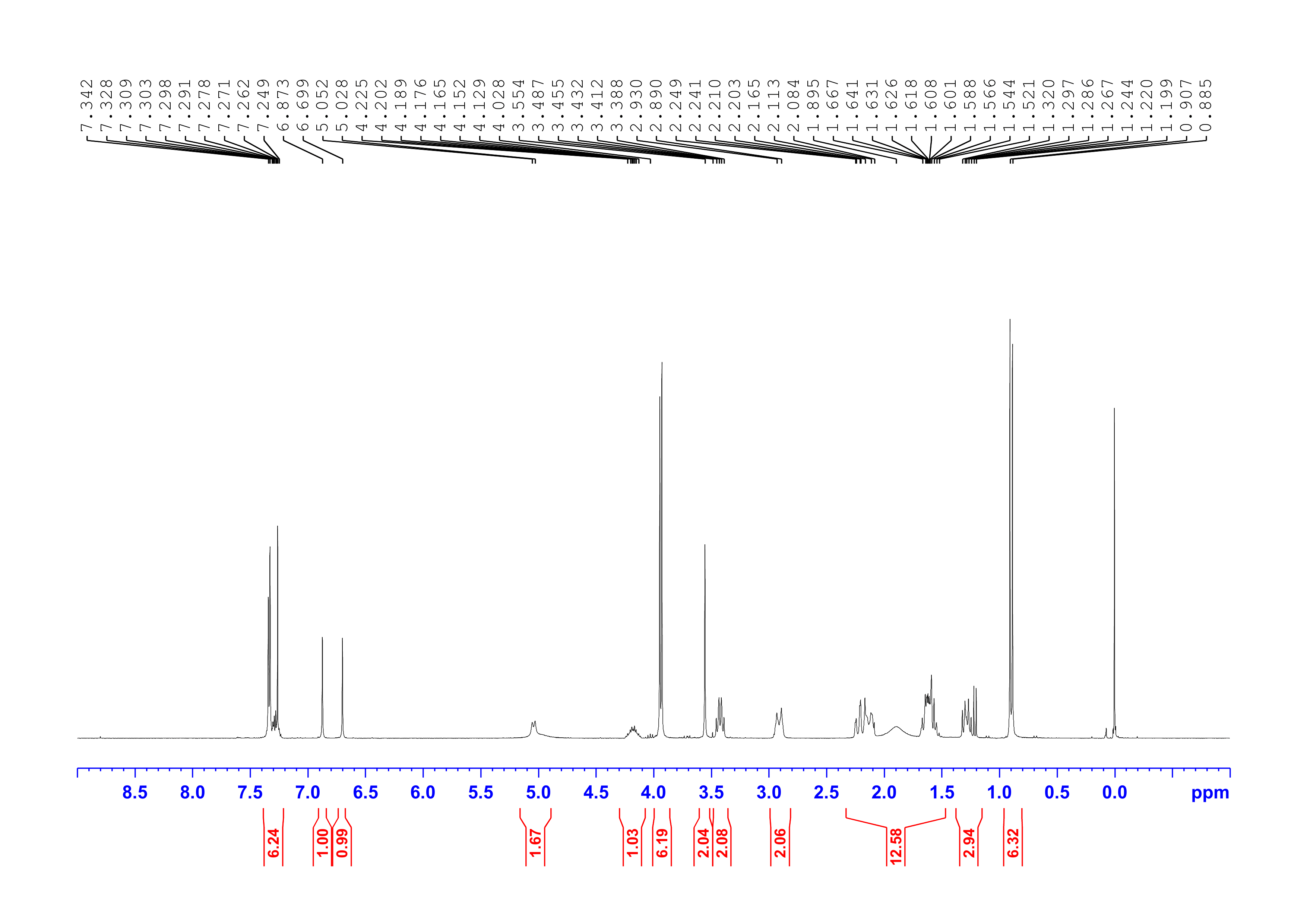
**

**^13^C NMR (Compound 14d)**

**
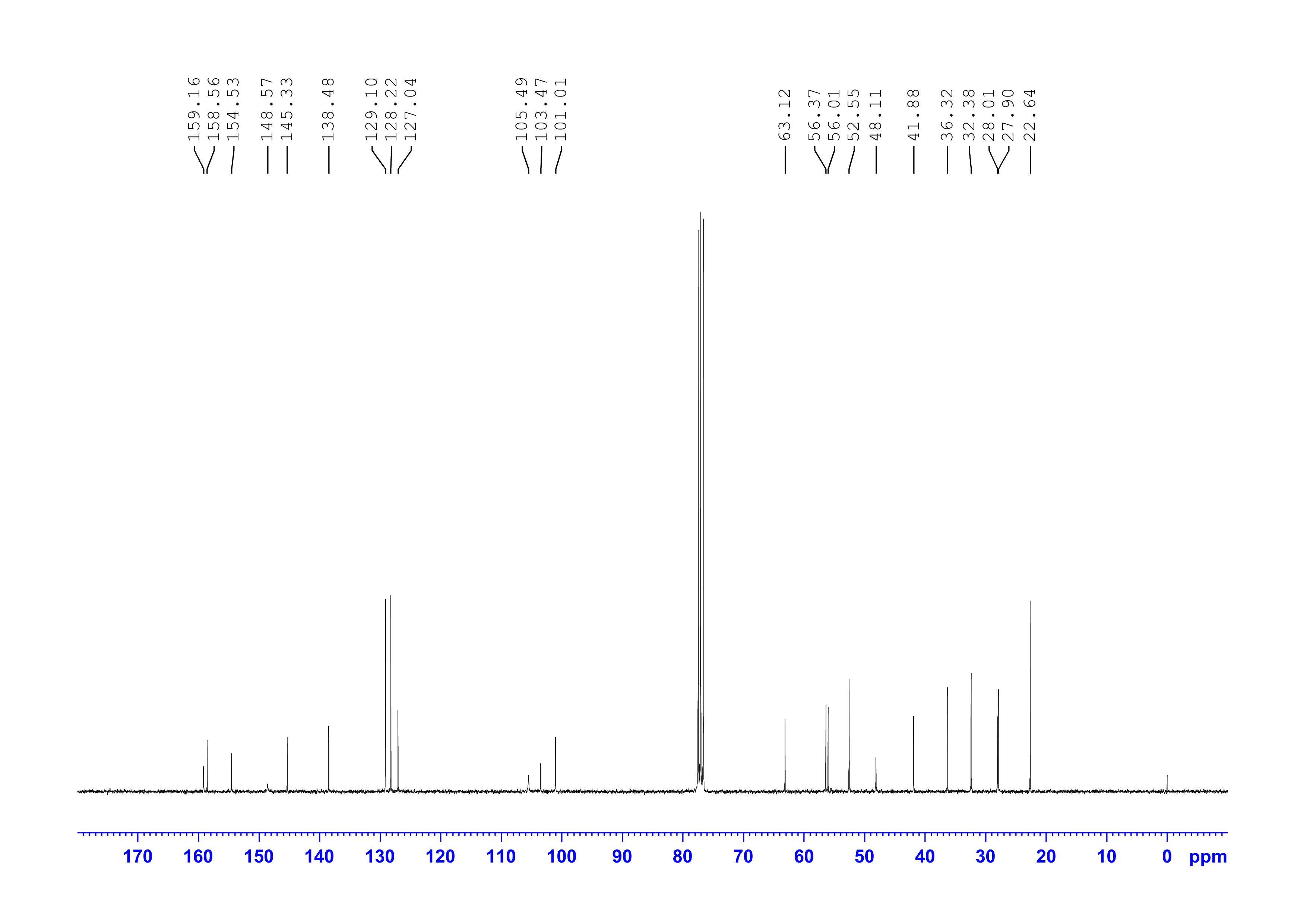
**

**^1^H NMR (Compound 15a)**

**
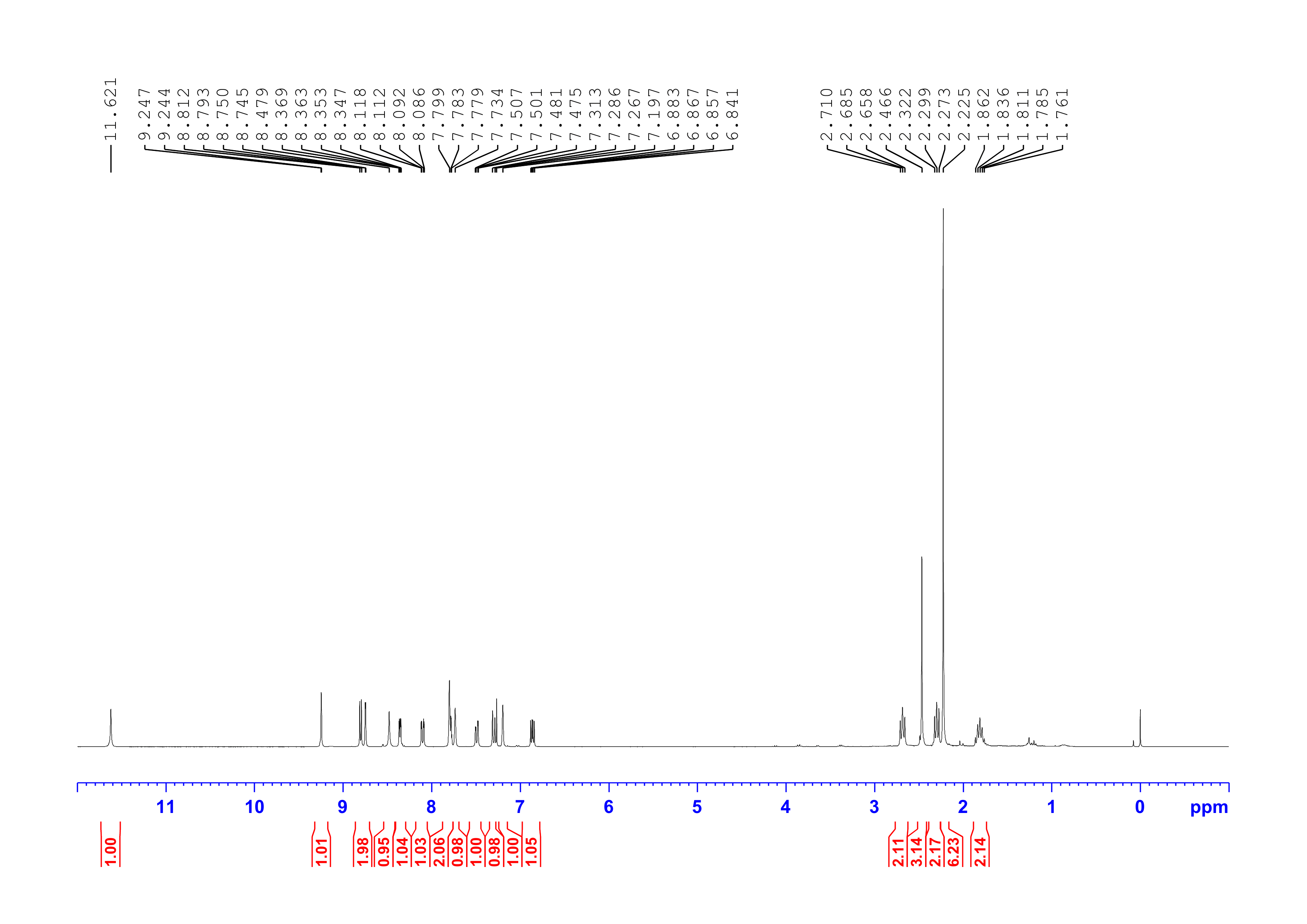
**

**^13^C NMR (Compound 15a)**

**
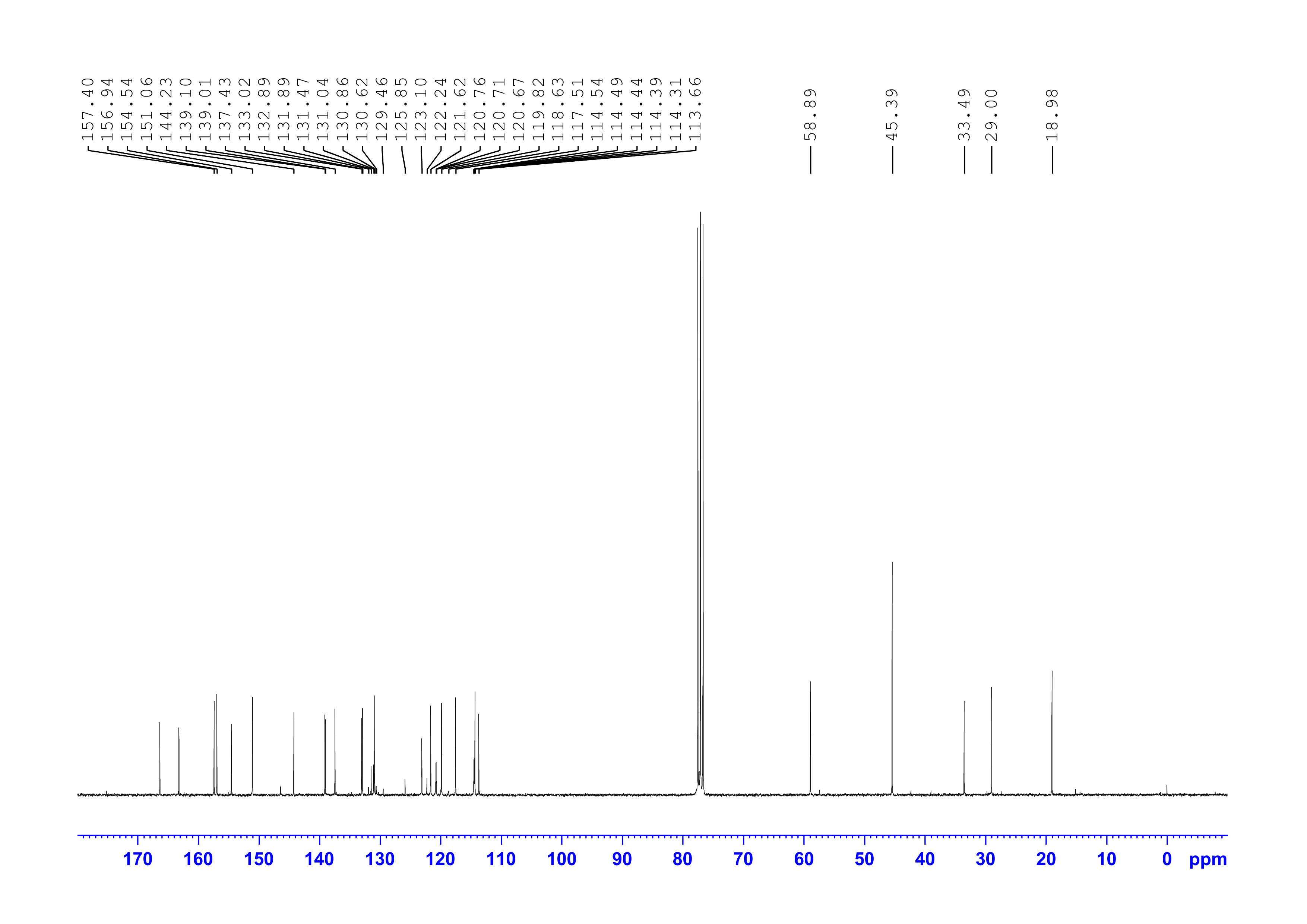
**

**^1^H NMR (Compound 15b)**

**
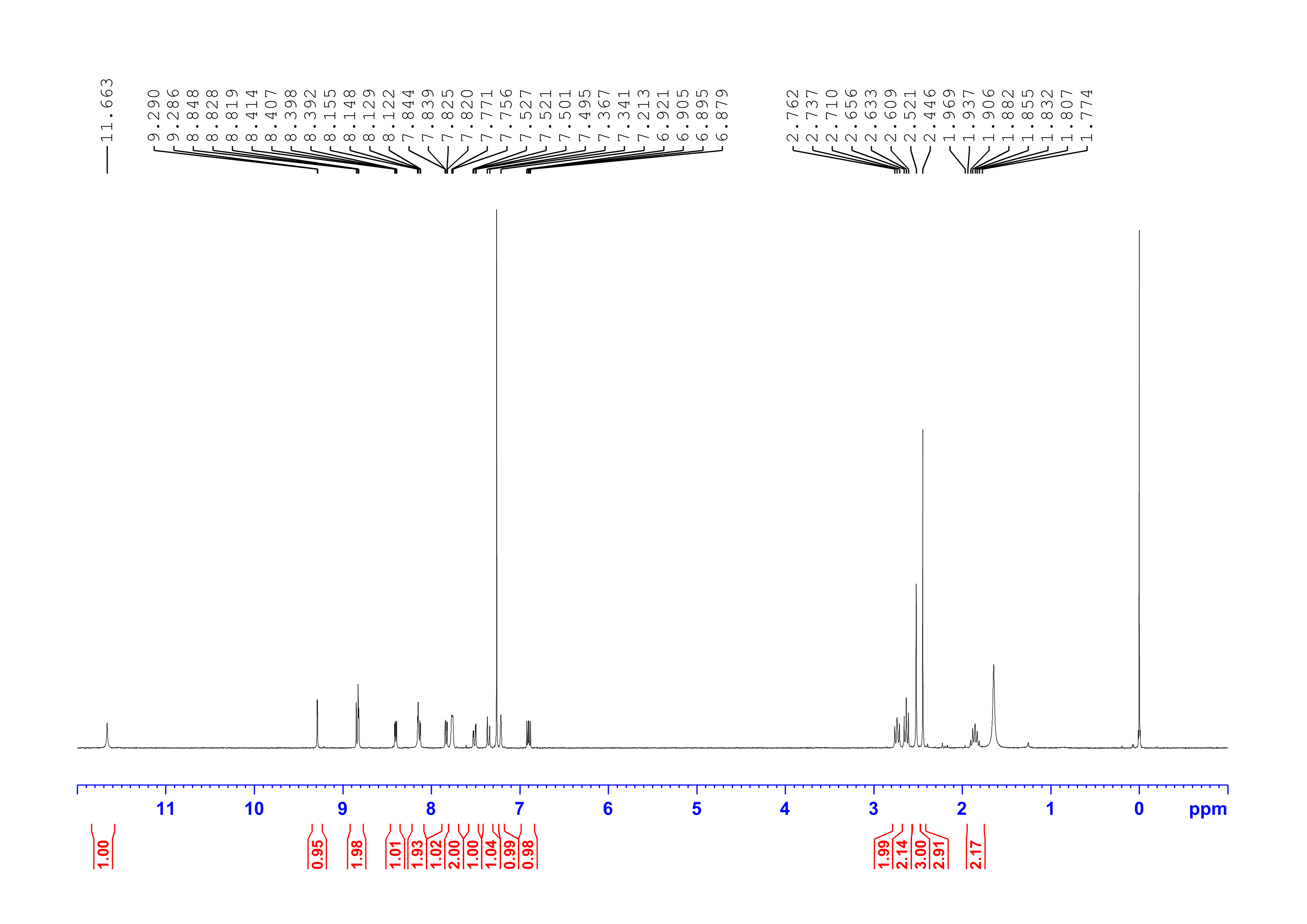
**

**^13^C NMR (Compound 15b)**

**^
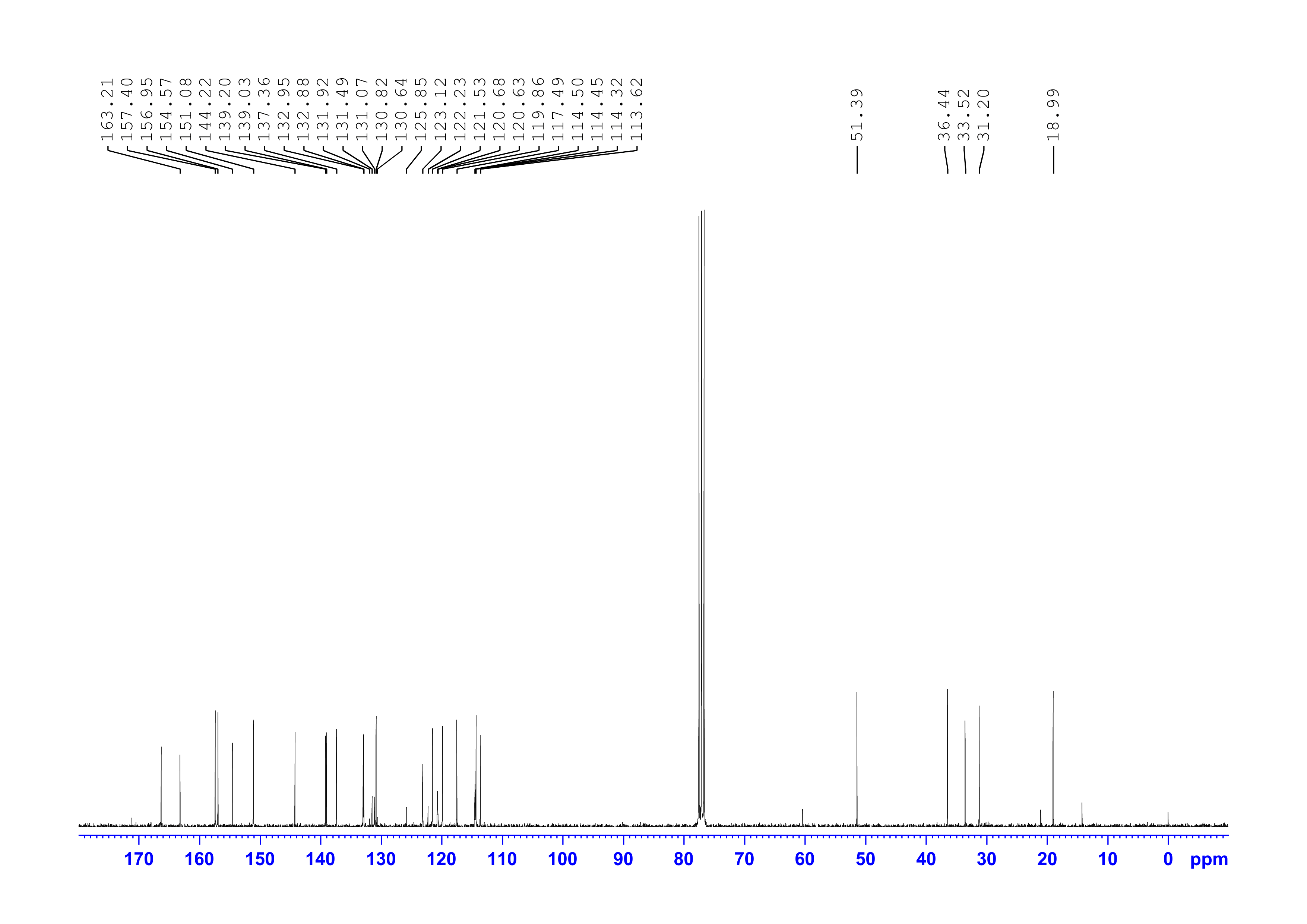
^**

**^1^H NMR (Compound 15c)**

**
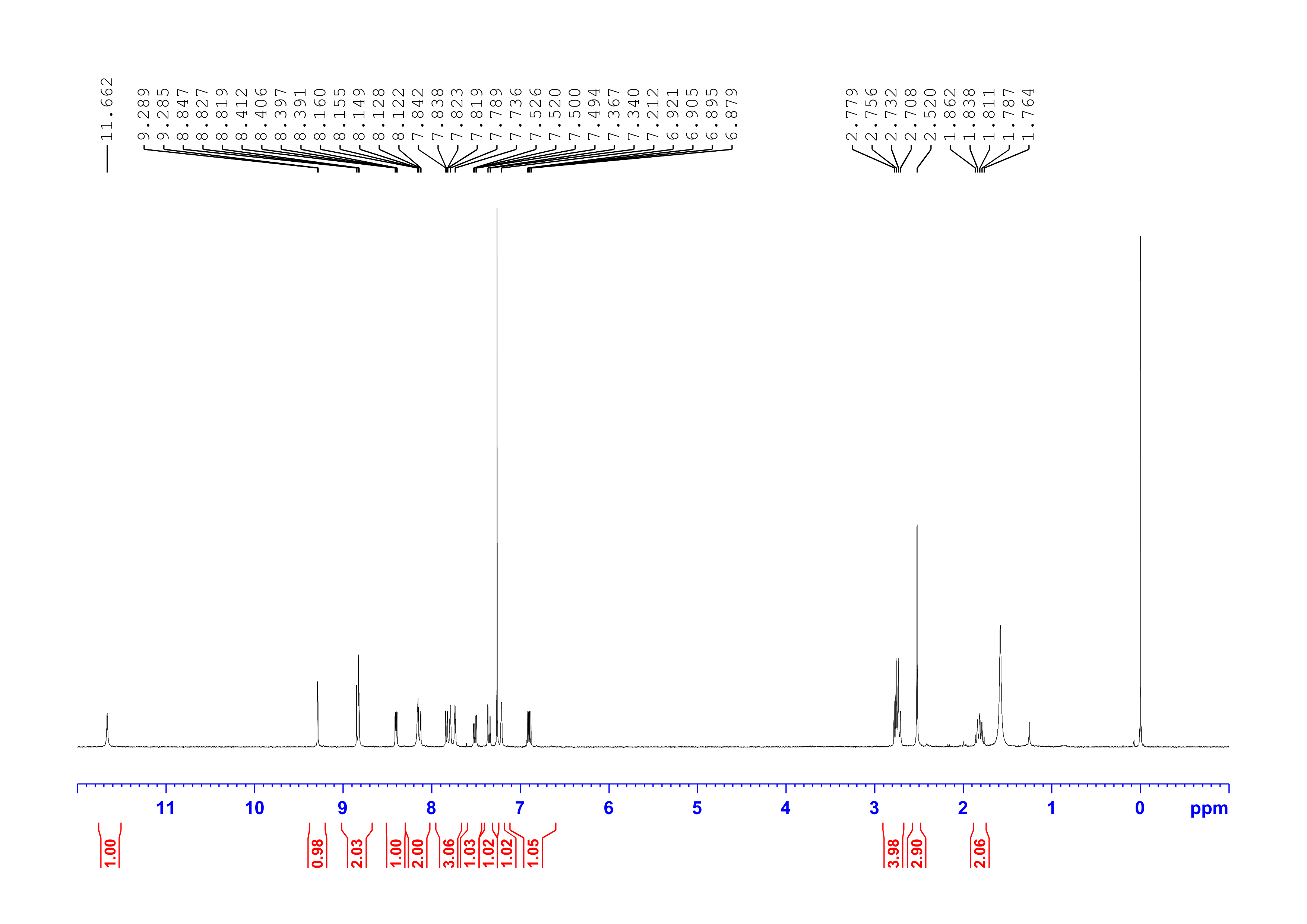
**

**^13^C NMR (Compound 15c)**

**
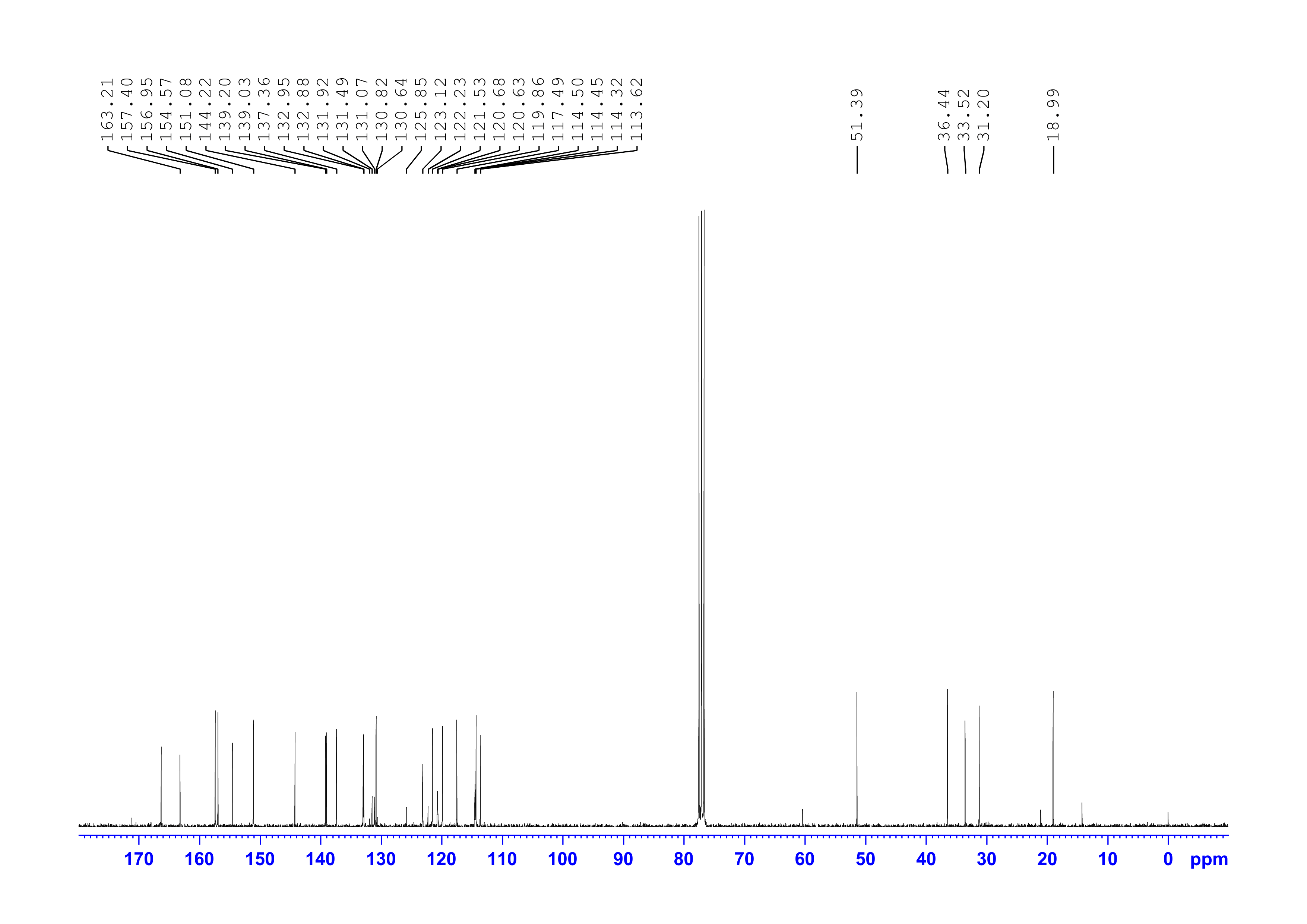
**

**^1^H NMR (Compound 15d)**

**
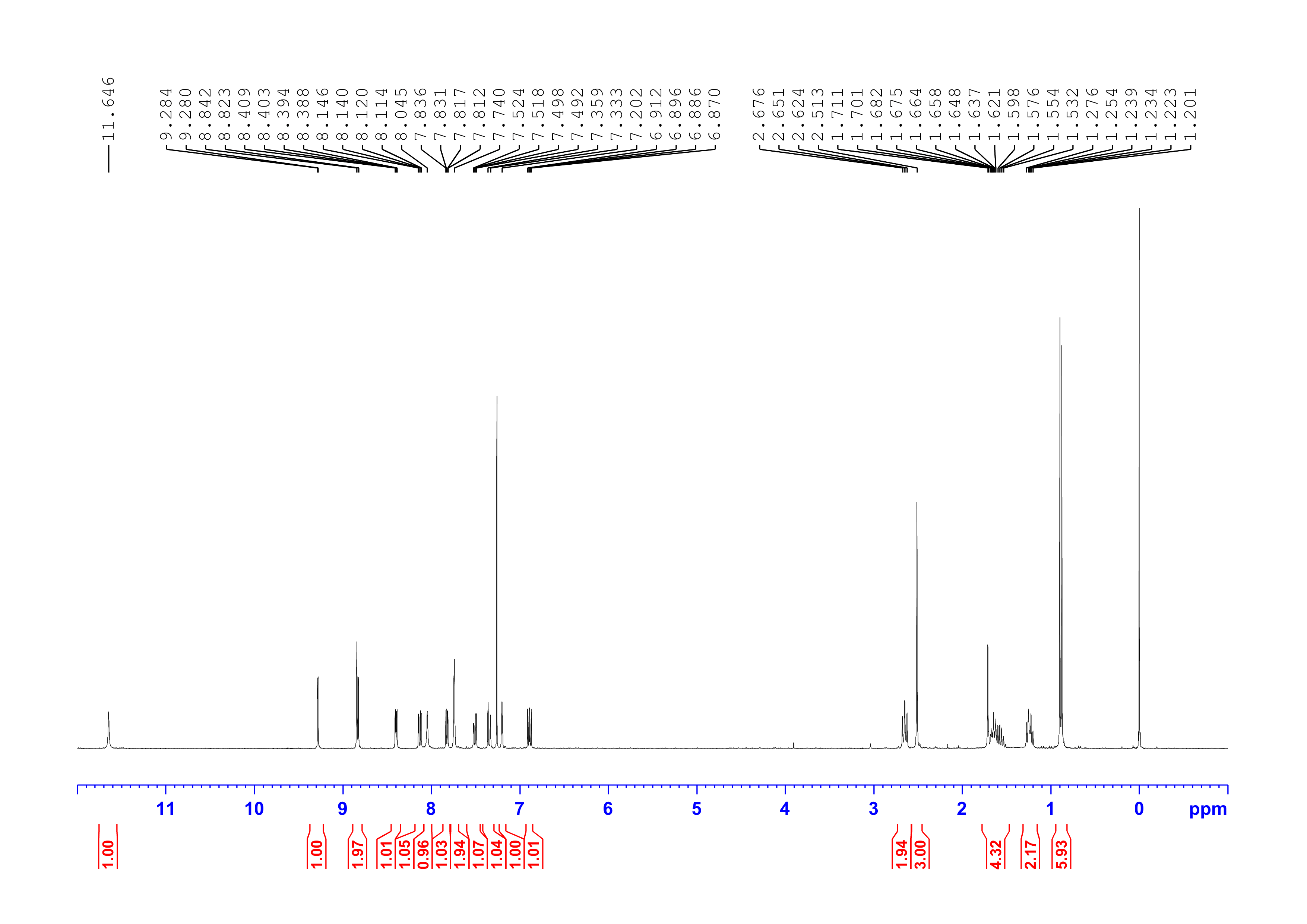
**

**^13^C NMR (Compound 15d)**

**
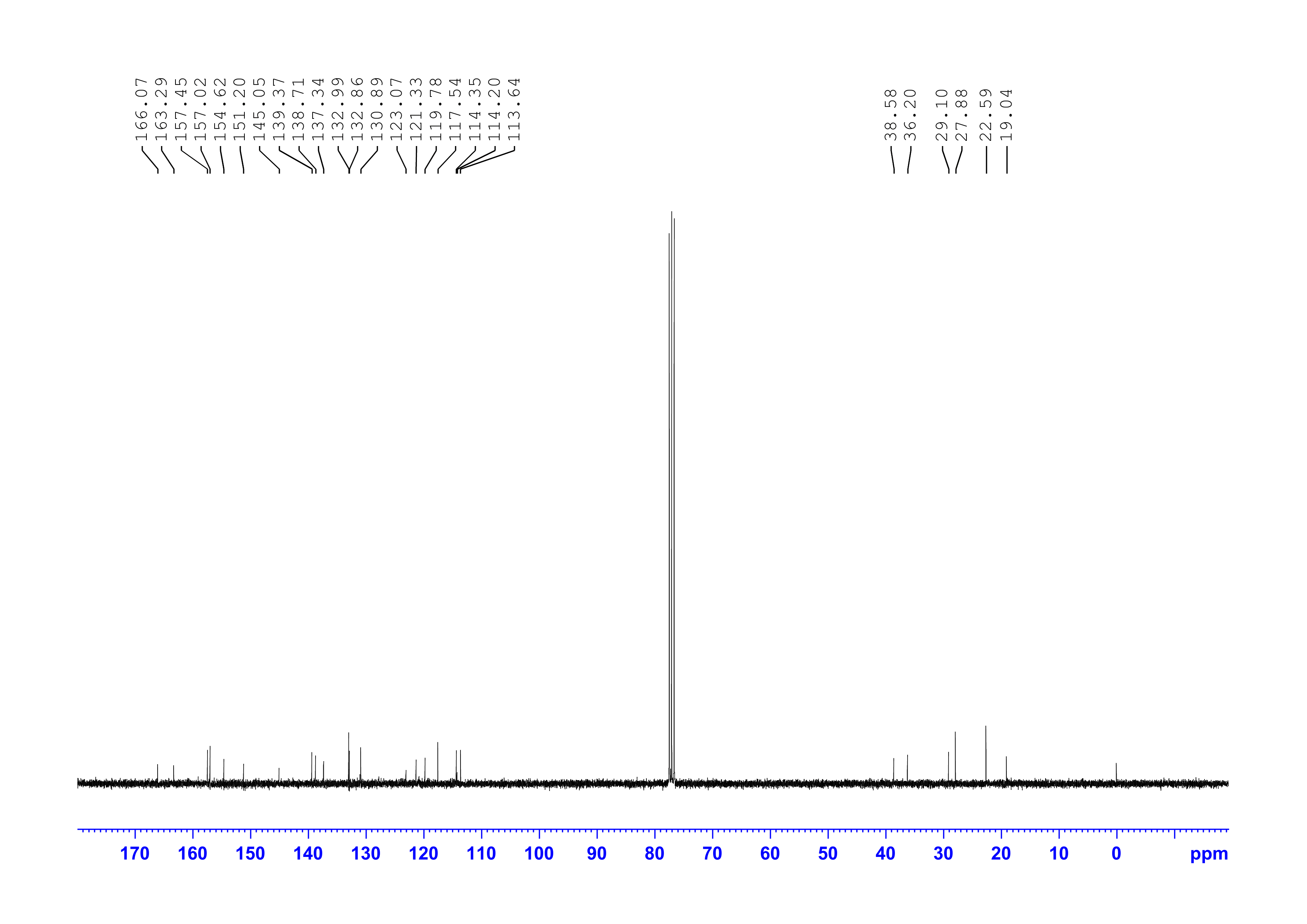
**

**SI References**

1. Hodous, B. L., et al. Evolution of a highly selective and potent 2-(pyridin-2-yl)-1,3,5-triazine Tie-2 kinase inhibitor. *J. Med. Chem.* **50**, 611–626 (2007).
2. Lukasik, P. M., et al. Synthesis and biological evaluation of imidazo[4,5-b]pyridine and 4-heteroaryl-pyrimidine derivatives as anti-cancer agents. *Eur. J. Med. Chem.* **57**, 311–322 (2012).
